# Measuring What Latent Fingerprint Examiners Consider Sufficient Information for Individualization Determinations

**DOI:** 10.1371/journal.pone.0110179

**Published:** 2014-11-05

**Authors:** Bradford T. Ulery, R. Austin Hicklin, Maria Antonia Roberts, JoAnn Buscaglia

**Affiliations:** 1 Noblis, Falls Church, Virginia, United States of America; 2 Latent Print Support Unit, Federal Bureau of Investigation Laboratory Division, Quantico, Virginia, United States of America; 3 Counterterrorism and Forensic Science Research Unit, Federal Bureau of Investigation Laboratory Division, Quantico, Virginia, United States of America; University of Catania, Italy

## Abstract

Latent print examiners use their expertise to determine whether the information present in a comparison of two fingerprints (or palmprints) is sufficient to conclude that the prints were from the same source (individualization). When fingerprint evidence is presented in court, it is the examiner's determination—not an objective metric—that is presented. This study was designed to ascertain the factors that explain examiners' determinations of sufficiency for individualization. Volunteer latent print examiners (n = 170) were each assigned 22 pairs of latent and exemplar prints for examination, and annotated features, correspondence of features, and clarity. The 320 image pairs were selected specifically to control clarity and quantity of features. The predominant factor differentiating annotations associated with individualization and inconclusive determinations is the count of corresponding minutiae; other factors such as clarity provided minimal additional discriminative value. Examiners' counts of corresponding minutiae were strongly associated with their own determinations; however, due to substantial variation of both annotations and determinations among examiners, one examiner's annotation and determination on a given comparison is a relatively weak predictor of whether another examiner would individualize. The extensive variability in annotations also means that we must treat any individual examiner's minutia counts as interpretations of the (unknowable) information content of the prints: saying “the prints had N corresponding minutiae marked” is not the same as “the prints had N corresponding minutiae.” More consistency in annotations, which could be achieved through standardization and training, should lead to process improvements and provide greater transparency in casework.

## Introduction

Latent print examiners compare latents (friction ridge impressions from the fingers, palms, or feet of an unknown subject) to exemplars (prints deliberately collected from known subjects), to determine whether the two prints originated from the same source. (See Glossary, Appendix S1.) Testimony on fingerprint evidence presented in court is based on the examiner's expert opinion, not an objective metric: “The criteria for absolute identification in fingerprint work are subjective and ill-defined. They are the product of probabilistic intuitions widely shared among fingerprint examiners, not of scientific research.” [1] Because of the societal implications of fingerprint testimony, it is important to understand what examiners consider sufficient information for individualization determinations.

An examiner's determination of individualization is that examiner's assessment that the information in the two prints is in sufficient agreement to conclude that they came from the same source. Examiners are highly accurate when they individualize [2]–[3], but they do not always agree whether the evidence supports individualization, as opposed to exclusion (different sources) or inconclusive [2], [4]–[5]. There are two aspects to the sufficiency criteria: the examiner's assessment of the content of the prints, and how much agreement is sufficient (given the clarity, distortion, and the rarity of the configurations of the features); neither is standardized.

Policies and procedures for latent print examination vary within and among countries. For example, in some countries, a minimum minutia count (“point standard”) is used as a criterion for individualization: a 2011 survey of 73 countries by INTERPOL found that 44 countries use a point standard, 24 of which require a minimum of 12 minutiae [6]. Various papers have indicated that a minimum minutia threshold is problematic [7]–[9]. The U.K. and most agencies in the U.S. previously used minutia count standards but abandoned them in favor of a nonnumeric, holistic approach [10]–[11]. In 1973, the International Association for Identification resolved that there was no basis for requiring a “pre-determined minimum number of friction ridge characteristics” for individualization [12].

In the holistic approach, an examiner's individualization determination is based on that examiner's assessment of the quantity and clarity of corresponding features, their relationships, and their specificity [13]–[14]. The fingerprint examination process is called ACE [15]–[16]: Analysis of the latent print (interpretation based on how it was deposited, developed, etc.), side-by-side Comparison of the two prints (observation of (dis)similarities), and Evaluation (determining whether the (dis)similarities are sufficient to support a conclusion). ACE relies upon the examiner's skills, training and experience, not upon formal criteria. In the absence of such criteria, the only available method for assessing whether an individualization is more appropriate than an inconclusive determination for a particular comparison is by consensus among examiners [17]. Compounding this issue is the fact that there are no generally accepted, rigorous definitions of features or clarity, and therefore no generally accepted systematic approaches to indicate confidence in features, to define ridge detail (level-3) features, or even consistent definitions of what exactly constitutes a minutia. The lack of such rigorous definitions and systematic approaches contributes to a lack of reproducibility (interexaminer agreement) and repeatability (intraexaminer agreement) of which features are annotated by examiners [18]–[21] and complicates attempts to develop quantitative approaches for sufficiency. *Data Format for the Interchange of Fingerprint, Facial, & Other Biometric Information* (ANSI/NIST-ITL 1-2011) [22] and *Markup Instructions for Extended Friction Ridge Features* [23] provide a standard means for the definition and exchange of forensic friction ridge feature data, but this recent advance is not yet widely used in operational casework nor has its effectiveness in casework been evaluated.

There have been a number of attempts over more than a century to more precisely articulate and standardize the procedures by which examiner reach determinations (surveys in [24]–[25]). Some of this research has been successfully incorporated into the development of Automated Fingerprint Identification Systems (AFISs), which are effective tools in matching finger- and palmprints in very large databases. For latents, AFISs generate candidates for human examiners to compare, and do not make automated decisions [26]–[28]—for exemplars, which are generally larger, higher quality, and less distorted than latents, AFISs can make fully automatic determinations without involving human examiners for all but the poorest quality images [29]. Another branch of research proposes the use of statistical models (e.g., [30]–[34], [25]) to augment or replace the determinations of latent print examiners with probabilistic estimates of the strength of evidence; these models are not yet generally accepted for operational use.

What constitutes sufficiency for an examiner to reach an individualization determination is a critical question that has been the subject of extensive discussion and debate for many years; recently, this question has received increased attention as a result of critiques of the forensic sciences [35]–[39], a series of legal challenges to the admissibility of fingerprint evidence in the U.S. (e.g., [40]–[42]), and publicized errors [43]–[44]. In order to understand the bases for examiners' determinations, we designed an experiment to investigate the relationship between the clarity and quantity of features in fingerprints and examiners' determinations. In a previous study [2], [4], we evaluated the accuracy, repeatability, and reproducibility of examiners' determinations without attempting to determine how those determinations were made (“black box” approach). In this experiment, practicing latent print examiners annotated features, clarity, and correspondences in latent and exemplar fingerprints to document what they saw when performing examinations (“white box” approach).

This report focuses on the question of sufficiency for individualization: how much information do examiners require in order to make an individualization rather than inconclusive determination? Subsequent reports will address other results from this White Box study, including differences between Analysis and Comparison markup. As part of our investigation, we sought to determine what information must be accounted for when describing the decision threshold, how the reproducibility of individualizations is associated with annotations, and to what extent disagreements among examiners arise from differing criteria as to what constitutes sufficiency vs. differing interpretations of the prints.

## Materials and Methods

### Ethics Statement

The collection of fingerprints from human subjects was approved by the FBI Laboratory Institutional Review Board and the Noblis Institutional Review Board. Use of latent print examiners in the study was approved by the FBI Laboratory Institutional Review Board, and written informed consent was obtained from all participating examiners.

### Test procedure

**Figure 1:**
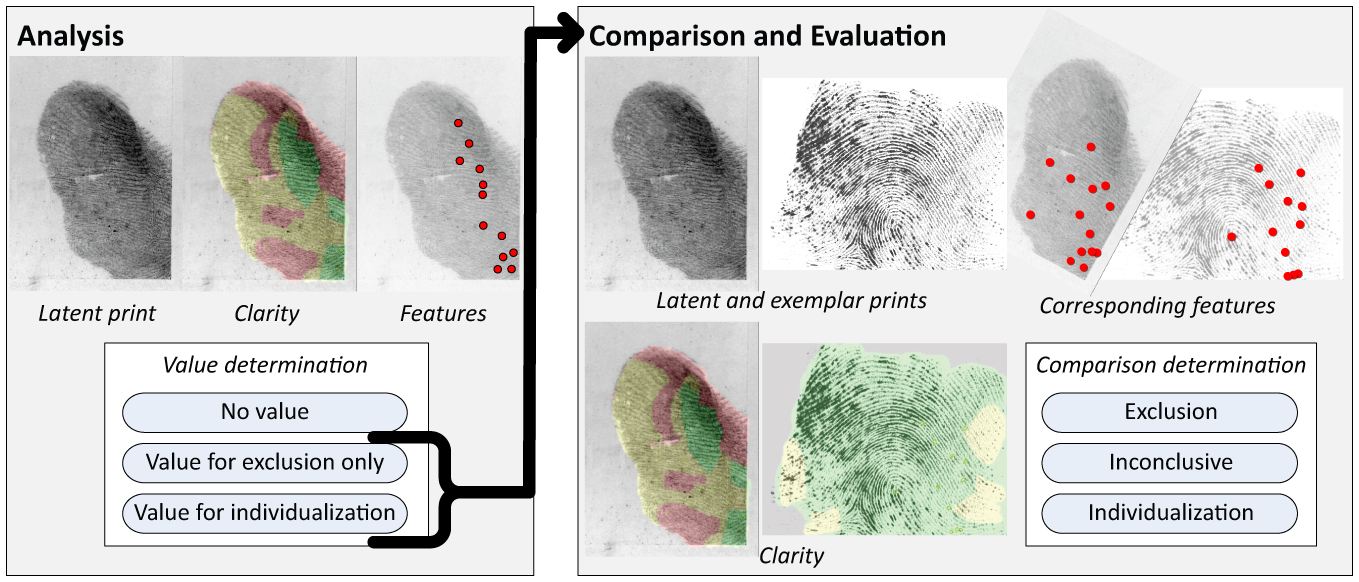
Test workflow. Each examiner was assigned a distinct, randomized sequence of latent-exemplar image pairs. For each pair, the latent was presented first for a value decision. If the latent was determined to be no value, the test proceeded directly to the latent from the next image pair; otherwise, an exemplar was presented for comparison and evaluation.

The test procedure was designed to correspond to that part of casework, in which a single latent is compared to a single exemplar print (latent-exemplar image pair). The test workflow (Figure 1) conformed to the prevailing latent print examination methodology known as Analysis, Comparison, Evaluation, and Verification (ACE-V) [15], [16]. During the Analysis phase, only the latent was presented; the examiner annotated clarity and features and recorded a value determination: value for individualization (VID), value for exclusion only (VEO), or no value (NV). If VID or VEO, the examiner proceeded to the Comparison/Evaluation phase, in which the exemplar was presented for side-by-side comparison with the latent: the examiner annotated clarity and recorded a value determination for the exemplar; compared the two images and further annotated the features to indicate correspondences and discrepancies; recorded a comparison determination (individualization, exclusion, or inconclusive); and indicated the difficulty of the comparison. The Verification phase was not addressed in this study. Examiners could review and revise their work prior to submitting their results. Examiners were free to modify the annotation and value determination for the latent after the exemplar was presented, but any such changes were recorded and could be compared with their Analysis responses.

**Figure 2:**
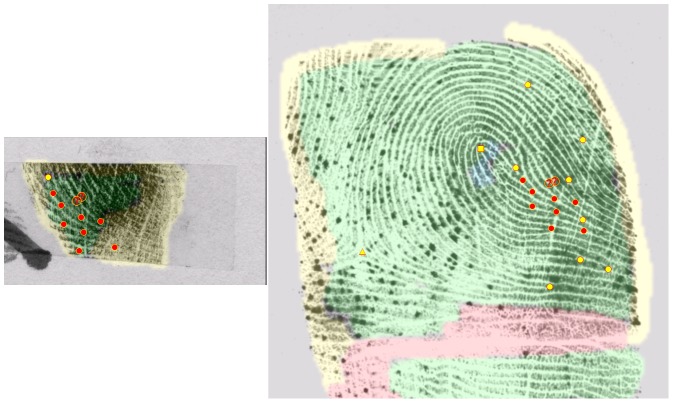
Example annotation of a mated image pair. Corresponding features are indicated here in red, unassociated features in yellow, and debatable correspondences with question marks. This examiner marked 8 corresponding minutiae, 2 debatable correspondences, individualized, and assessed it as very difficult. Determinations by the 9 examiners assigned this image pair: 5 NV, 4 VID (1 changed to NV during Comparison); 1 inconclusive, 2 individualization.

The software application used for our experiment is a variant of the FBI's Universal Latent Workstation's Comparison Tool [45]. It included tools for annotating the fingerprints, simple image processing, and recording the examiners' determinations. Fingerprint annotations complied with the ANSI/NIST-ITL 1-2011 standard [22] (using Extended Feature Set features); the test instructions were derived in part from [23]. In the Analysis phase, the examiners provided the following annotations pertaining to the latent: local clarity map (produced by “painting” the images using six colors denoting defined levels of clarity [46], [22]); locations of features; types of features (minutiae, cores, deltas, and “other” points (nonminutia features such as incipient ridges, ridge edge features, or pores)); and value determination (VID, VEO, or NV). If the latent print was determined to be VEO or VID, the examiner provided the following annotations during the Comparison/Evaluation phase (Figure 2): latent and exemplar clarity; latent and exemplar features, as well as correspondences (definitive and debatable) and discrepancies; latent and exemplar value determinations; comparison determination (individualization, exclusion, or inconclusive); and comparison difficulty (very easy/obvious, easy, moderate, difficult, very difficult).

Participants were instructed in the test objectives, procedures, and tool usage through a short video (Video S1) and a detailed instruction document (Appendix S20). Prior to taking the test, they were asked to complete practice exercises to become familiar with the software and instructions. We estimated that the test would take about 8 to 12 hours to complete. Participants were permitted to take as long as they wished to complete the test over a period of approximately one month (numerous extensions were granted). Actual completion times varied substantially among examiners (see Appendix S6).

Participation was open to practicing latent print examiners and included a broad cross-section of the fingerprint community. A total of 170 latent print examiners participated: 90% were certified (or qualified by their employers) as latent print examiners; 82% were from the U.S. Most of the participants were volunteers, but some were required or requested to participate by their employers. Participants were assured that their results would remain anonymous; a coding system was used to ensure anonymity during Analysis and in reporting. Participant survey responses are summarized in Appendix S7. The participants reported a range of prior experience in performing latent print comparisons on computer screens (Appendix S7, question #11); lack of familiarity with computer-based comparisons may have had an impact on the performance of some of the examiners in this study. For further details on Methods and Materials, see Appendices S2, S3, and S4.

### Fingerprint selection and assignments

Fingerprints were collected under controlled conditions for research and selected from operational casework. Latent-exemplar image pairs collected under controlled conditions are ***known*** to be mated (known to be from the same source) or nonmated (known to be from different sources), whereas the terms “individualization” and “exclusion” refer to examiners' ***determinations*** as to whether the prints are from the same source. In our previous Black Box study [2], [4], in which the focus was on the correctness of the determinations, we only used images collected under controlled conditions because it was critical that the mating be known definitively. In this study, it was less critical that the mating be known with certainty because the objective was to investigate the bases for examiners' determinations, not their correctness. Here, in order to increase the variety of attributes (such as substrates, matrices, and processing methods), we included prints from operational casework. Mating of casework prints was established through the use of multiple additional corroborating latents and exemplars that were available in these cases; mating was not established solely through the use of the latents presented in the test.

Nonmated pairs were selected to result in challenging comparisons. They were prepared by down-selecting among exemplar prints returned by searches of over 58 million subjects (580 million distinct fingers) in the FBI's Integrated AFIS (IAFIS), and among neighboring fingers from the same subject; neighboring index, middle, or ring fingers from a subject often have similar fingerprint pattern classifications and therefore are more likely to be similar than two random fingerprints.

Although the fingerprints actually came from casework or were collected to resemble examples from casework, the sampling strategy was not designed to yield a mix of prints that would be representative of typical casework. Instead, the fingerprint pairs were selected to vary broadly over a four-dimensional design space: number of corresponding minutiae, image clarity, presence or absence of corresponding cores and deltas, and complexity (based on distortion, background, or processing). These four dimensions were selected to evaluate their effects on individualization determinations. The sampling method emphasizes pairs with low counts of corresponding minutiae in order to focus on the threshold between individualization and inconclusive, with the implication that our results would show lower interexaminer reproducibility than would be typical in casework.

Through a preliminary screening process, fingerprints were assigned to bins representing combinations of levels for each dimension (see Table 1). Each bin (48 bins for mated pairs, 16 bins for nonmated pairs) was populated with a sample of three to six pairs of fingerprints, depending on the difficulty of obtaining suitable samples. This design resulted in a total of 320 image pairs (231 mated and 89 nonmated pairings), including 301 distinct latents.

**Table 1:** Design dimensions used for data selection.

Dimension	Mates (48 bins)	Nonmates (16 bins)
**Corresp. minutiae**	1–4, 5–8, 9–12, 13–20	0-4, 5–8, 9–12, 13–20
**Corresp. cores or deltas**	Yes, No	Yes, No
**Corresp. clarity**	Low, Medium, High	Low, High
**Complexity**	Low, High	—

Each pair of fingerprints was assigned to one of 64 bins indicating the true mating and factor level for each of four dimensions. The factor levels for mated pairs describe the corresponding information available in the area of overlap. Nonmated pairs were described based on “apparent correspondences.”

The preliminary bin assignments were not intended for final analysis, which would rely instead upon the measures obtained from test participants. In operational data, these four dimensions tend to be correlated; we controlled the dimensions independently to be able to observe separate effects. This approach over-represents some types of prints that would be uncommon and under-represents others that are common.

Based on preliminary estimates of the time that would be required of participants, we assigned 22 image pairs to each examiner. In order to concentrate the test design on sufficiency for individualization, each examiner was assigned 17 mated pairs and 5 nonmated pairs. The emphasis on mated pairs was not revealed to participants; the true proportions would have been obscured through NV determinations, inconclusive determinations, and erroneous exclusions.

The assignments of fingerprint images to examiners followed a randomized incomplete block design (with examiners as blocks, image pairs as factor levels), balanced to the extent possible: separate designs were implemented for mated and for nonmated pairs. The data and experimental design are discussed further in Appendix S3. The number of examiners per image pair was selected as a compromise: more examiners per image pair increases the ability to measure interexaminer reproducibility, whereas fewer examiners per image pair increases the total number of images in the study thereby increasing the ability to measure a greater variety of image attributes. The final design allowed us to measure individualization rates for each image pair and each examiner. The final design also allowed us to explore the importance of specific image attributes, including interaction effects among image attributes. The experimental design allows us to model both image and examiner effects on responses but is not sized to directly measure interaction effects between images and examiners. For example, an image-examiner interaction is present when an examiner has an average individualization rate overall yet is more likely than other examiners to individualize low-clarity images.

### Analysis data

The test yielded 3730 valid responses from the Analysis phase (170 examiners, mean 12.4 examiners per latent). Among these were 2796 *mated* pairs with valid responses from both phases (165 examiners, mean 12.1 examiners per image pair). A summary of test responses and examiner determinations is provided in Appendix S5.

In order to describe the decision boundary between individualization and inconclusive, we often restrict our attention to the 2671 mated pairs with inconclusive (including no value) or individualization determinations (i.e., omitting erroneous exclusions). We omit the exclusions because the decision criteria for exclusions and individualizations are distinct: an increase of corresponding information provides support for an individualization vs. an inconclusive determination, whereas an increase of discrepant or contradictory information provides support for an exclusion vs. an inconclusive determination. Exclusions may be based on pattern class information when there is insufficient information for individualization, or they may result from an examiner's determination that a single feature was discrepant despite otherwise having sufficient information for individualization.

Summary information for analyses was extracted from the examiners' annotations as detailed in Appendix S4. In this paper, our analyses of the annotated features are limited to counts of those features; we plan further analyses of the features (e.g., by location) in future papers. For comparisons that resulted in three or more corresponding features, each examiner's clarity maps for the latent and exemplar were superimposed using a thin-plate spline deformation model (method detailed in [47]); a “corresponding clarity” map was then defined as the minimum clarity at each location of the two superimposed maps, as described in [46]. Also, for each image and each image pair, the clarity maps from all examiners who were assigned that pair were combined to produce median clarity maps representing a group consensus, reducing the impact of outlier opinions and imprecision. Clarity measures, including various area measures and the “Overall Clarity” metric [46], were derived from each of the clarity maps (original, corresponding, and median).

## Results and Discussion

What constitutes sufficiency for individualization as opposed to inconclusive determinations? Here we explore the following aspects of that question: What is the association between examiners' annotations and their own determinations? What is the association between one examiner's annotation and another examiner's determination? What are the factors explaining the reproducibility of annotations and determinations among multiple examiners?

### Associations between examiners' annotations and their determinations

**Figure 3:**
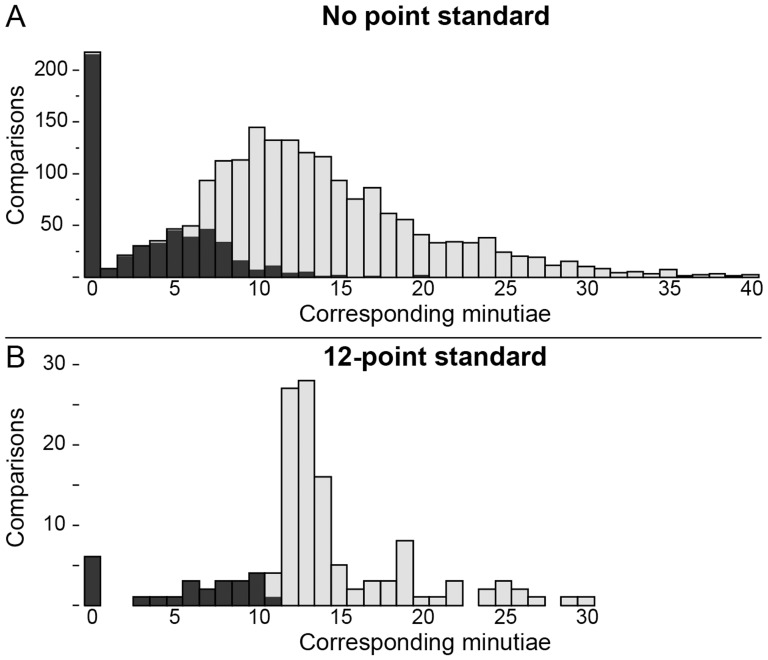
Associations of (A) minutia count and value determinations from analysis of the latent (n = 3730); (B) corresponding minutia count and determinations from comparison of latent and exemplar prints on mated data (n = 2796). In (B), 1.6% of determinations with 12 or more corresponding minutiae marked were not individualized. A few responses in (B) indicate NV with corresponding minutiae due to examiners changing their value determinations during Comparison.

The number of minutiae annotated by examiners is strongly associated with their own value and comparison determinations (Figure 3). Value is a preemptive sufficiency decision: NV indicates that any comparison would be inconclusive. For both value (Figure 3A) and comparison (Figure 3B) determinations, a count of seven minutiae is a tipping point between determinations: for any minutia count greater than seven, the majority of ***value*** determinations were VID, and for any ***corresponding*** minutia count greater than seven, the majority of *****comparison***** determinations were individualization (see also Appendix S8). Only sixteen individualization determinations (1% of all individualizations) had fewer than seven corresponding minutiae marked (detailed in Appendix S9); most of these can be explained as having additional corresponding features (either nonminutia features or “debatable” correspondences) or as invalid annotation (features were marked in both images but not the correspondences). These results are consistent with our previous findings on the sufficiency for value determinations [21], as well as those of other researchers: Budowle et al. [48] discussed an informal minimum threshold of seven minutiae for value determinations; Langenburg [20] observed that examiners were more likely to make VID determinations than not VID starting at about seven to eight minutiae, and the cross-over point for individualization was about eight to nine corresponding minutiae.

High minutia counts are not limited to VIDs and individualizations: there are high-count VEO determinations (ranging up to 27 minutiae) and high-count inconclusive determinations (up to 20 corresponding minutiae); the majority of these determinations are on prints with discontinuous areas or low-clarity minutiae. Figure 3B also shows erroneous exclusions (red): these occurred at a lower rate (5.5%) than in our previous Black Box study [2]; see discussion in Appendix S5.

Among nonmated image pairs (see Appendix S16), 89% had no corresponding minutiae marked, and few had more than seven corresponding minutiae marked. The single erroneous individualization (false positive) had 14 corresponding minutiae marked (the highest count among 582 comparisons of nonmated pairs); in Figure 3B we see that when 14 corresponding minutiae are marked, individualization is the typical determination for mated image pairs, and therefore the minutiae count for the false positive does not stand out as an anomaly.

We compared and evaluated a variety of models in order to assess the relative importance of factors associated with examiners' sufficiency decisions. These models are specifically focused on differentiating individualization vs. inconclusive determinations, and therefore omit erroneous exclusions (n = 2671).

To describe how well the various models fit our data, we report misclassification rate as a summary statistic: misclassification rates are calculated by treating the models as classifiers, where any estimated probability above 0.5 is interpreted as a predicted individualization; otherwise the model is interpreted as having predicted that the examiner did not individualize. Misclassification rate describes the effectiveness of our models in explaining examiner determinations; it is ***not*** referring to whether the determinations made by examiners are “correct” or “incorrect.” The misclassification rates are specific to the mix of data used here: examiners individualized the majority of the mated image pairs; therefore, the percentage of the 2671 mated pairs that were ***not*** individualized (38.1% were NV or inconclusive) defines the base misclassification rate when predicting individualizations (i.e., a model that assumes all mated pairs are individualizations would have a 38.1% misclassification rate).

We evaluated a variety of models relating the probability that an examiner would individualize to factors derived from that examiner's annotations. For example, we use the following logistic regression model to relate the probability of individualization to corresponding minutia count (*CMin*):
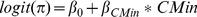
(Eq1a)where *π* is the probability of individualization for an examiner given *CMin* as marked by that examiner. This can also be expressed as
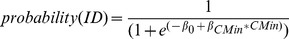
(Eq1b)


We use misclassification rate as a summary statistic when comparing the models. Misclassification rates are calculated by treating the models as classifiers, where the model is interpreted as having predicted an individualization if and only if the estimated probability is greater than 0.5. As reported in Table 2, the fitted model from Eq 1a predicts that an examiner who marks eight or more corresponding minutiae will individualize, resulting in a misclassification rate of 6.0% (2.4% of mated pairs were individualized with CMin≤7; 3.6% were not individualized with CMin≥8).

**Table 2:** Misclassification rates for models describing associations between annotations and individualization determinations by the same examiner.

Predictors	Description	Misclass
**None**	*(base rate)*	38.1%
**CD>0**	*whether any cores or deltas were marked*	38.1%
**Difficulty**	*very easy to very difficult*	24.1%
**OverallClarity**	*area metric derived from corresponding clarity map*	17.1%
**CMin>2**	*whether corresponding clarity map could be created*	13.6%
**CMin>0**	*whether any corresponding minutiae were marked*	12.6%
**CMin**	*count of corresponding minutiae*	6.0%
**CMin_yellow; CMin_green**	*CMin in areas of debatable and definitive clarity*	6.0%
**CMin; OverallClarity**		5.8%
**CMin; PtStd**	*whether examiner followed a 12-point standard*	5.7%
**CMin; Examiner**	*Which examiner; 166 degrees of freedom*	3.0%

(n = 2671 responses by 165 examiners on 231 mated pairs)

To assess the effectiveness of this model, we can compare this 6.0% misclassification rate to the base misclassification rate for this dataset, which results from a (trivial) model with no independent variables that always predicts the most prevalent examiner response. In this case, the base rate model predicts that examiners would always individualize mated pairs, and therefore it misclassifies responses whenever examiners actually determined NV or inconclusive (38.1%). Misclassification rate describes the effectiveness of our models in explaining examiner determinations; it is ***not*** referring to whether the determinations made by examiners are “correct” or “incorrect.” The misclassification rates reported here are specific to this dataset (the 2671 mated pairs, omitting erroneous exclusions) and are not estimates of operational rates.

Table 2 summarizes the performance of several models; see Appendix S12 for additional models and performance measures. Including additional modeling terms based on nonminutia annotations (clarity; cores, deltas, or other features; difficulty) did not markedly improve on the *CMin* model; this is a notable result given that we designed the study to measure the effect of these dimensions. This finding is consistent with our previous results regarding value determinations [21], and those of Neumann et al. [49].

We conducted analyses using analogous models associating annotations with latent value determinations (Appendix S11); those findings generally parallel our findings for comparison determinations, and confirm and expand upon our previous findings reported in [21].

The consistency with which participants annotated the image pairs had an impact on the strength of associations revealed by these models. For example, some examiners never marked cores or deltas, and the majority never marked “other” features (level-3 details). While markup of minutiae would be familiar to most examiners from AFIS searches and markup of cores and deltas from pattern classification, annotation of clarity and level-3 features would be novel to most participants. Corresponding clarity had a strong influence on sufficiency decisions, but that influence is subsumed by the count of corresponding minutiae: we presume that clarity is an important determinant of the selection of minutiae, but it has minimal additional effect after the minutiae are selected. Table 2 shows that most of the association captured by *OverallClarity* derives simply from whether or not the examiner marked corresponding minutiae: the *CMin>0* and *CMin>2* models explain much of the association; note that corresponding clarity maps can only be constructed if at least three corresponding points are marked.

The *CMin + Examiner* model includes a term indicating which examiner made the determination, resulting in a 3.0% misclassification rate. Specifically, the model becomes:
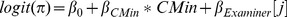
(Eq 2)


The *Examiner* terms model each examiner's individual individualization rate. The remaining 3.0% could be explained by lack of repeatability of the examiner's association between *CMin* and determinations, inconsistent usage of annotations among examiners, other interaction effects between examiners and image attributes, or limitations of the metrics used.

### Reproducibility of corresponding minutiae

**Figure 4:**
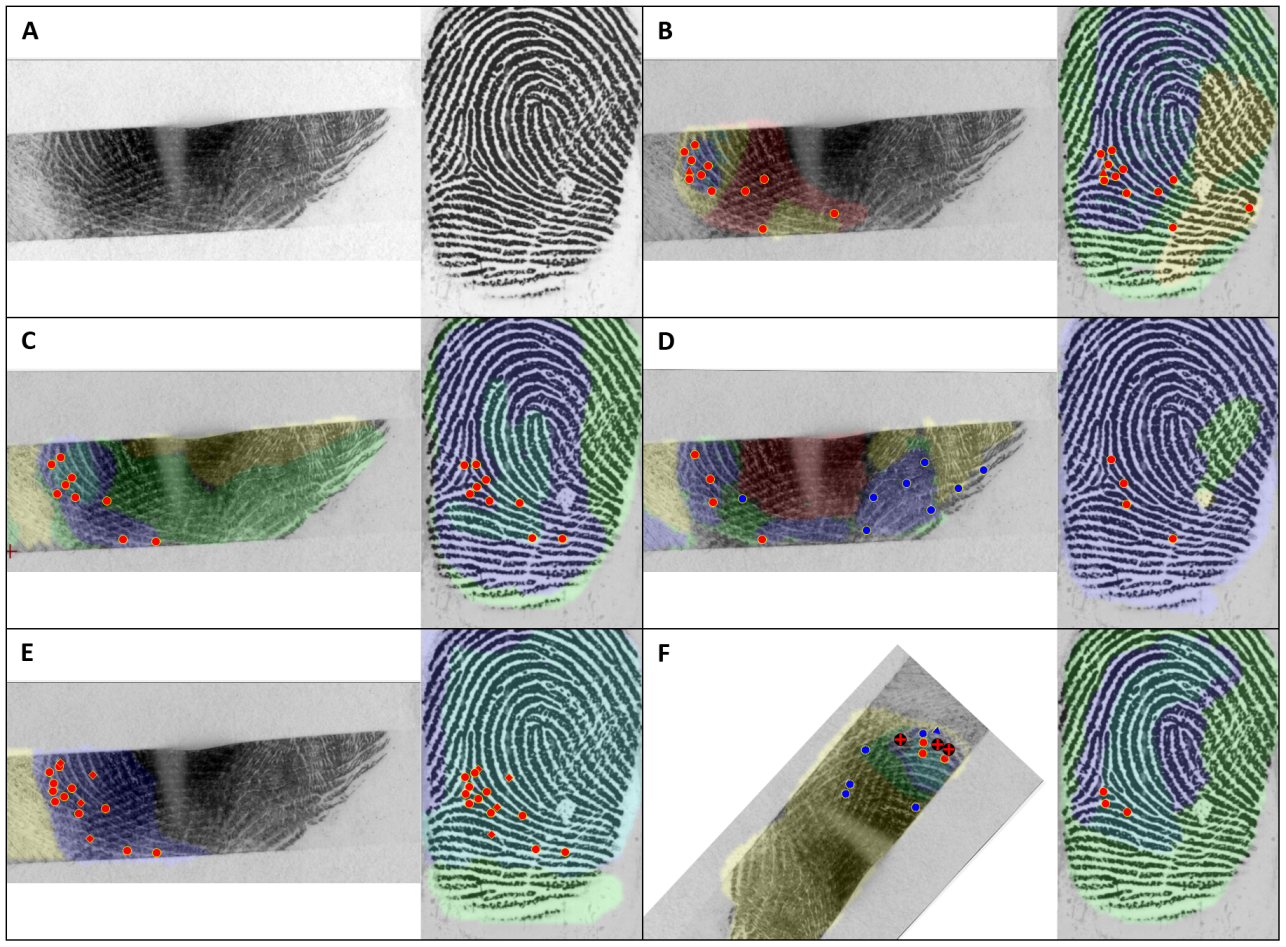
Example of a mated image pair (A), showing variations in annotation among five examiners (B–F). Corresponding points are shown here in red, unassociated in blue; minutiae as circles, deltas as triangles, other points as rhombuses; noncorresponding points as red Xs. Examiners B–E individualized; F excluded. Determinations by the 11 examiners assigned this image pair: 2 NV, 3 VEO (2 of which were changed to VID during Comparison), 6 VID; 1 inconclusive, 1 exclusion, 7 individualization.

While examiners' determinations are strongly associated with their own minutia counts, previous research has shown that minutia counts and determinations are not always highly reproducible among examiners [2], [4], [10], [20], [49], [21]. Figure 4 shows examples of interexaminer differences in annotations of corresponding minutiae, suggesting how some of the differences among examiners arise: examiners B, C, and E marked the features in a generally similar manner but differed on specific points (especially within the delta) and the extent of the areas they used in Comparison; examiner C changed value determination from VEO to VID during Comparison; examiner D individualized with only four corresponding minutiae but did not mark the delta or any of the features within the delta (improper annotation); examiner F misinterpreted the orientation, resulting in an erroneous exclusion.

**Figure 5:**
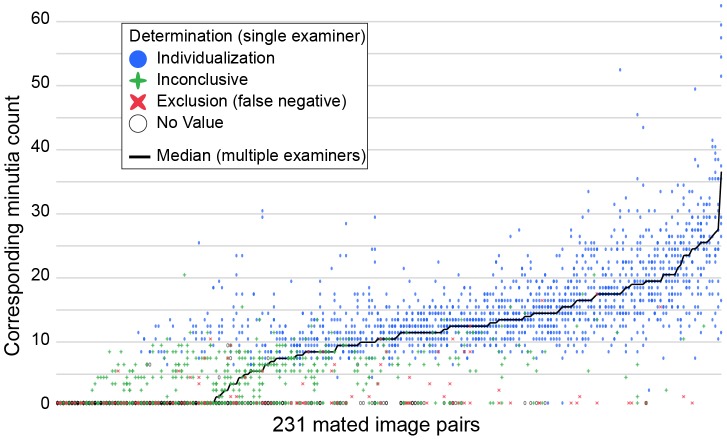
Corresponding minutia count (y-axis) and determination (color) by image pair (x-axis). Each column of points contains the set of all responses for a given image pair. Some points are superimposed, indicated through color blending. X-axis is sorted by median, then by mean corresponding minutia count. Latents that were determined NV and not compared are shown as having zero corresponding minutiae. NV responses with one or more corresponding minutiae are due to examiners changing their value determinations during Comparison. (n = 2796 responses by 165 examiners to 231 mated image pairs.)

**Figure 6:**
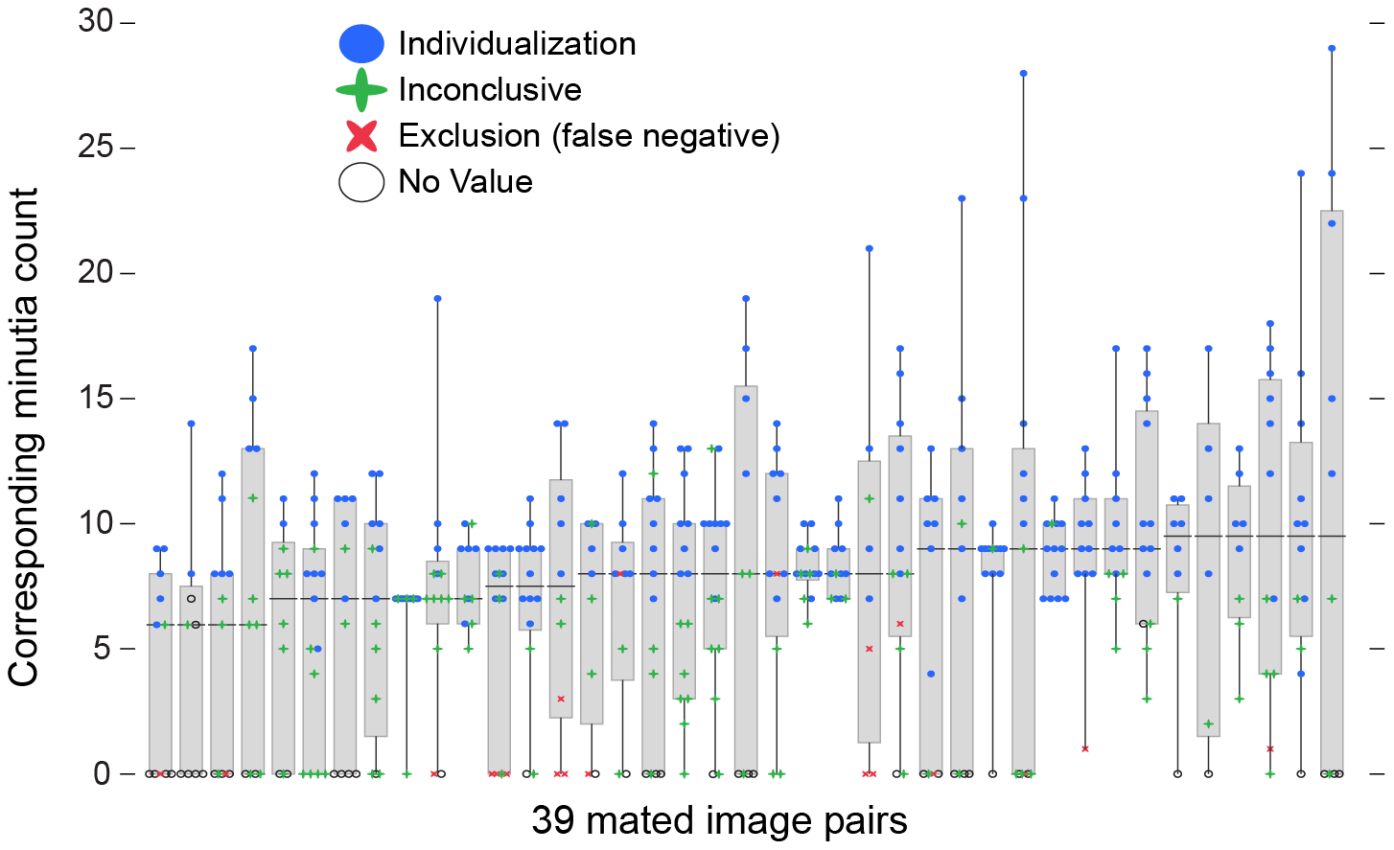
Detail of Figure 5 for the 39 image pairs that had median corresponding minutia counts between 6 and 9.5, with the addition of box plots showing interquartile range, minima, and maxima. (n = 452 responses; 6 to 16 responses per image pair.)

Figure 5 shows the association between corresponding minutia counts and determinations, as well as the reproducibility of counts and determinations among examiners. The strong association between examiners' minutia counts and their own determinations shown in Figure 3B is seen here as a color change in the vertical dimension. Figure 6 shows a subset of this data to more clearly reveal the interexaminer variability on each image pair. For most image pairs (x-axis), we see substantial interexaminer variability in both the corresponding minutia counts (vertical spread) and determinations (color). This extensive variability means that we must treat any individual examiner's minutia counts as interpretations of the (unknowable) information content of the prints: saying “the prints had N corresponding minutiae marked” is not the same as “the prints had N corresponding minutiae.” The variability also implies that one examiner's minutia count is a weak predictor of another examiner's determination: for example, while we might have assumed that having one examiner mark 13 or more corresponding minutiae and individualize would guarantee that any other examiner would also individualize, that is not true; most of the mated image pairs had one or more examiners mark 13 or more corresponding minutiae. Appendix S15 includes additional charts clarifying some of these relations and showing results from the Analysis phase. See Appendix S16 for corresponding data on the nonmated pairs.

In our previous reports [2], [4], we observed that variability in determinations was concentrated on certain image pairs, but did not characterize the attributes of those prints. In Figure 5 and Figure 6, we see that the reproducibility of determinations is associated with the median corresponding minutia count and is lowest on image pairs with a median corresponding minutia count between about six to nine (Appendix S14). Interexaminer variability in corresponding minutia counts is seen across all image pairs, except where there is unanimous agreement on zero corresponding minutiae. Disagreements on sufficiency for individualization tend to be associated with substantial disagreements on corresponding minutiae; similar observations have been made previously [10], [19], [5], [49]. When examiners made an inconclusive determination, they typically reported fewer than 12 corresponding minutiae; these counts were independent of the median count reported by those who individualized. The individual examiners' determinations generally transition from inconclusive to individualization between about six to nine corresponding minutiae, which is relatively independent of the other examiners' counts. An increasing median corresponding minutia count is associated with fewer examiners making inconclusive determinations. The variation in the counts remains even when examiners agree on individualization. However, the critical instances occur when annotation disagreements are associated with differing determinations. Failure to see correspondence is a notable cause for variation in the counts: on 42% of inconclusive determinations on mated pairs, examiners marked no corresponding minutiae. “Corresponding features” is only a particularly meaningful concept when the examiner is at least leaning toward individualization: if the examiner cannot find any areas of possible correspondence or “anchor points”, marking no corresponding points would be the expected response. Individualization disagreements arose on 61% of mated pairs. When an examiner fails to individualize a mated pair that is individualized by another examiner, it is considered in some agencies as a “missed ID”: 10% of responses were missed IDs on mated pairs that were individualized by the majority of examiners.

Differences in minutia counts understate the variability among examiners: annotations may have similar minutia counts but differ greatly in which specific minutiae were marked. Some differences relate to lack of concurrence in what constitutes minutiae, especially within cores and deltas. Some of the variability in minutia selection may be due to the examiners themselves not being consistent in their minutia selection: in this study, a small number of latents were presented to examiners twice, and substantial variability of annotation was observed (see Appendix S13).

An individual examiner's corresponding minutia counts are not highly consistent descriptions of how well the image pairs correspond: given an image pair as a stimulus, the minutia counts are subjective responses with limited reproducibility among examiners. Based on our inspection of the annotated images, we notice several factors that contribute to interexaminer differences in which minutiae were marked. These include whether to mark minutiae that are not clear or are difficult to interpret; what constitutes a minutia close to cores and deltas; the extent of the region of interest, such as when marking discontinuous impressions; and how to mark features such as incipient ridges and dots, which some examiners marked as minutiae.

To quantify the variability in corresponding minutia counts and attribute it to specific sources, we use an Analysis of Variance main effects model with minutia counts as responses to the image pairs and the examiners to whom they were assigned:
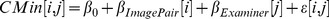
(Eq 3)where the betas are unknown parameters for an intercept, each image pair, and each examiner.

Because of the large numbers of image pair and examiner parameters, they were analyzed as if they were random samples from populations of images pairs and examiners, respectively. This “random effects” model was analyzed using Restricted Maximum Likelihood Estimation (REML). If examiners always agreed on the corresponding minutia count for each image pair, all of the variance would be attributed to image pair effects. We find that 65% of the variance can be attributed to image pair effects, 11% to examiner effects, and 24% is residual (Table 3). These examiner effects represent a tendency by some examiners to mark more minutiae than other examiners. This results in a standard deviation of 2.8 corresponding minutiae, after controlling for image pair effects; this value is large in relation to the critical range of about six to nine corresponding minutiae in which examiner determinations generally transition from inconclusive to individualization (Figure 3B). Some of the residual variance is likely to be associated with limited repeatability of minutia counts by individual examiners (Appendix S13; [18]).

**Table 3:** Image pair and examiner effects on corresponding minutia counts, showing restricted maximum likelihood estimates.

Random Effect	St. Dev.	Variance	(95% bounds)	% of Total Variance
Examiner	2.8	8.1	(6.4–10.5)	11.0%
ImagePair	6.9	47.6	(39.7–58.1)	64.6%
Residual	4.2	18.0	(17.0–19.0)	24.4%

(n = 2796 responses by 165 examiners to 231 mated image pairs)

### Predicting another examiner's individualization determination

From the Black Box study [2], [4], we saw that reproducibility of individualization determinations is much higher on some image pairs than others, but that study did not provide any data for predicting for a given image pair whether agreement would be high or low. The only current method to assess whether an individualization or inconclusive determination is appropriate in a particular case is by consensus among examiners. Therefore, it is of great interest to estimate the probability that one examiner's determination of sufficiency would be reproduced by other examiners, taking into account that examiner's expressed basis for the determination.

We evaluated several logistic regression models predicting individualization determinations by one examiner from the responses (annotation and determination) of another examiner to the same image pair (Table 4). As we saw when modeling associations between annotations and determinations by the same examiner, accounting for factors such as clarity or the examiner's rating of comparison difficulty does not substantially improve upon predictions based on *CMin* alone (see Appendix S10 for additional performance measures).

**Table 4:** Misclassification rates for models using one examiner's annotations and determinations to predict a second examiner's individualization determinations.

Predictors		Misclass.
**None (base rate)**		39.8%
**Difficulty**		26.3%
**OverallClarity**		23.7%
**OverallClarity; CMin**		20.9%
**Determination**	*{Individualization, Insufficient}*	20.5%
**CMin**		20.4%
**CMin_green; CMin_yellow**		20.0%
**Determination; CMin**		20.0%
**CMin; Difficulty**		19.9%

(14,608 paired responses by 165 examiners, reweighted to n = 231 mated image pairs) See Table 2 for definitions of predictor variables.

Comparing the paired-examiner models of Table 4 with the same-examiner models of Table 2 shows that although examiners' associations and determinations are strongly associated, these same annotations are not as strongly associated with other examiners' determinations; for example, the misclassification rate for paired-examiner models based on corresponding minutia count is 20.4% versus 6.0% same-examiner models. The reason for this difference is the substantial interexaminer variability in both corresponding minutia counts and determinations, both of which negatively affect this prediction. If annotations from multiple examiners are available (not typical in operations), we can predict determinations using voted metrics for each image pair, such as median *CMin*, which are less affected by the interexaminer variability in corresponding minutia count.

**Figure 7:**
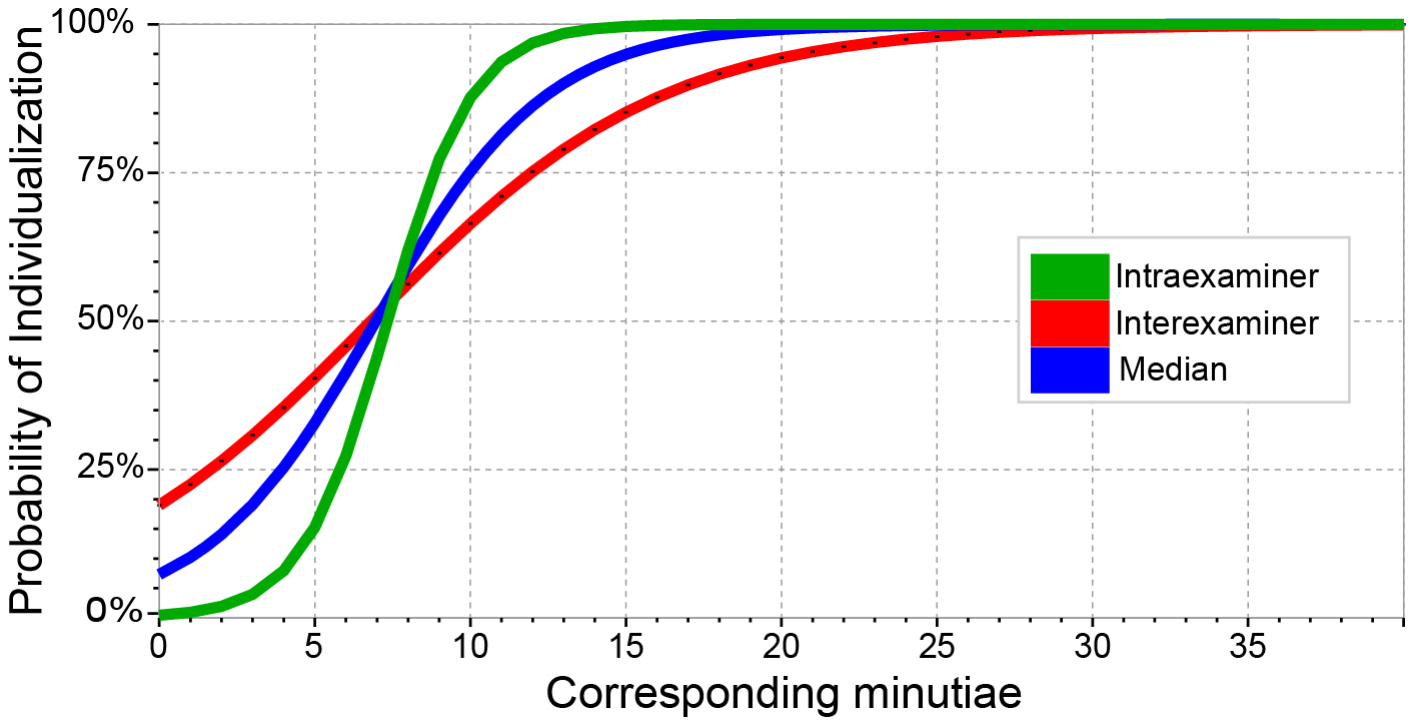
Logistic models estimating the probability of individualization based on corresponding minutia counts, on mated image pairs: (green) probability that an examiner would individualize based on the ***same*** examiner's corresponding minutia counts (6.0% misclassification, see Table 2); (red) probability that ***another*** examiner would individualize based on this examiner's minutia counts (20.4% misclassification, Table 4); (blue) probability that an examiner would individualize based on the ***median*** of all examiners' corresponding minutia counts (13.6% misclassification, Table 5).

Figure 7 shows the substantial differences in predictive ability among the same-examiner *CMin* model, the paired-examiner *CMin* model, and a model based on the median(*CMin*) across multiple examiners. All three models estimate approximately 50% probability of individualization at seven corresponding minutiae. However, the models differ on where they estimate 90% probability of individualization: when the ***same*** examiner marked 10 corresponding minutiae (green), when the ***median*** count was 13 (blue, median), or when ***another*** examiner marked 17 (red). Examiners' determinations are much more closely aligned with their own *CMin* than with others' *CMin*, limiting the effectiveness of using one examiner's annotations to predict other examiners' determinations.

### Factors explaining agreement on sufficiency

Whether a given image pair would be individualized by an examiner can be seen as a function of that examiner's tendency to make individualization determinations and the tendency of all examiners to individualize that image pair. By modeling examiner determinations as dependent responses to which image pair was presented to which examiner, we can establish how much of the observed variation in examiner responses is associated with these two factors and the extent to which these two factors fall short of a full explanation. Letting *π[i,j]  =  Probability(Individualization)[i,j]*, for image pair *i* and examiner *j*, we can fit a logistic regression model such as
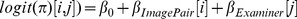
(Eq 4)which has separate parameters for each image pair and each examiner (394 degrees of freedom). The relative contributions of examiner effects and image pair effects are summarized in Table 5A. Predicting individualizations based on which image pair was compared reduces misclassification from a base rate of 38.1% to 13.0% (Table 5A, *ImagePair*); this is equivalent to predicting the determination for an image pair based on majority vote (13% of determinations were in the minority). This 13.0% misclassification rate defines a limit for any model of this data based only on image attributes, as a necessary consequence of examiner disagreements on the determinations; if examiners were always unanimous on their individualization determinations, the misclassification rate for the *ImagePair* model would be zero. The *Examiner* model (32.8%) reduces misclassification from the base rate due to differences among examiners' individualization rates.

**Table 5:** Misclassification rates for models describing individualization as a dependent response to (A) image pairs and examiners and (B) attributes of the image pairs as estimated by median statistics (derived from all examiner responses).

	Predictors	DF	Misclass
	None (base rate)	0	38.1%
**A**	Examiner	164	32.8%
	ImagePair	230	13.0%
	ImagePair; Examiner	394	6.3%
**B**	CD_rate	1	31.6%
	MedianOverallClarity	1	24.3%
	CD_rate; MedianOverallClarity	2	23.1%
	Median(CMin); MedianOverallClarity	2	13.6%
	Median(CMin)	1	13.6%
	Median(CMin); Examiner	165	9.5%

n = 2671 responses. CD_rate: proportion of examiners who marked a core or delta. MedianOverallClarity: Overall Clarity from the median corresponding clarity map. DF  =  degrees of freedom. See Appendix S12 for additional models and performance measures.

Having thus evaluated the overall magnitude of the image effects, we then fit simple models based on specific measures derived from the annotations (Table 5B). By comparing the models of Table 5B with those of Table 5A, we can assess how well those simple models explain the basis for sufficiency decisions. Note that the models describe image pairs using predictors that are fixed for each image pair (indexed by *[i]*, not by *[i,j]*) in order to model the effects of the image pairs on determinations. For this purpose, we use voted metrics derived from the annotations of multiple examiners to produce our best estimate of each attribute. The 13.6% misclassification of the *Median(CMin)* model is nearly as low as the rate for the *ImagePair* model (13.0%), and therefore accounts for nearly all of the observed variation in the examiner responses that could be explained by attributes derived from the image pair; paired-examiner models accounting for attributes such as clarity, complexity, or nonminutia features ***cannot*** introduce much additional predictive information, as they are bounded by the 13.0% misclassification due to the reproducibility of determinations. Just as we saw for same-examiner predictions, corresponding minutia count is the dominant factor in determinations.

When we model individualization determinations as responses to both *Median(CMin)* and *Examiner*, the misclassification rate drops to 9.5% (vs. 13.6% for *Median(CMin)* alone); much of the further reduction to 6.3% in the *ImagePair + Examiner* model may be due to overfitting. We know from our previous research that a substantial proportion of determinations are not repeated on retest [4], and we estimate that more than half of the 9.5% misclassification rate can be attributed to this lack of repeatability (Appendix S17). The remainder of the misclassification is due to ImagePair*Examiner interaction effects.

Comparing the models in Table 5 with those in Table 2 reveals that examiners' determinations are much more strongly associated with their own corresponding minutia counts than with the median estimates, as we saw in Figure 7. This implies that individual annotations are a good description of the basis for examiner determinations, as opposed to suggesting that examiners all tend to see and rely upon the same features, yet describe them inconsistently. The limited reproducibility of corresponding minutia counts demonstrates that the subjective annotations of these examiners do not consistently describe intrinsic attributes of the images themselves. By comparing the *ImagePair* model (misclassification rate 13.0%, Table 5) to the same-examiner *CMin* model (misclassification rate 6.0%, Table 2), we see that the individual examiner's minutia counts are part of a combined response to the images that reflects the subjective outcome of the ACE process and goes beyond the consensus response to the images reflected in the *ImagePair* model.

### Effect of point standard

Ten of the participants who indicated in the questionnaire that their agency or country has a 12-point standard conformed to that standard in their responses (see discussion in Appendix S18). Although one might expect that a high point count threshold would be associated with a lower individualization rate, participants following a 12-point standard were no less likely to individualize than those without a point standard. The individualization rate was 69% among those examiners following a 12-point standard (n = 10) and 62% among the remainder (n = 155); the difference is not statistically significant.

**Figure 8:**
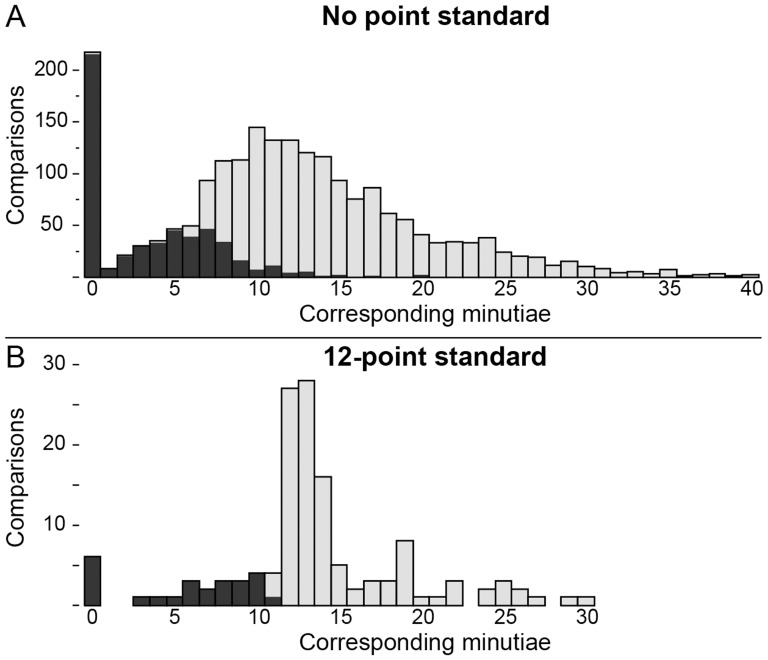
Distribution of corresponding minutia counts by (A) the majority of participants (n = 2062 comparisons of mated pairs by 155 examiners) and (B) those participants following a 12-point standard (n = 135 comparisons of mated pairs by 10 examiners). Colored by determination: inconclusive (black), individualization (gray); NV not included.

As shown in Figure 8, the number of corresponding minutiae examiners marked differed greatly between those following a 12-point standard and the remainder of participants. Given the balanced assignments, we would expect no substantial difference in these two distributions: we would expect a smooth distribution in the number of corresponding minutiae that examiners marked based on how the prints were selected. Instead we see abrupt steps in both distributions: those examiners following a 12-point standard were much more likely to mark 12 corresponding minutiae than 11, and those without a point standard were much more likely to mark seven corresponding minutiae than six. Evett and Williams [10] made a similar observation, noting that examiners following a 16-point standard avoided counting 15 points. These abrupt steps indicate that examiners' counting appears to be influenced by their determinations. Conceptually ACE separates examination into different phases, so that corresponding features are defined in Comparison prior to the determination being made in Evaluation. However, these results indicate that we cannot assume causality between minutia counts and determinations. We might hypothesize that examiners subconsciously reach a preliminary determination quickly and this influences their behavior during Comparison (e.g., level of effort expended, how to treat ambiguous features). Additional supporting data from the Analysis phase is presented in Appendix S18. The sample of participants following a 12-point standard is very small and not necessarily broadly representative of examiners who follow point standards.

### Minutia thresholds

We have seen that across multiple examiners there is a gradual transition from inconclusive to individualization that can be described in terms of minutia counts. We might expect individual examiners to each have their own thresholds, and that these would vary from examiner to examiner with the consequence that some examiners individualize more often than others. The minimum number of corresponding minutiae that each examiner reported when individualizing varied among examiners. More than one-third of examiners individualized with eight or fewer minutiae, but others had a minimum count as high as 14. While some examiners based individualizations on fewer than seven minutiae, on review, all of the outliers with fewer than five corresponding minutiae can be explained as improper annotation, and most of the outliers with five or six corresponding minutiae rely on nonminutia features (Appendix S9). After discounting the outliers that we believe were due to improper annotation, we did find examples of individualizations with as few as six corresponding minutiae, or five minutiae and two level-3 features.

We investigated the reasons for this wide variation in the data to determine whether the minimum minutia counts are indicative of thresholds that differ among examiners, or are artifacts of sample size or data selection. In order to understand the substantial dispersion in minimum minutia count, we performed three simulations to isolate contributing factors: random variations due to small sample sizes, variations associated with differing individualization rates among examiners, and variations associated with differences in marking minutiae among examiners. These simulations are presented in Appendix S19. Our simulations demonstrate that most of the dispersion in minimum minutia count is a consequence of the limited number of measurements obtained per examiner (i.e., small sample size: 17 mated comparisons per examiner). The minimum is an extreme statistic and biased upwards: if each examiner had been assigned many more comparisons, more opportunities would have lowered the observed minimum for many examiners. In particular, on a larger test, we would expect the proportion of examiners who individualized with seven or eight corresponding minutiae to increase.

The small samples do not account for all of the variation in minimum minutia counts. As we showed above (Table 5A, *Examiner*), there are real differences among examiners' individualization rates, more than can be explained by the random test assignments. Our simulations demonstrate that these differences in individualization rates contribute very little to the dispersion in minimum minutia count. Nevertheless, we do observe some differences among examiners in the minimum number of corresponding minutiae marked when individualizing (beyond imprecision and chance): notably, some examiners only individualize when they mark nine or more corresponding minutiae. The simulations show that, apart from sampling limitations, the primary significance of a higher minutia count threshold appears to relate to differences in examiner judgment as to which features to mark (i.e., a higher minimum count means some examiners mark more minutiae than others on the same prints), not to differences in judgment as to which prints to individualize (i.e., a higher minimum count does not mean that they are less likely to individualize). Differences in individual minimum minutia count thresholds do not appear to be an important factor contributing to differing individualization rates.

## Conclusions

In a controlled study designed to ascertain the factors that explain examiners' determinations of sufficiency for individualization, latent print examiners recorded the bases for their determinations by providing detailed, standardized annotations of the fingerprints. The fingerprints used in this study were selected to test the boundaries of sufficiency for individualization determinations, and we deliberately limited the proportion of image pairs on which we expected examiners to have unanimous decisions; therefore, the reproducibility and error rates reported in this study should not be assumed to represent latent print examination in general.

While erroneous individualizations and exclusions are obvious concerns, differences in examiners' assessments of sufficiency also have serious operational implications. Such differences may result in conflict between examiners at the time of verification or in court, and in the failure to identify a criminal subject, which could result in that individual committing additional crimes. Disagreements among examiners on whether there is sufficient information to make an individualization does not imply that the determinations are erroneous (i.e., false positives or false negatives); for a discussion of error rates, we direct the reader's attention to our previous Black Box study [2].

The study was designed to assess the associations between annotations and determinations, not to assess whether examiners' decisions to to make individualization vs. inconclusive determinations were “correct” in an absolute sense. From our previous work, we expected variability among examiners with respect to individualization determinations: we reported in [4] that two examiners agreed whether or not to individualize 86.6% of the time; in other words, 13.4% of the time a second examiner in that study would disagree whether the information content was sufficient to make an individualization decision. Disagreements on borderline decisions are expected, and requiring categorical decisions exaggerates examiner differences. Two examiners may both agree that a given decision is borderline, but reach different determinations in part because the discrete categories force them to make a choice.

The study revealed substantial differences among examiners' annotations. We cannot tell whether this is due to differences in how examiners see and interpret the data or merely to differences in how they document their interpretations. Differences in interpretation may arise at several points during examination: an examiner analyzing an unclear print must decide whether there is sufficient continuity when determining the limits of the region of interest to be used; an examiner analyzing a ridge within an unclear region must determine whether or not features are present; and an examiner must decide during Comparison whether potentially corresponding features are within a reasonable tolerance for differences in appearance. Each of these decisions may contribute to differences in interpretations and thus to differences in annotations. Additionally, there were many cases in which examiners made inconclusive determinations on mated pairs because those examiners failed to find any correspondences between the prints.

In addition to differences in interpretation, a lack of clear criteria in the latent print discipline specifying when and how to mark features may have contributed to much of the observed variability in annotations [50], [20], [49], [51]. The lack of generally accepted and detailed standards for defining and recording the bases for conclusions limits the effectiveness of studies such as this, as well as the effectiveness of reviews of operational casework. Courts are now more frequently requiring that examiners demonstrate their bases for conclusions (during discovery, admissibility, and trial). Examiners are rarely trained specifically on how to interpret, select, and record features (other than for AFIS searches) in a standard, reproducible manner. Consistently applied and rigorously defined methods of performing and documenting ACE-V would result in a more transparent process, which could be more readily validated in research or in operations. Standardized annotation, such as the ANSI/NIST-ITL markup used here, may be of operational benefit as a means of documenting and communicating the bases for examiners' determinations, especially for complex or disputed prints. Although the annotations collected in this study were based on recent standards, we recognize that the software and instructions were unfamiliar to many participants, and this may have contributed to the variability in annotations.

We found examiners' individualization determinations to be closely related to the number of corresponding minutiae marked. Other factors describing the fingerprints, such as clarity and level-3 details, were not as strongly associated, and only a small proportion of the variability in determinations remains unexplained by corresponding minutia count. This finding is consistent with our previous results regarding value determinations [21], and is largely but not entirely consistent with the findings of Neumann et al. [49], who concluded that “sufficiency is mainly driven by the number and spatial relationships between the minutiae observed on the latent and control prints.”

We designed our experiment to allow us to measure the extent to which various factors played a role in determining sufficiency for individualization, following the publication by SWGFAST of a conceptual Sufficiency Graph that depicts a complementary role between quality (an assessment of the overall clarity of the impression) and the quantity of minutiae for sufficiency for individualization [13]. We found, contrary to the SWGFAST proposition, that models accounting for clarity and minutia count performed no better than models that only accounted for minutiae count: we assume clarity influences which minutiae are marked rather than providing additional complementary information.

ACE distinguishes between the Comparison phase (assessment of features) and Evaluation phase (determination), implying that determinations are based on the assessment of features. However, our results suggest that this is not a simple causal relation: examiners' markups are also influenced by their determinations. How this reverse influence occurs is not obvious. Examiners may subconsciously reach a preliminary determination quickly and this influences their behavior during Comparison (e.g., level of effort expended, how to treat ambiguous features). After making a decision, examiners may then revise their annotations to help document that decision, and examiners may be more motivated to provide thorough and careful markup in support of individualizations than other determinations. As evidence in support of our conjecture, we note in particular the distributions of minutia counts, which show a step increase associated with decision thresholds: this step occurred at about seven minutiae for most examiners, but at 12 for those examiners following a 12-point standard. An interesting question for future research is to what extent examiners' latent value and comparison determinations may influence their use (and markup) of minutia and other features.

Although we expected variability in minutia counts, we did not expect the counts to vary as much as they did, especially in those critical cases in which examiners do not agree on their determinations and precise counting might be pivotal. The differences in minutia count understate the variability because the annotations not only differ substantially in total minutia counts, but also in which specific minutiae were selected. The limited reproducibility of minutia markup may be expected to have an operational effect on AFIS latent print searches, which are predominantly based on examiners' markup of minutiae; variability of annotations among examiners implies that search results would vary among examiners. Similarly, proposed models for probabilistic conclusions (e.g., [30], [25], [31]) based on examiners' minutia markup would result in different probability estimates for different examiners or even for the same examiner on different occasions.

Examiners' annotations are much more strongly associated with their own determinations than with those of other examiners. Neumann et al. observed the same result, noting that examiners are internally coherent, but consistency among examiners is low [49]. The observation that different determinations are often associated with substantially different annotations suggests that disagreements over sufficiency arise not only from differences in judgment about what constitutes sufficiency, but also from basic differences in interpretation of the prints.

Whereas our previous Black Box study design [2], [4] was well-suited to estimating overall rates for errors and the reproducibility of determinations, one anticipated benefit of the white box approach used here was that the markups would reveal which determinations would be likely to result in disagreements related to the marginal sufficiency of the information. For quality assurance, it would be operationally desirable to flag sufficiency decisions that may be unreliable so that extra action could be taken: for example, flagging determinations that may not be highly reproducible, or flagging instances in which an examiner's determinations do not follow from that examiner's own markup. However, because of the limited reproducibility of minutia counts and determinations, one examiner's annotation and determination are often unreliable predictors of another examiner's determination. More consistency in annotations, which could be achieved through standardization and training, should lead to process improvements and provide greater transparency in casework.

## Supporting Information

Appendix S1 **Glossary.**
(PDF)

Appendix S2 **Test procedure.**
(PDF)

Appendix S3 **Fingerprint data.**
(PDF)

Appendix S4 **Post-processing of response data.**
(PDF)

Appendix S5 **Summary of examiner determinations.**
(PDF)

Appendix S6 **Timing.**
(PDF)

Appendix S7 **Participant background survey responses.**
(PDF)

Appendix S8 **Associations between examiners' annotations and their determinations.**
(PDF)

Appendix S9 **Low count individualizations.**
(PDF)

Appendix S10 **Reproducibility of individualization determinations.**
(PDF)

Appendix S11 **Models of latent value sufficiency.**
(PDF)

Appendix S12 **Models of sufficiency for individualization.**
(PDF)

Appendix S13 **Repeatability of minutia counts (Analysis phase).**
(PDF)

Appendix S14 **Reproducibility of determinations by median corresponding minutia count.**
(PDF)

Appendix S15 **Reproducibility of responses.**
(PDF)

Appendix S16 **Corresponding minutia counts and determinations for nonmated image pairs.**
(PDF)

Appendix S17 **Estimating repeatability of determinations.**
(PDF)

Appendix S18 **Effect of point standard.**
(PDF)

Appendix S19 **Minimum number of corresponding minutiae.**
(PDF)

Appendix S20 **White Box Latent Examiner Study — Instructions.**
(PDF)

Information S1 **Single-file concatenation of Appendices S1 through S20.**
(PDF)

Video S1 **White Box Latent Print Examiner Study Tutorial (Video).**
(MP4)
