# Correction: Measuring What Latent Fingerprint Examiners Consider Sufficient Information for Individualization Determinations

**DOI:** 10.1371/journal.pone.0118172

**Published:** 2015-02-06

**Authors:** The PLOS ONE Staff

Fig. 3 is incorrect. The image for Figure 8 was inadvertently published twice. The authors have provided a corrected version of Fig. 3 here.

**Fig 3:**
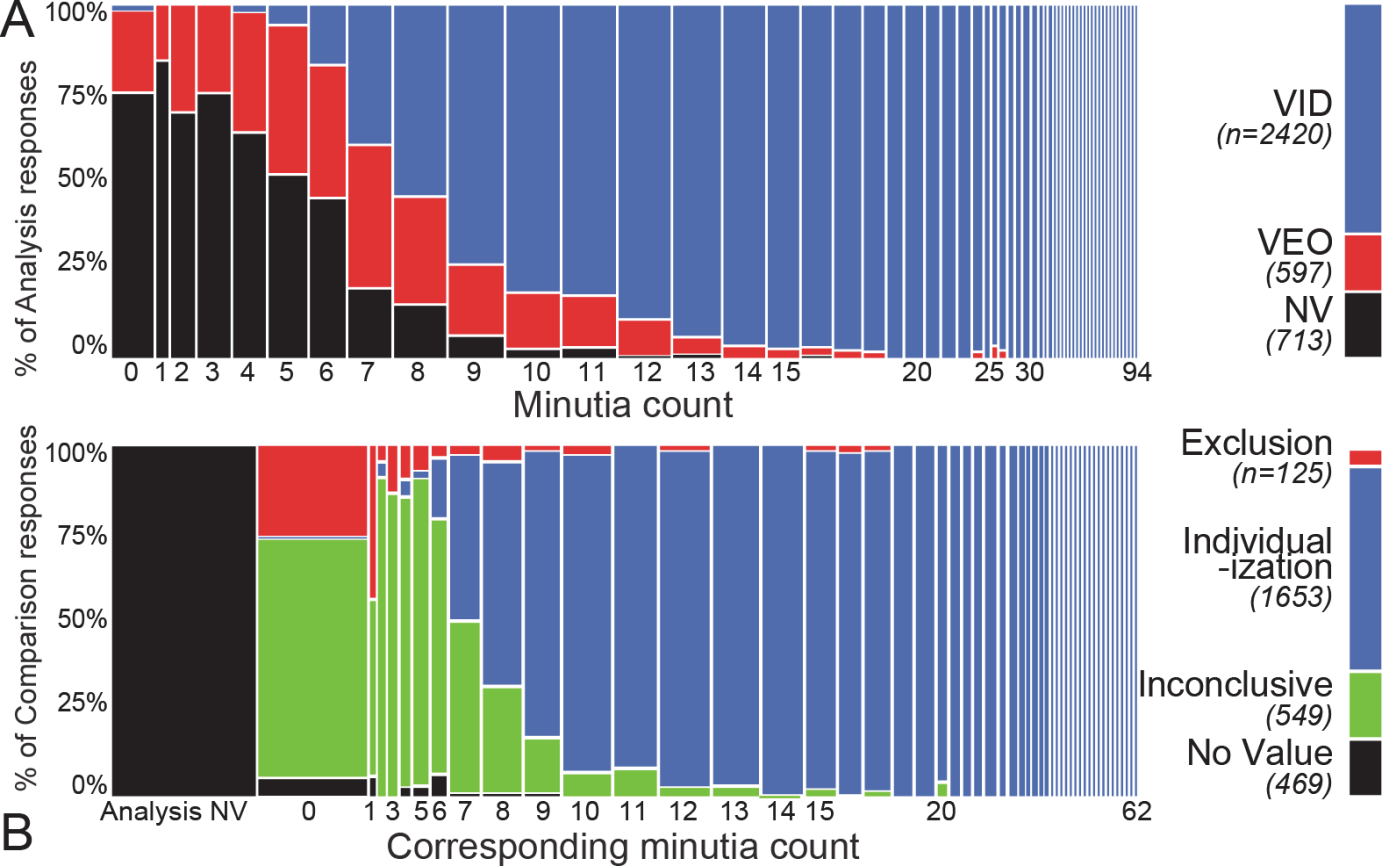
Associations of (A) minutia count and value determinations from analysis of the latent (n = 3730); (B) corresponding minutia count and determinations from comparison of latent and exemplar prints on mated data (n = 2796). In (B), 1.6% of determinations with 12 or more corresponding minutiae marked were not individualized. A few responses in (B) indicate NV with corresponding minutiae due to examiners changing their value determinations during Comparison.

S1 Video is truncated. Please view the correct S1 Video here.

## Supporting Information

S1 Video White Box Latent Print Examiner Study Tutorial (Video).
