# Supplementary material for: Measuring What Latent Fingerprint Examiners Consider Sufficient Information for Individualization Determinations
Source: PLoS One. 2014 Nov 5;9(11):e110179. doi: 10.1371/journal.pone.0110179 (PMC4221158; doi:10.1371/journal.pone.0110179)
Supplement: Appendix S1 — Glossary. (PDF) [file pone.0110179.s001.pdf]

Appendix SI-1 Glossary

This section defines terms and acronyms as they are used in this paper.

**ACE**	The phases of ACE-V prior to verification: Analysis, Comparison, Evaluation.
**ACE-V**	The prevailing method for latent print examination: Analysis, Comparison, Evaluation, Verification.
**AFIS**	Automated Fingerprint Identification System (generic term)
**Analysis phase**	The first phase of the ACE-V method. In this test, the examiner annotated the latent and made a value determination before seeing the exemplar print. In this report, we capitalize Analysis when referring to the ACE phase.
**ANSI/NIST-ITL**	An electronic file and interchange format that is the basis for biometric and forensic standards used around the world, including the FBI's EBTS and Interpol's INT-I, among others. As of 2011, this incorporates the Extended Feature Set (EFS) definition of friction ridge features used in this study [1].
**Clarity**	The clarity of a friction ridge impression refers to the fidelity with which anatomical details are represented in a 2D impression, and directly corresponds to an examiner's confidence that the presence, absence, and details of the anatomical friction ridge features in that area can be correctly discerned in that impression. (Note: The term "clarity" is used here instead of "quality" to avoid ambiguity, since the latter term as used in biometrics and forensic science is often used to include not only clarity but also the quantity or distinctiveness of features.)
**Comparison/Evaluation phase**	The second and third phases of the ACE-V method. In this test, there was no procedural demarcation between the Comparison and Evaluation phases of the ACE-V method; hence, this refers to the single combined phase during which both images were presented side-by-side. For brevity, in this report we use "Comparison" to refer to the Comparison/Evaluation phase.
**Comparison determination**	The determination of individualization, exclusion, or inconclusive reached in the Comparison/Evaluation phase of the test. SWGFAST [2] refers to this determination as the Evaluation Conclusion.
**Corresponding clarity map**	The corresponding clarity map represents the minimum clarity at each location in the aligned latent and exemplar clarity maps, as described in [3]. These maps were constructed from the examiners' annotations by post-processing software whenever at least three corresponding features were marked by the examiner. A thin-plate spline algorithm was used to align the latent and exemplar prints. (See local clarity map)
**Corresponding features**	A 1:1 relationship between a feature in a latent and a feature in the exemplar in which the feature is present in both images.
**Debatable correspondence**	A relationship between a feature in a latent and a feature in the exemplar in which there is an apparent correspondence between a feature in the latent and a feature in the exemplar that does not rise to the threshold of definite correspondence. (Not to be confused with debatable ridge flow or debatable features, which were indicated by painting the image clarity.)
**Determination**	An examiner's decision: the Analysis phase results in a Value determination, and the Comparison/Evaluation phase results in a Comparison determination.
**Exclusion**	The comparison determination that the latent and exemplar fingerprints did not come from the same finger. For our purposes, this is *exclusion of source*, which means the two impressions originated from different sources of friction ridge skin, but the subject cannot be excluded, whereas *exclusion of subject* means the two impressions originated from different subjects.
**Exemplar**	A fingerprint from a known source, intentionally recorded.
**Extended Feature Set**	The definition of friction ridge features incorporated into the ANSI/NIST-ITL standard.
**False negative**	An erroneous exclusion of a mated image pair by an examiner.
**False positive**	An erroneous individualization of a nonmated image pair by an examiner.
**Feature**	Minutia, core, delta, or "other" point marked by examiners. In this study, a feature has a location (x,y coordinate) but no direction.
**IAFIS**	The FBI's Integrated Automated Fingerprint Identification System (as of 2013, IAFIS latent print services have been replaced by the FBI's Next Generation Identification (NGI) system).
**IAI**	International Association for Identification
**Image**	A fingerprint as presented on the computer screen to test participants. The test software permitted rotating, panning, zooming, tonal inversion, and grayscale adjustment of the image.
**Incipient ridge**	A friction ridge not fully formed that may appear shorter and thinner in appearance than fully developed friction ridges.
**Inconclusive**	The comparison determination that neither individualization nor exclusion is possible.
**Individualization**	The comparison determination that the latent and exemplar fingerprints originated from the same source. Individualization is synonymous with identification for latent print determinations in the U.S. Both are defined as: "the decision by an examiner that there are sufficient discrimination friction ridge features in agreement to conclude that two areas of friction ridge impressions originated from the same source. Individualization of an impression to one source is the decision that the likelihood the impression was made by another (different) source is so remote that it is considered as a practical impossibility." [2,4]
**Insufficient**	When referring to examiner determinations (response data), "Insufficient" responses include both no value and inconclusive determinations.
**Latent (or latent print)**	A friction ridge impression from an unknown source. In North America, "print" is used to refer generically to known or unknown impressions [5]. Outside of North America, an impression from an unknown source (latent) is

	often described as a "mark" or "trace," and "print" is used to refer only to known impressions (exemplars).
**Level-3 detail**	Friction ridge dimensional attributes such as width, edge shapes, and pores.
**Local clarity map**	A color-coded annotation of a friction ridge image indicating the clarity for every location in the print, as described in [3] and defined in the ANSI/NIST-ITL standard [1].
**Mated**	A pair of images (latent and exemplar) known *a priori* to derive from impressions of the same source (finger). Compare with "individualization," which is an examiner's ***determination*** that the prints are from the same source.
**Median clarity map**	A local clarity map combining the annotations from multiple examiners, based on the median clarity at each location across the clarity maps from all examiners who annotated the clarity of an image (or image pair, for median corresponding clarity maps).
**Minutiae**	Events along the path of a single path, including bifurcations and ending ridges. In this study, examiners did not differentiate between bifurcations and ending ridges. Dots are considered minutiae in some uses, but not for AFIS usage; in this study, examiners were instructed to mark dots as "other" features.
**Misclassification rate**	The proportion of responses that would be incorrectly classified as individualization or not individualization for a given model.
**Missed ID**	Failure by an examiner to individualize a mated pair that was individualized by any other examiners (also known as a "missed individualization" or "missed identification").
**Noncorresponding feature**	A discrepancy – a feature that exists in one print and is definitely not present in the other print. Participants were instructed to indicate points in one print that definitely do not exist in the other print as needed to support an exclusion determination.
**Nonmated**	A pair of images (latent and exemplar) known *a priori* to derive from impressions of different sources (different fingers and/or different subjects).
**NV (No value)**	The impression is not of value for individualization and contains no usable friction ridge information. See also VEO and VID.
**Other point**	In this study, features such as scars, dots, incipient ridges, creases and linear discontinuities, ridge edge features, or pores (i.e., features other than minutiae, cores, and deltas).
**Overall Clarity**	A metric based on the size and consistency of the areas of the various levels of clarity in a local clarity map, described in [3]. Overall Clarity ranges from 0-100 and was developed to correspond to human examiner assessments of the value and difficulty of an image.
**Qualified examiner**	Determined by an agency to be appropriately qualified as a latent print examiner. Used instead of "certified" in some organizations to differentiate from the IAI certification, "Certified Latent Print Examiner."
**Repeatability**	Intraexaminer agreement: when one examiner provides the same response (annotation or determination) to a stimulus (image or image pair) on multiple occasions.
**Reproducibility**	Interexaminer agreement: when multiple examiners provide the same response (annotation or determination) to a stimulus (image or image pair).
**Source**	An area of friction ridge skin from which an impression is left. Two impressions are said to be from the "same source" when they have in common a region of overlapping friction ridge skin.
**Sufficient**	An examiner's assessment that the quality and quantity of information in a print (or image pair) justifies a specific determination (especially used with respect to individualization).
**SWGFAST**	Scientific Working Group on Friction Ridge Analysis, Study and Technology
**ULW**	The FBI's Universal Latent Workstation software [6].
**Unassociated feature**	A feature marked in one print for which the examiner did not indicate any level of correspondence or non-correspondence with respect to the other print (often either obscured or outside the corresponding area).
**Value determination**	An examiner's determination of the suitability of an impression for comparison: value for individualization (VID), value for exclusion only (VEO), or no value (NV). A latent value determination is made during the Analysis phase. Agency policy often reduces the three value categories into two, either by combining VID and VEO into a value for comparison (VCMP) category or by combining VEO with NV into a "not of value for individualization" (Not VID) category [survey in 7].
**VCMP**	Value determination based on the analysis of a latent that the impression is of value for comparison (either VEO or VID).
**VEO**	Value determination based on the analysis of a latent that the impression is of value for exclusion only and contains some friction ridge information that may be appropriate for exclusion if an appropriate exemplar is available. See also NV and VID.
**Verification**	The final phase of ACE-V: the independent application of the ACE process by a subsequent examiner to either support or refute the conclusions of the original examiner. Not addressed in this study.
**VID**	Determination based on the analysis of a latent that the impression is of value and is appropriate for potential individualization if an appropriate exemplar is available. See also VEO and NV.

1 National Institute of Standards (2011) American National Standard for Information Systems: Data format for the interchange of fingerprint, facial & other biometric information. ANSI/NIST-ITL 1-2011. (http://fingerprint.nist.gov/standard)

2 SWGFAST (2013) Standards for Examining Friction Ridge Impressions and Resulting Conclusions, Version 2.0. (http://www.swgfast.org/documents/examinations-conclusions/130427_Examinations-Conclusions_2.0.pdf)

3 Hicklin RA, Buscaglia J, Roberts MA (2013) Assessing the clarity of friction ridge impressions. *Forensic Sci Int* **226(1)**:106-117.

4 SWGFAST (2012) Individualization / Identification Position Statement, Version 1.0. (http://swgfast.org/Comments-Positions/120306_Individualization-Identification.pdf)

5 SWGFAST (2011) Standard terminology of friction ridge examination, Version 3.0. (http://swgfast.org/documents/terminology/110323_Standard-Terminology_3.0.pdf)

6 Federal Bureau of Investigation; Universal Latent Workstation (ULW) Software. (https://www.fbibiospecs.org/Latent/LatentPrintServices.aspx)

7 Ulery BT, Hicklin RA, Buscaglia J, Roberts MA (2011) Accuracy and reliability of forensic latent fingerprint decisions. Proc Natl Acad Sci USA **108(19)**: 7733-7738. (http://www.pnas.org/content/108/19/7733.full.pdf)
