# Supplementary material for: Measuring What Latent Fingerprint Examiners Consider Sufficient Information for Individualization Determinations
Source: PLoS One. 2014 Nov 5;9(11):e110179. doi: 10.1371/journal.pone.0110179 (PMC4221158; doi:10.1371/journal.pone.0110179)
Supplement: Appendix S2 — Test procedure. (PDF) [file pone.0110179.s002.pdf]

Appendix SI-2 Test procedure

The tests were administered using a customized version of the FBI's Universal Latent Workstation (ULW) Comparison Tool, distributed on DVDs [1]. Participants were required to sign and return an informed consent form approved by the FBI's Institutional Review Board before receiving the disks. The fingerprint files were encrypted to limit reuse; all participants agreed to return or destroy the DVDs, and uninstall the data and software from all computers. Each participant was assigned a unique identifier, which was associated with a randomized assignment of image comparisons and was used to anonymize results. Test instructions were provided in paper and digital formats (Appendix SI-20) and as an introductory video (Supporting Information S21). Examiners outside the U.S. were advised that a moderate level of English fluency was necessary to understand the test instructions. A set of practice data was included so that participants could become familiar with the software and procedures before starting the test. After completing all examinations, the examiners were required to complete a survey (Appendix SI-7) before submitting test responses. When each participant completed the test, the test software packaged the response data as encrypted files with the fingerprint images removed to limit file size for transmission.

The differences between the White Box software and the ULW Comparison Tool were generally to enable test administration (e.g., handling encrypted data files, presenting specific image pairs to each participant, and submission of results); the White Box software did not include ULW's "ghost cursor" tool, which assists examiners in locating correspondences.

The White Box software included tools for annotation of features, correspondence of features, and ridge clarity. One tool permitted drawing of temporary lines to assist with ridge counting and following ridges; these were not used in analysis. A minimal set of tools was provided for viewing the images (zoom, rotation, scrollbars, tonal reversal, and grayscale controls); more sophisticated tools were not included to limit the effect of differences among examiners regarding expertise with software tools. Additional controls were provided to show or hide the annotation.

Participants were instructed that the computer screen must accurately display the images presented by the software, and the software included a check for that purpose. They were instructed that it was imperative that they conduct the examinations in this study with the same diligence that they would use in performing casework. The test environment was not controlled. Participants were given several weeks to complete the test, but there were no specific time requirements. They were not told what proportion of the image pairs were mated, nor was any feedback on the correctness of responses provided during the test. Participants were able to review and revise their responses before proceeding to the next comparison, revisit previous comparisons, and skip analyses and comparisons and return to them later. Once an examiner indicated that the Analysis phase was complete and proceeded to comparison, the Analysis phase markup and determination were recorded; any subsequent revisions were recorded as part of the Comparison/Evaluation phase. Examiners were able to leave comments associated with specific test responses. Based on these comments, results of one examiner were excluded from analysis (see Appendix SI-5); no other irregularities were mentioned that required excluding data or special handling.

Alpha and beta test releases were used to solidify the software, instructions, and process. The test was distributed to participants in tiered releases to minimize project risks.

Results are anonymous. A coding system was used to ensure anonymity so that even the analysis team could not associate test responses to individual participants. Because of this, there is no way to cross-reference any participants who may have taken both the Black Box and White Box studies.

The white box software enforced the following errors and warnings, which were shown as dialog boxes by the software when the examiner attempted to complete Analysis or Comparison. Errors prevented the examiner from concluding Analysis or Comparison; for warnings the examiner had to click "OK but could be ignored.

Errors and warnings in Latent Analysis:

- Error: Latent Value determination has not been selected
- Error: Latent Quality is not painted
- Warning: Latent is Of Value for ID but fewer than 6 points are marked

Errors and warnings in Comparison:

- Error: Exemplar Value determination has not been selected
- Error: Exclusion determination and latent is of no value
- *[If latent value is set to No Value during Comparison]* Error: Latent value determination has not been selected
- Error: A comparison determination has not been selected
- Error: Individualization determination and latent is of limited value (value for exclusion only)
- Error: A comparison determination has not been selected
- Error: Exclusion determination and exemplar is of no value
- Error: Individualization determination and exemplar is of no value
- Error: A comparison determination has not been selected
- Error: Individualization determination and exemplar is of limited value (value for exclusion only)
- Error: A comparison determination has not been selected
- Error: Exemplar Quality is not painted
- Error: Comparison difficulty has not been selected
- Warning: Individualization determination and fewer than 6 corresponding features marked
- Warning: Individualization determination and no corresponding features marked
- Warning: Individualization determination, but non-corresponding points were also marked

1 Federal Bureau of Investigation; Universal Latent Workstation (ULW) Software.
(https://www.fbibiospecs.org/Latent/LatentPrintServices.aspx)
