# Supplementary material for: Measuring What Latent Fingerprint Examiners Consider Sufficient Information for Individualization Determinations
Source: PLoS One. 2014 Nov 5;9(11):e110179. doi: 10.1371/journal.pone.0110179 (PMC4221158; doi:10.1371/journal.pone.0110179)
Supplement: Appendix S3 — Fingerprint data. (PDF) [file pone.0110179.s003.pdf]

Appendix SI-3 Fingerprint data

The fingerprints for the study were collected at the FBI Laboratory and at Noblis under controlled conditions, and from operational casework datasets collected by the FBI. The collection of fingerprint data from human subjects was approved by the FBI Institutional Review Board and the Noblis Institutional Review Board.

The latents used in the study were selected from a broader set of several thousand latent fingerprints. They were processed using black powder, ninhydrin, physical developer, cyanoacrylate, RUVIS (Reflective Ultra Violet Imaging Systems), RAM Cyanoacrylate fluorescent dye (Rhodamine 6G; Ardrox; 7-p-methoxybenzylamino-4-nitrobenz-2-oxa-1, 3-diazole), or DFO (1,8-diazafluoren-9-one). The processed latents were captured electronically at 8-bit grayscale, uncompressed, at a resolution of 1000 pixels per inch: lift cards and porous materials (processed using ninhydrin or DFO) were scanned using flatbed scanners; latents on nonporous materials (processed using black powder, or cyanoacrylate and light gray powder) were captured using digital cameras.

The exemplars included rolled and plain impressions and were captured as inked prints on paper cards or using FBI-certified livescan devices; exemplars were 8-bit grayscale, 1000 or 500 pixels per inch and were either uncompressed or compressed using Wavelet Scalar Quantization [1]. Nonmated pairs were selected to result in challenging comparisons. They were prepared by down-selecting among exemplar prints returned by searches of the FBI's Integrated AFIS (IAFIS) and among neighboring fingers from the same subject.

The test was initially released to a small subset of the final participants to minimize project risks. Some of these early participants noted a few of the latents were scanned from images that included physical marks indicating minutiae. In response, we reviewed our data and identified seven such images. Each of the seven image pairs was replaced by another pair of the same mating and same preliminary bin classification. The ten responses to the removed image pairs were excluded from all analyses.

The final test design included 320 image pairs (231 mated and 89 nonmated), for an average of five sample image pairs in each of the 64 bins; the actual number of image pairs per bin varied from three to six, as samples of some combinations of fingerprint attributes were relatively difficult to obtain. Some of the latents were used in both mated and nonmated pairs: the 320 image pairs were constructed from 301 latents (from 247 distinct fingers) and 319 exemplars (from 276 distinct fingers). Of the 301 latents, 184 were collected under controlled conditions and 117 were from operational casework. All prints were impressions of distal segments of fingers, including some sides and tips.

The assignments of fingerprint images to examiners were randomized based on an Incomplete Block Design (with examiners as blocks, image pairs as factor levels), balanced to the extent possible using the criterion of D-Optimality, yielding an the experimental design with nearly the same statistical properties as a fully balanced design. Separate designs were constructed for mated and for nonmated pairs. Note that image pairs were used as the factor levels rather than the bins themselves. This decision was based in part on the fact that the same source fingers often contributed to multiple bins and we did not want to assign any one participant two prints from the same finger. Given that (1) there was no "ground truth" for the preliminary bin assignments and those assignments were expected to be imprecise and (2) each examiner was assigned only 22 image pairs from a design space of 64 bins, this seemed a safe approach. After creating the design, we confirmed the validity of this approach: the D-Efficiency, a measure of balance, was calculated for the design with respect to models involving 1-, 2-, 3-, and 4-way interactions of the 4 factors defining the bins. The metric was reasonably close to 100% for models up to 2-way interactions, though somewhat less for models with higher-order interactions.

Early in design we collected fingerprints and assigned those prints to bins before we knew how many participants to expect. Trainees at the FBI were given sets of approximately 25 mated image pairs, informed that they were in fact mated pairs, and asked to quickly characterize each pair per the questions shown in Table S1; one of the authors performed a similar procedure for nonmates. This rapid screening was used for data selection and assignment to bins, not for analysis. Additional prints were collected as needed until we had at least three sample pairs per bin. Late in the design process, after many volunteers had formally expressed interest in the test, we finalized the incomplete block design sized to 210 participants. The number of people who responded to the call for participation, and actually returned results, remained in flux during the testing period.

Can a corresponding area be determined? (if not, there is no overlap, no basis for comparison)
- Yes - there is a potentially corresponding area
- No - there is no overlap, no basis for comparison

Estimated number of minutiae in correspondence
- No minutiae
- 1-4 minutiae
- 5-8 minutiae
- 9-12 minutiae
- 13-20 minutiae
- 21-30 minutiae
- 31+ minutiae

Number of cores present in both prints
- 0
- 1
- 2

Number of deltas present in both prints
- 0
- 1
- 2

Overall clarity of latent
- Low: at least 3/4 of the usable area is limited to ridge flow information; minutiae in this area are "debatable."
- Medium: neither Low nor High (mixed clarity)
- High: the presence or absence of minutiae is certain throughout least 3/4 of the usable area

Is the exemplar quality unusually poor?
- Poor quality exemplar
- Normal exemplar

Is there substantial distortion in the latent? (e.g. twisting, slippage, compressed/stretched ridges)
- High: Distortion is a major factor complicating the comparison
- Medium: Distortion is moderate, but not a major factor complicating the comparison
- Low: Distortion does not complicate the comparison

Does the processing method make interpretation difficult?
- Yes - the processing method makes interpretation particularly difficult
- No

Do the two images have unusually distinctive features in common (e.g., scar, unusual pattern)
- Yes - distinctive features in common
- No

Do the two images have substantial level 3 data in common?
- Yes - substantial level 3 in common
- No

Are there superimpositions, discontinuous impressions or other red flags?
- Yes - there are superimpositions, discontinuous impressions or other red flags
- No

Is either image flipped left-right?
- Yes (flipped)
- No

These two images are supposed to have come from the same finger. If you think this is not correct, please indicate:
- Obvious exclusion (not the same finger)
- Probable exclusion (I am reasonably sure these are not from the same finger)
- Fine as is (no reason to believe these are not the same finger)

Are there any other data problems such as blank or invalid images, multiple impressions where it's not obvious which impression to use, or anything else that we should review for this pair of images?
- Yes (data problem)
- No

Table S1: Questions asked of latent print examiner trainees to screen mated pairs for possible inclusion in the study. Responses to these questions were used to select image pairs for the test and to assign selected image pairs to preliminary bins.

1. Criminal Justice Information Services (1997) Wavelet Scalar Quantization (WSQ) Gray-Scale Fingerprint Image Compression Specification, Version 3.1. (https://www.fbibiospecs.org/docs/WSQ_Gray-scale_Specification_Version_3_1_Final.pdf)
