# Supplementary material for: Measuring What Latent Fingerprint Examiners Consider Sufficient Information for Individualization Determinations
Source: PLoS One. 2014 Nov 5;9(11):e110179. doi: 10.1371/journal.pone.0110179 (PMC4221158; doi:10.1371/journal.pone.0110179)
Supplement: Appendix S4 — Post-processing of response data. (PDF) [file pone.0110179.s004.pdf]

Appendix SI-4 Post-processing of response data

For each image pair assigned to an examiner, two data files were produced: one saved upon completion of the Analysis phase (before the exemplar print was presented) and a second after completion of Comparison/Evaluation phase. The files complied with the ANSI/NIST-ITL [1] standard, using the COMP transaction described in the Latent Interoperability Transmission Specification [2]. All collected data was included in standard fields, with the exception of the comparison difficulty, the exclusion reason (collected in temporary fields), and the temporary lines (not used for evaluation; field expected to be added to a 2013 revision of the standard).

Upon receipt, the files were decrypted, images were reinserted, and the contents were validated. Validation included some of the processing steps below to perform a cursory check that the data was intact and appeared to contain complete annotation and determinations.

Post-processing software derived tabular data from the ANSI/NIST COMP files. Initially, summary data was produced for each fingerprint (latent and exemplar separately) and separately for each feature annotated. The summary data for a fingerprint includes counts of features with some breakdown by feature type and the local clarity for the features, various measures pertaining to the area annotated at each clarity level, and the categorical responses (determinations and difficulty). The summary data for each feature includes its type, clarity, and location; data for features annotated as corresponding between the latent and exemplar were linked. For a full description of the available annotation data (feature types, correspondence types, clarity levels, etc.), see the test Instructions (Appendix SI-20).

The summary data describing clarity annotation includes the area at each clarity level, measures of largest contiguous area at each clarity level, and related measures [3]. For each type of annotation (latent Analysis, latent and exemplar Comparison, and derived corresponding clarity) median clarity maps were produced. Each median clarity map represents the median clarity at each (x, y) location on an image as calculated from a set of clarity maps produced by individual examiners for the same image. The set of clarity maps used to produce the median maps excludes responses from four examiners whose clarity annotation did not comply with the test instructions. The median clarity maps conformed well to our expectations of proper and careful characterizations of latent clarity, by reducing the impact of outlier opinions and imprecision.

The tabular summary response data was joined with data from the experimental design, including in particular whether or not the image pairs were mated, and also meta-information about the fingerprint images and the screening bins used to balance the assignments. These tables, together with the actual images and visualization of the annotation, were the primary basis for analyses. Additional tools and information were used to identify and analyze outliers.

Numerous anomalies were observed in the examiner response files. Most of these pertained to specific aspects of the annotation and often there was no objective criterion for differentiating extreme, but legitimate responses from outright errors and failures to comply with specific instructions. Most of these anomalous annotations were perfectly usable in most regards. Some common examples include annotating nonminutia features as minutiae (the default feature type), failing to expressly correspond pairs of features that were annotated in both the latent and exemplar, and using clarity colors improperly (e.g., annotating debatable ridge flow as background). We chose to keep records of anomalies we discovered and be cognizant of such outliers when interpreting the results rather than exclude them from analysis. Anomalous responses were sometimes excluded from voted metrics. For example, many examiners never annotated cores or deltas, so these examiners were excluded from the construction of metrics describing the proportion of examiners who marked cores or deltas. As a rule, the percentage of responses containing problematic measurements was sufficiently low as to have limited deleterious effects on the models; also, robust statistics, such as median, were often selected to limit these effects and help interpret the data.

Statistical analyses and modeling were conducted with SAS JMP® version 10 [4].

1 National Institute of Standards (2011) American National Standard for Information Systems: Data format for the interchange of fingerprint, facial & other biometric information. ANSI/NIST-ITL 1-2011. (http://fingerprint.nist.gov/standard)

2 Chapman W, et al (2013) Latent Interoperability Transmission Specification. NIST Special Publication 1152. US Department of Commerce, National Institute of Standards and Technology, Washington, DC. (http://nvlpubs.nist.gov/nistpubs/SpecialPublications/NIST.SP.1152.pdf)

3 Hicklin RA, Buscaglia J, Roberts MA (2013) Assessing the clarity of friction ridge impressions. Forensic Sci Int 226(1):106-117.

4 JMP® statistical software, SAS Institute Inc., Cary, NC, USA, Copyright 2012 (www.jmp.com)
