# Supplementary material for: Measuring What Latent Fingerprint Examiners Consider Sufficient Information for Individualization Determinations
Source: PLoS One. 2014 Nov 5;9(11):e110179. doi: 10.1371/journal.pone.0110179 (PMC4221158; doi:10.1371/journal.pone.0110179)
Supplement: Appendix S5 — Summary of examiner determinations. (PDF) [file pone.0110179.s005.pdf]

Appendix SI-5 Summary of examiner determinations

We received data from 172 examiners: one examiner did not take the test seriously and commented that the results should be disregarded; another examiner returned responses to practice problems only. This resulted in a total of 3740 responses received and fully processed (170 examiners x 22 assignments) for each of Analysis and Comparison. As discussed in Appendix SI-3, responses on seven image pairs were excluded, yielding a total of 3730 valid responses from the Analysis phase (mean 12.4 examiners per latent).

For our analyses of mated pairs, we required valid annotations and determinations from both phases. Among the 2882 responses to mated pairs (see Table S2), we additionally excluded one file that was missing a Comparison determination and all responses from five examiners who routinely did not annotate correspondences. This resulted in 2796 valid responses (mean 12.1 responses per mated pair). Some analyses omit the 125 erroneous exclusions, resulting in 2671 mated image pairs. We did not omit any additional responses that we considered to be improperly annotated.

Comparison Determination	Latent Value	Total	Mates (Invalid)	Mates (valid)	Nonmates (all)
	NV (in Analysis)	713	21	441	251
	NV (in Comparison)	43	1	28	14
Exclusion	VEO	131	2	27	102
Exclusion	VID	430	4	98	328
Inconclusive	VEO	359	5	268	86
Inconclusive	VID	346	6	275	65
Individualization	VID	1700	46	1653	1
(Exemplar NV)	VEO	3	0	3	0
(Exemplar NV)	VID	3	0	3	0
(invalid determination)	VEO	1	0	0	1
(invalid determination)	VID	1	1	0	0
Totals		3730	86	2796	848
Total comparisons	VCMP (either VEO or VID)	2974	64	2327	583
Total comparisons	VID	2480	57	2029	394

Table S2: Final determinations by 170 examiners on 320 image pairs. The latent value column reports the final value determination (after any Comparison phase changes in value assessments).

Fig. S1 shows that the sample fingerprints selected for the test were well balanced with respect to our objective of better understanding what constitutes sufficiency for individualization. The majority of latents were of marginal value for individualization but included a small proportion in which examiners were unanimous in their determinations. Likewise, the mated image pairs were predominantly of marginal sufficiency for individualization and there were samples at either extreme. This distribution ensured an efficient design for answering our research questions. One effect of this design (focusing on marginal cases), is that the rates of interexaminer reproducibility of determinations were reduced relative to our previous Black Box study [1].

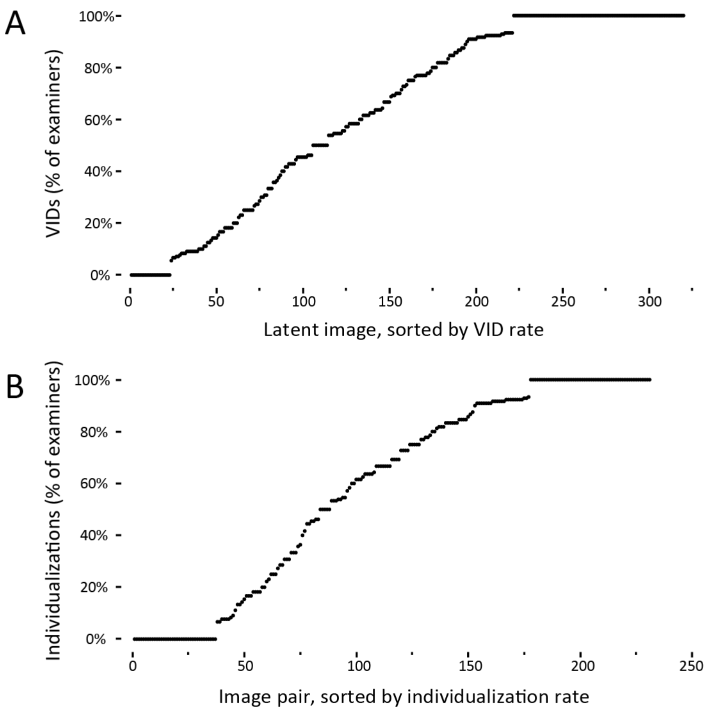

Fig. S1: Reproducibility of latent value and individualization determinations. Percentage of examiners (y-axis) who made (A) VID determinations for each latent (x-axis) and (B) Individualization determinations for each mated pair (x-axis). Includes 3730 latent value determinations from 170 examiners on 301 latents, and 2796 responses by 165 examiners on 231 mated pairs.

This data sampling strategy is neither designed, nor well-suited, for estimating operational error rates; however, we note that the error rates on this test were similar to those we measured in our Black Box study. The single false positive error (among 583 nonmated comparisons) was consistent with the rate of 6/4083 observed in the Black Box study [2]. The false negative error rate was lower on this test (5.5% of mated comparisons vs. 7.5% in the Black Box study). In addition to differences in data sampling, other factors might have contributed to this lower false negative error rate: the Black Box study alerted the fingerprint community to a high false negative rate, so the examiners may have modified their behavior; the process of providing detailed markup may have influenced some of the determinations; there were some differences in participants between the two studies, notably a greater number of non-U.S. participants in this study.

1 Ulery BT, Hicklin RA, Buscaglia J, Roberts MA (2012), Repeatability and Reproducibility of Decisions by Latent Fingerprint Examiners. PLoS ONE 7:3. (http://www.plosone.org/article/info:doi/10.1371/journal.pone.0032800)

2 Ulery BT, Hicklin RA, Buscaglia J, Roberts MA (2011) Accuracy and reliability of forensic latent fingerprint decisions. Proc Natl Acad Sci USA 108(19): 7733-7738. (http://www.pnas.org/content/108/19/7733.full.pdf)
