# Supplementary material for: Measuring What Latent Fingerprint Examiners Consider Sufficient Information for Individualization Determinations
Source: PLoS One. 2014 Nov 5;9(11):e110179. doi: 10.1371/journal.pone.0110179 (PMC4221158; doi:10.1371/journal.pone.0110179)
Supplement: Appendix S6 — Timing. (PDF) [file pone.0110179.s006.pdf]

Appendix SI-6 Timing

Fig. S2 summarizes the time taken by examiners for Analysis and Comparison: 80% of examiners took 3 to 26 minutes per image for latent analysis; 80% of examiners took 5 to 46 minutes per image pair for comparison. Some examiners reported spending up to about 20 hours to complete the study. Times are based on the examiner's opening and closing of images for Analysis or image pairs for Comparison. Examiners were allowed to revisit work; hence, elapsed times were constructed as totals across multiple sessions. Interruptions, such as phone calls or lunch breaks may have substantially affected many of these measurements, so they should be taken as upper bounds on the time actually taken for examination itself. Examiners were not informed that timing measurements were being recorded.

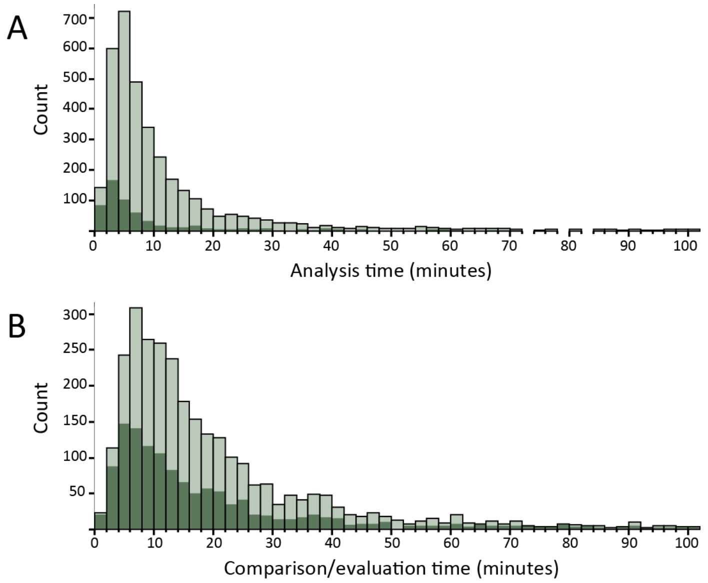

Fig. S2: Elapsed times in minutes for (A) Analysis phase, NV determinations highlighted; and (B) Comparison/Evaluation phase, non-individualization determinations highlighted.
