# Supplementary material for: Measuring What Latent Fingerprint Examiners Consider Sufficient Information for Individualization Determinations
Source: PLoS One. 2014 Nov 5;9(11):e110179. doi: 10.1371/journal.pone.0110179 (PMC4221158; doi:10.1371/journal.pone.0110179)
Supplement: Appendix S7 — Participant background survey responses. (PDF) [file pone.0110179.s007.pdf]

Appendix SI-7 Participant background survey responses

The following table summarizes responses to the background survey, which participants completed after submitting their test data. One participant did not complete the survey, so responses total 169.

	Count	*%*
**1. Sex**		
• Female	98	58.0%
• Male	71	42.0%
**2. Age**		
• 18-29	28	16.6%
• 30-39	71	42.0%
• 40-49	29	17.2%
• 50-59	30	17.8%
• 60-69	11	6.5%
**3. Highest level of education achieved**		
• High School Diploma/GED	13	7.7%
• Associate Degree / some college	20	11.8%
• Bachelor's Degree	76	45.0%
• Graduate Degree / Professional Degree	60	35.5%
**4. Current Employment**		
• U.S. Federal government	71	42.0%
• U.S. State government	24	14.2%
• U.S. City/County government	36	21.3%
• U.S. Private sector (non-government)	7	4.1%
• Non-U.S. National/Federal government	24	14.2%
• Non-U.S. State/Provincial government	5	3.0%
• Non-U.S. Local/ City/County government	1	0.6%
• Non-U.S. Private sector (non-government)	1	0.6%
**5. Has your agency received accreditation in latent prints?**		
• Yes - for example, by American Society of Crime Laboratory Directors/Laboratory Accreditation Board (ASCLD/LAB), Forensic Quality Services (FQS), or International Organization for Standardization (ISO/IEC 17025)	113	66.9%
• No	50	29.6%
• Don't know	6	3.6%
**6. Total number of years employed as a latent examiner**		
• Less than 1	0	0.0%
• 1-4	40	23.7%
• 5-9	59	34.9%
• 10-19	37	21.9%
• 20-29	20	11.8%
• 30-39	11	6.5%
• 40 or more	2	1.2%
**7. Type of latent training received**		
• Formal program of instruction for 1 year or more	106	62.7%
• Formal program of instruction for 6 months to 1 year	35	20.7%
• Limited formal training (courses, workshops) for less than 6 months	19	11.2%
• Other	9	5.3%

	Count	%
**8. Are you certified as a latent print examiner? (Check all that apply - may add up to more than 100%)**		
• International Association for Identification (IAI) Certified Latent Print Examiner (CLPE)	56	33.1%
• Certified or qualified as a latent print examiner by a current or previous employer	97	57.4%
• National certification (non-US only)	19	11.2%
• Other certification	5	3.0%
• No certification	19	11.2%
**9. Are you currently conducting latent examinations on a regular basis (at least weekly over an extended period?)**		
• No	2	1.2%
• No, but I have previously conducted latent examinations on a regular basis	16	9.5%
• Yes	151	89.4%
**10. What percentage of time have you spent over the last year doing latent comparisons?**		
• None: I am not performing comparisons	0	0.0%
• Less than 10%	12	7.1%
• 10-25%	21	12.4%
• 25-50%	23	13.6%
• 50-75%	46	27.2%
• 75-100%	67	39.6%
**11. Of the latent-to-exemplar comparisons you have performed over the last year, what proportion do you perform on computer screens, as opposed to looking at physical evidence/paper cards?**		
• 0% computer	12	7.1%
• 1-30% computer	68	40.2%
• 30-60% computer	35	20.7%
• 60-99% computer	42	24.9%
• 100% computer	12	7.1%
**12. Of the latent-to-exemplar comparisons you have performed over the last year, what proportion of the conclusions were based on a single exemplar print (e.g. based on the rolled exemplar without reference to the plain exemplar or additional sets of exemplar fingerprints)?**		
• None	13	7.7%
• Less than 10%	48	28.4%
• 10-25%	16	9.5%
• 25-50%	23	13.6%
• 50-75%	40	23.7%
• 75-100%	29	17.2%
**13. Does your organization permit an official conclusion of less than individualization, more than inconclusive, such as "limited match" or "qualified identification"? (Given the standard operating procedures that you/your agency currently use)**		
• No	149	88.2%
• Yes	20[1]	11.8%
**14. In determining the value/sufficiency of a latent impression, how do you define an impression that is not suitable for individualization but could potentially be used for exclusion? (Given the standard operating procedures that you/your agency currently use)**		
• It has its own category used in standard practice, such as "Of value for exclusion only" or "Limited value"	33	19.5%
• It has its own category, such as "Of value for exclusion only" or "Limited value" - but only used upon request	42	24.9%
• No value	81	47.9%
• Of value	13	7.7%

[1] *Eleven of these were from non-U.S. examiners.*

	Count	%
**15. Do you have a different value threshold for AFIS searches than for non-AFIS casework, such as "AFIS quality" or "of value for AFIS"? (Given the standard operating procedures that you/your agency currently use)**		
• No	53	31.4%
• Yes, used informally	70	41.4%
• Yes, used as formal designation	46	27.2%
**16. Are the latent prints in the White Box Study similar to those that you might encounter in casework?**		
• Some of them	20	11.8%
• Most of them	47	27.8%
• All but one or two	6	3.6%
• All of them	96	56.8%
**17. Are the exemplar prints in the White Box Study similar to those that you might encounter in casework?**		
• Some of them	14	8.3%
• Most of them	45	26.6%
• All but one or two	9	5.3%
• All of them	101	59.8%
**18. Overall, how do the comparisons in the White Box study compare to your casework? In other words, is the distribution of difficulty of the comparisons in the White Box study similar to the distribution of difficulty in your casework?**		
• MUCH EASIER than casework	4	2.4%
• EASIER than casework	11	6.5%
• SIMILAR to casework	128	75.7%
• HARDER than casework	25	14.8%
• MUCH HARDER than casework	1	0.6%
**19. Would you consider using markup such as this for actual casework?**		
• No	36	21.3%
• Possibly	42	24.9%
• Yes, for general practice	39	23.1%
• Yes, Only for complex cases	52	30.8%
**20. If your country or agency has a minimum number of minutiae required to make an individualization (or identification) decision, please indicate that minimum point standard here. (Text response)[2]**		
• No point standard (no response or said they had no point standard for individualization)	150	88.8%
• 16-point standard	2	1.2%
• 12-point standard	11	6.5%
• 10-point standard "for a criminal case"	1	0.6%
• 8-point standard	3	1.8%
• 7-point standard	2	1.2%

[2] *See Appendix SI-18 for discussion of how the point standard text responses were categorized for analysis.*
