# Supplementary material for: Measuring What Latent Fingerprint Examiners Consider Sufficient Information for Individualization Determinations
Source: PLoS One. 2014 Nov 5;9(11):e110179. doi: 10.1371/journal.pone.0110179 (PMC4221158; doi:10.1371/journal.pone.0110179)
Supplement: Appendix S8 — Associations between examiners' annotations and their determinations. (PDF) [file pone.0110179.s008.pdf]

Appendix SI-8 Associations between examiners' annotations and their determinations

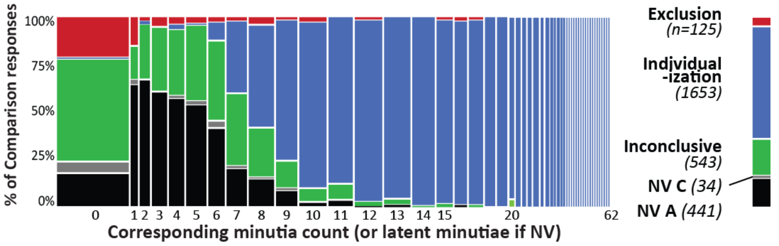

Fig. S3: Associations of corresponding minutia counts and determinations (n=2796 responses by 165 examiners on 231 mated image pairs). This differs from Figure 3B by including the Analysis phase minutia count for NV latents rather than showing zero corresponding minutia count. "NV A" denotes NV in the Analysis phase; "NV C" denotes NV in the Comparison phase.

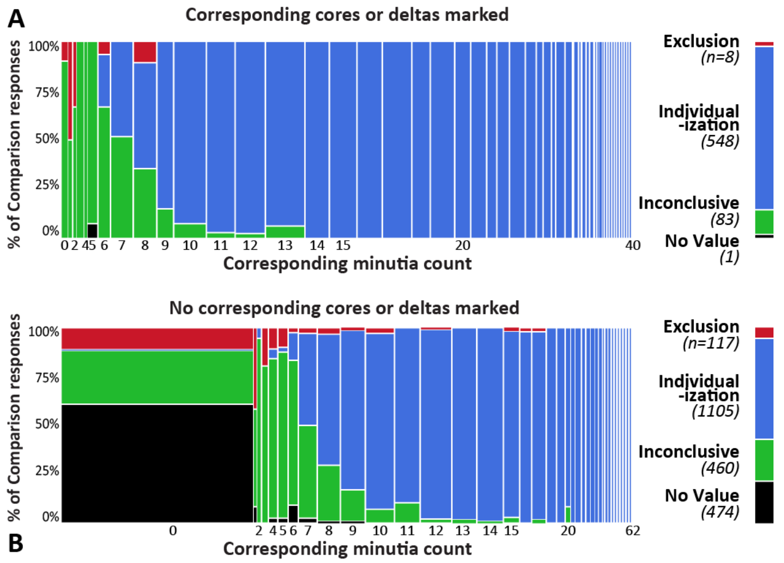

Fig. S4: Associations of corresponding minutia counts and determinations by 165 examiners on 231 mated image pairs, conditioned on (A) at least one corresponding core or delta marked (n=640 responses); and (B) no corresponding cores or deltas marked (n=2156 responses). NV decisions from the Analysis phase are treated as having zero corresponding minutiae.

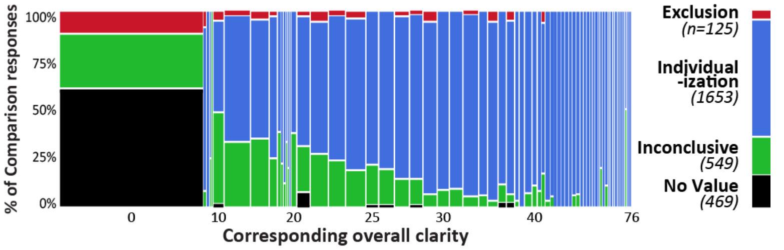

Fig. S5: Associations of corresponding Overall Clarity and determinations (n=2796 responses by 165 examiners on 231 mated image pairs).
