# Supplementary material for: Measuring What Latent Fingerprint Examiners Consider Sufficient Information for Individualization Determinations
Source: PLoS One. 2014 Nov 5;9(11):e110179. doi: 10.1371/journal.pone.0110179 (PMC4221158; doi:10.1371/journal.pone.0110179)
Supplement: Appendix S9 — Low count individualizations. (PDF) [file pone.0110179.s009.pdf]

Appendix SI-9 Low count individualizations

Table S5 details our review of the individualization determinations with fewer than seven corresponding minutiae. In all of the annotations of image pairs with fewer than five corresponding minutiae marked, examiners marked but did not link (correspond) additional features (improper annotation). Most of the image pairs with five or six corresponding minutiae marked included additional nonminutia features. After discounting the outliers that we believe were due to improper annotation, we did find examples of individualizations with as few as six corresponding minutiae or five minutiae with two level-3 points marked on incipient ridges.

Image Pair	Corresponding Features				ID Rate	Median corresponding minutiae among IDs (overall)	Our comments on the annotation
	Minutiae	Cores, Deltas	Other Features	Debatably Corresponding Features			
CW083	0	0	0	0	100%	15 (15)	13 apparently corresponding minutiae were marked but not linked
*CW262	0	0	0	1	100%	14 (14)	10 apparently corresponding minutiae were marked but not linked; obvious errors in using software
*CW167	2	0	0	8	100%	19 (19)	a total of 15 apparently corresponding minutiae marked, but obvious errors in using software to link
CW156	4	0	0	0	75%	10 (9.5)	7 apparently corresponding incipient ridge features were marked but not linked (in green clarity area)
CW306	4	0	0	0	64%	10 (9)	corresponding delta & its features not marked (in blue clarity area)
CW173	5	0	2	0	53%	8 (7)	small area of green clarity
CW022	6	0	0	0	55%	9 (7)	3 apparently corresponding minutiae were marked but not linked (in yellow clarity area)
CW081	6	0	4	3	45%	8 (6)	
CW141	6	0	0	0	8%	6 (0)	this examiner made the sole ID for this image pair (out of 12)
CW154	6	0	0	0	79%	9 (7.5)	
CW185	6	1	0	5	64%	13 (12)	
CW201	6	1	0	0	36%	6.5 (5)	
CW201	6	0	0	0	36%	6.5 (5)	core not marked; 3 apparently corresponding minutiae marked (in green clarity area), but not linked
CW218	6	1	0	0	23%	17 (5)	minimal overlap
CW249	6	0	0	3	91%	9.5 (10)	
CW326	6	1	0	0	44%	12.5 (3)	

* Image pair comparisons were by the same examiner.

Table S5: Descriptions of individualizations with fewer than seven corresponding minutiae marked; comments are based on the authors' manual review of the original annotations. In some cases, features were marked in the latent and exemplar that clearly corresponded, but the pairs were not explicitly linked by the examiner. Not linking features could have been an oversight or a deliberate expression of uncertainty. This table excludes five examiners who did not correspond features.
