# Supplementary material for: Measuring What Latent Fingerprint Examiners Consider Sufficient Information for Individualization Determinations
Source: PLoS One. 2014 Nov 5;9(11):e110179. doi: 10.1371/journal.pone.0110179 (PMC4221158; doi:10.1371/journal.pone.0110179)
Supplement: Appendix S10 — Reproducibility of individualization determinations. (PDF) [file pone.0110179.s010.pdf]

Appendix SI-10 Reproducibility of individualization determinations

Predictors		DF	AICc	R^2	Gen R^2	Misclass.	AUC
None		0	312	0.0000	0.0000	0.3976	0.5000
CD>0		1	303	0.0378	0.0671	0.3976	0.5943
Difficulty		5	255	0.2185	0.3443	0.2632	0.7926
OverallClarity		1	241	0.2379	0.3703	0.2370	0.8143
OverallClarity + CMin		2	223	0.3024	0.4518	0.2088	0.8483
Determination	*{Individualization, Insufficient}*	1	237	0.2492	0.3850	0.2054	0.7819
CMin		1	225	0.2877	0.4339	0.2035	0.8408
CMin_green + CMin_yellow		2	222	0.3037	0.4534	0.2000	0.8497
CMin + Difficulty		6	226	0.3175	0.4699	0.1992	0.8565
Determination + CMin		2	224	0.2981	0.4466	0.1995	0.8428

Table S12: Logistic regression models predicting whether a second examiner will individualize {Indiv, Insuff} based on first examiner's response (n=2671). Constructed from all distinct pairs of examiners on each mated image pair; each image pair weighted equally (n=231).

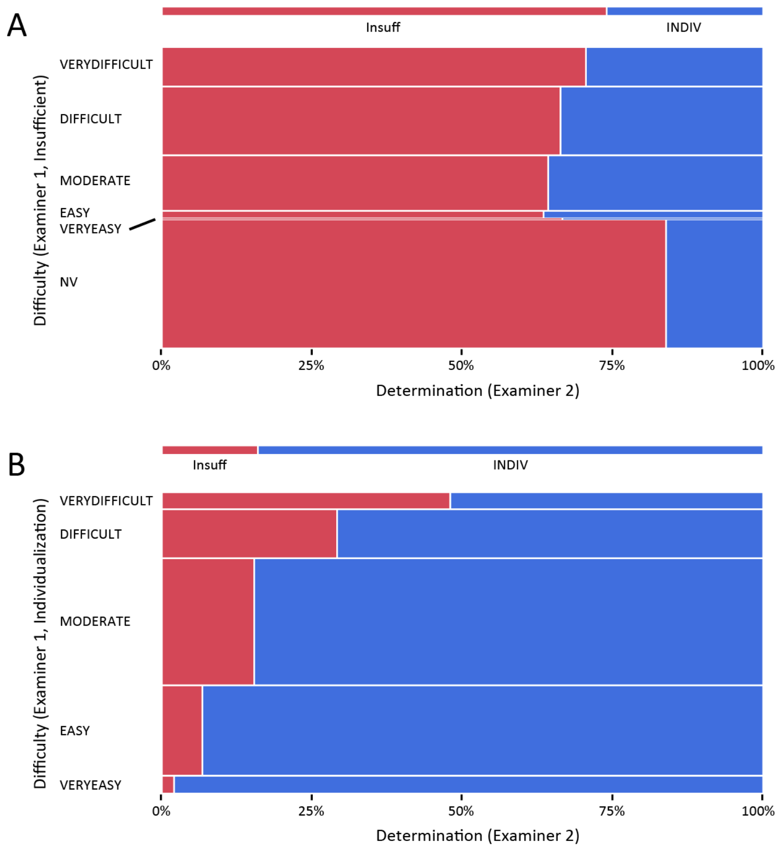

Fig. S19: Reproducibility of sufficiency decisions by difficulty. Mosaic charts showing proportions of examiners who reproduced determinations (x-axis), conditioned on the determination and difficulty assessment of another examiner who determined (A) insufficient or (B) individualization. Chart A: n=1018 insufficient determinations on 168 mated pairs (10,927 paired examiner responses). Chart B: n=1653 individualization determinations on 194 mated pairs (constructed from 18,289 paired examiner responses).
