# Supplementary material for: Measuring What Latent Fingerprint Examiners Consider Sufficient Information for Individualization Determinations
Source: PLoS One. 2014 Nov 5;9(11):e110179. doi: 10.1371/journal.pone.0110179 (PMC4221158; doi:10.1371/journal.pone.0110179)
Supplement: Appendix S11 — Models of latent value sufficiency. (PDF) [file pone.0110179.s011.pdf]

Appendix SI-11 Models of latent value sufficiency

The data on latent value sufficiency contributes to our understanding of sufficiency for individualization in two important ways:

- When modeling sufficiency for individualization, our measures of corresponding clarity and corresponding feature counts were often zero because responses to image pairs were not provided when the latent was determined to be NV. The Analysis phase data is sometimes easier to interpret than the Comparison/Evaluation phase data because it is neither conditioned on a prior response (Analysis determination), nor on the examiner's ability to align the latent and exemplar prints. The associations between Analysis annotations and VID are very similar to the associations between Comparison annotations and individualizations.
- In a previous study [1], we reported similar results for latent value determinations. The data collected in this experiment includes examiner effects (many responses to each latent), a much larger sample of latents that are of borderline value, and more independent variation in latent clarity and minutia counts. These differences allow us to observe additional and more subtle effects. These results generally support our previous findings.

The following analysis is based on logistic regression models describing the association between examiner markup and determinations by the same examiner of latent value for individualization (VID) and value for comparison (VCMP); n=3730 responses to 301 latents by 170 examiners.

Examiners rated most latents VID. Therefore, the percentage of latent determinations that were not VID (35.1%) represents the base misclassification rate (see Table S7). Predicting individual VID determinations based on the majority determination for each latent reduces the misclassification rate to 15.5%. This latter statistic represents a theoretical limit to how well any model based exclusively on properties of the latents can perform on this dataset. This limit is imposed by the lack of agreement among examiners. Both of these reference numbers reflect our deliberate selection of data for the test: we wanted abundant data from the Comparison phase, and we concentrated data selection on borderline cases in order to measure decision thresholds (see Fig. S1A). The corresponding statistics for VCMP determinations are 19.1% base misclassification rate, and 11.3% theoretical limit for models based exclusively on latent characteristics.

A simple model based on the median number of minutia that examiners marked on the latents performs close to the theoretical limits, with misclassification rates of 17.0% (VID) and 13.7% (VCMP). This strong performance leaves little room for improving the model using other descriptors of the latents, such as measures of clarity. Median minutia count, which is a constant for each latent, captures none of the variability due to differences among examiners, interaction effects between examiners and latents, or any other factors contributing to disagreements; it is based exclusively on properties of the latents. Further substantial improvements to the models must account for these other sources of variability.

Using each examiner's minutia count to predict that examiner's latent value determination, results in misclassification rates of 7.3% (VID) and 11.5% (VCMP). The misclassification rate for VID is much lower than the theoretical limit for any metric describing only the latent. That is, individual examiners' counts and value determinations co-vary to an extent beyond what can be explained by properties of the latent.

- Much of the variability (disagreement) in value determinations is associated with variability (disagreement) in minutia counts.
- One implication is that "minutia count" must be understood as a subjective measure (depending on the examiner) and not strictly as a property of the latent.

Examiners' counts are subjective and biased in relation to their determinations. Insofar as this bias is captured in the median minutia count, one might describe the *median* as a "subjective" measure. It is important to understand, however, that this subjectivity pertains to which properties of the latent are actually being measured and does not pertain to variability among examiner responses.

Examiner subjectivity may manifest in various ways, such as a general tendency toward (or from) VID determinations, conformance to a point standard, or tending to mark more (or fewer) features. This experiment was not specifically designed to resolve these questions, but offers some insight.

- The *Latent + Examiner* model gives misclassification rates of 8.2% (VID) and 5.9% (VCMP) [DF=469]. Adding examiners' subjective minutia counts substantially reduces the misclassification rates to 3.0%

(VID) and 2.7% (VCMP). This demonstrates that minutia count captures a substantial proportion of the Latent-Examiner interaction effects (as opposed to merely additive effects due to general examiner tendencies). Any overfitting (due to the 469 degrees of freedom) would bias all four of these estimates downward, potentially giving a misleadingly low indication of the remaining lack of repeatability.

- Individualizations were frequently made with fewer than 12 marked features. As a group, the 10 examiners following a 12-point standard actually rated a slightly greater proportion of latents VID than did other examiners: 157/220 (75%) vs. 2263/3510 (65%) [unconvincing p=0.0381]. We have no evidence that this is causally related to the point standard itself, as this variable is confounded with other effects that were not controlled in the experimental design.
- The mere association between number of minutiae marked and value determinations does not necessarily imply a causal relation. The data appear consistent with various possible explanations. Determinations might methodically follow Analysis phase annotations; however, alternatively, a preliminary determination (possibly subconscious) might influence how an examiner marks a latent.

The following models describing latent value determinations extend our previous work [1]. The fingerprints in this study were selected to include a much higher proportion rated NV or VEO; they were selected to more easily discriminate effects of feature counts, image clarity, complexity, and the presence of cores and deltas. We also obtained multiple examiner responses to each latent in order to investigate subjectivity in examiner determinations. The fingerprints in this study were concentrated more toward those characteristics that would be of borderline sufficiency; this resulted in lower rates of agreement on value determinations than measured in our Black Box study [2]. Examiners were unanimous on whether 118/301 (39.2%) of the latents were VID (23 Not VID; 95 VID), with a mean percentage agreement of 77.5%. Examiners were unanimous on whether 151/301 (50.2%) of the latents were of value for comparison (5 NV; 146 CMP), with a mean percentage agreement of 81.8%.

In the following tables, the Predictors column describes the independent variables used in logistic regression models predicting examiner latent value determinations {VID, Not VID} and {VCMP, NV}. Each model was fit to 3730 responses by 170 examiners on 301 latents. In these models, all terms are additive; asterisks denote cross-product terms expressing interactions between pairs of explanatory variables. Table S7 summarizes explanatory models describing causal relations between the stimuli (in this case, image and examiner) and the response (determination): the models also describe the contribution of fixed attributes of each image such as median minutia count. The models in Table S8 describe associations between examiners' annotations and value determinations (same examiner); these models do not presume causality.

General caution: statistical measures such as the corrected Akaike Information Criterion (AICc), Generalized R^2 and p-values (not shown, but considered in model selection) assume that the 3730 responses are independent. This assumption is not valid because each examiner and image contributed to multiple responses; as a result, these statistics may be substantially biased (to indicate the models are better than they really are). Such biases were considered when selecting models for inclusion in the tables. Dozens of additional variants of these models were fitted and generally yielded similar results. Such models included alternate measures of features and clarity (such as largest contiguous areas at each level of clarity), cross terms and transforms of terms.

**Latent**	Nominal variable identifying the latent (n=301)
**Examiner**	Nominal variable identifying the examiner (n=170)
**MC1, .., MC5**	Areas of red, yellow, green, blue, aqua from median clarity map
**CD_rate**	Continuous variable indicating of the proportion of examiners who marked at least one core or delta (138 voting examiners)
**cdm**	Total number of minutiae, cores, and deltas marked by this examiner
**green_MC**	Area of green or higher clarity from median clarity map = AA3 + AA4 + AA5
**green_MC_LCA**	Largest contiguous area of green or higher clarity from median clarity map
**Min_green**	Minutiae that this examiner annotated as green or higher clarity
**Min_green_MC**	Minutiae of this examiner in green or higher clarity from median clarity map
**green**	Area of green or higher clarity annotated by this examiner = A3 + A4 + A5
**MedianMin**	Median(Min) across all responses to the same latent
**Min**	Minutia count for the latent print
**OC**	Overall Clarity from this examiner's annotation
**OC(MC)**	Overall Clarity from median clarity map
**PtStd**	Whether the examiner followed a 12-point standard
**YY**	Area of yellow or higher clarity from median clarity map = AA2 + AA3 + AA4 + AA5

Table S6: Legend of variables used to predict latent value determinations.

In these tables, DF= degrees of freedom; R^2 = entropy R^2; AICc = corrected Akaike Information Criterion (AICc); Gen R^2=Generalized R^2; Misclass= misclassification rate; AUC = area under the (receiver operating characteristic) curve.

Predictors	DF	VID					VCMP				
		AICc	R^2	Gen R^2	Misclass	AUC	AICc	R^2	Gen R^2	Misclass	AUC
None (base rate)	0	4838	0.0000	0.0000	0.3512	0.5000	3642	0.0000	0.0000	0.1912	0.5000
Examiner	169	4769	0.0874	0.1474	0.3201	0.6923	3593	0.1108	0.1644	0.1906	0.7245
Latent	300	3005	0.5141	0.6696	0.1547	0.9278	2458	0.5046	0.6240	0.1134	0.9349
Latent; Examiner	469	2447	0.7164	0.8327	0.0815	0.9772	2079	0.7245	0.8134	0.0590	0.9820
CD_rate	1	4561	0.0576	0.0990	0.3619	0.6596	3176	0.1285	0.1891	0.1912	0.7450
CD_rate; Examiner	170	4458	0.1521	0.2464	0.2960	0.7564	3099	0.2470	0.3437	0.1788	0.8363
MC1; MC2; MC3; MC4; MC5	5	3475	0.2838	0.4237	0.2080	0.8474	2657	0.2732	0.3756	0.1657	0.8543
green_MC	1	3506	0.2757	0.4137	0.2169	0.8444	2730	0.2511	0.3488	0.1818	0.8395
log(YY); green_MC; log(YY)*green_MC	3	3326	0.3139	0.4601	0.2105	0.8562	2607	0.2858	0.3906	0.1595	0.8564
green_MC_LCA	1	3469	0.2835	0.4233	0.2113	0.8476	2722	0.2532	0.3513	0.1826	0.8397
OC(MC)	1	3427	0.2920	0.4338	0.2121	0.8506	2682	0.2643	0.3648	0.1708	0.8503
OC(MC); Examiner	170	3171	0.4184	0.5763	0.1759	0.8993	2526	0.4045	0.5234	0.1332	0.9054
MedianMin	1	2784	0.4250	0.5831	0.1697	0.9014	2239	0.3860	0.5037	0.1367	0.8988
MedianMin; CD_rate	2	2785	0.4252	0.5833	0.1697	0.9012	2132	0.4159	0.5353	0.1196	0.9079
MedianMin; PtStd	2	2798	0.4265	0.5846	0.1697	0.9019	2240	0.3861	0.5038	0.1357	0.8989
MedianMin; OC(MC)	2	2753	0.4318	0.5901	0.1705	0.9042	2222	0.3912	0.5093	0.1351	0.9012
MedianMin; Median(Min_green_MC)	2	2735	0.4356	0.5939	0.1702	0.9061	2232	0.3885	0.5063	0.1386	0.9002
MedianMin; YY; green_MC	3	2739	0.4353	0.5936	0.1681	0.9063	2232	0.3890	0.5069	0.1351	0.9007
MedianMin; Examiner	170	2355	0.5872	0.7335	0.1142	0.9517	1950	0.5626	0.6780	0.0909	0.9522

Table S7: Latent value determination as a dependent response to (A) the image pairs and examiners; (B) attributes of the image pairs as estimated by median statistics (n=3730). These models are intended to address questions of causality and therefore do not include same-examiner associations between the predictor variables and the determinations. Predictors such as MedianMin do not vary by image and therefore describe something about the image itself (albeit something about examiners' collective responses to the image).

Predictors	DF	VID					VCMP				
		AICc	R^2	Gen R^2	Misclass	AUC	AICc	R^2	Gen R^2	Misclass	AUC
None (base rate)	0	4838	0.0000	0.0000	0.3512	0.5000	3642	0.0000	0.0000	0.1912	0.5000
CD	1	4717	0.0254	0.0445	0.3512	0.5873	3328	0.0867	0.1302	0.1912	0.6571
CD; Examiner	170	4631	0.1163	0.1927	0.3048	0.7252	3289	0.1948	0.2779	0.1863	0.8004
cdm; points	2	2088	0.5694	0.7185	0.1134	0.9463	1813	0.5036	0.6230	0.1099	0.9409
green	1	3828	0.2091	0.3268	0.2466	0.8052	2879	0.2102	0.2976	0.1912	0.8100
OC	1	3771	0.2211	0.3430	0.2504	0.8148	2867	0.2134	0.3016	0.1858	0.8228
OC; CD_rate	2	3607	0.2553	0.3879	0.2255	0.8316	2491	0.3172	0.4272	0.1552	0.8724
OC; Examiner	170	3408	0.3693	0.5237	0.1834	0.8836	2673	0.3642	0.4800	0.1440	0.8908
Min	1	2044	0.5782	0.7260	0.1150	0.9486	1895	0.4804	0.6005	0.1147	0.9348
Min; CD_rate	2	2027	0.5821	0.7293	0.1126	0.9499	1686	0.5385	0.6560	0.0962	0.9476
Min; Examiner	170	1672	0.7284	0.8411	0.0617	0.9793	1538	0.6758	0.7749	0.0617	0.9752
Min; Examiner; (Examiner*Min)	339	1661	0.8114	0.8957	0.0601	0.9891	1630	0.7577	0.8386	0.0598	0.9854
Min; Examiner; CD_rate	171	1654	0.7325	0.8439	0.0598	0.9799	1312	0.7386	0.8243	0.0469	0.9839
Min; Min_green	2	1985	0.5908	0.7365	0.1083	0.9526	1871	0.4877	0.6077	0.1150	0.9363
Min; Latent	301	2149	0.6916	0.8149	0.0845	0.9736	1956	0.6431	0.7480	0.0783	0.9694
Min; Latent; Examiner	470	770	0.8570	0.9233	0.0303	0.9946	1596	0.8577	0.9099	0.0271	0.9954
Min; MedianMin	2	1950	0.5979	0.7424	0.1080	0.9531	1812	0.5039	0.6233	0.1099	0.9406
Min; OC	2	1974	0.5930	0.7384	0.1064	0.9527	1846	0.4945	0.6144	0.1102	0.9382
Min; OC(MC)	2	1959	0.5961	0.7409	0.1054	0.9529	1844	0.4951	0.6149	0.1110	0.9383
Min; PtStd	2	2012	0.5852	0.7319	0.1126	0.9502	1854	0.4922	0.6121	0.1107	0.9380

Table S8: Logistic regression models describing associations between latent annotation and value determinations made by the same examiner (n=3730). These models describe associations between examiners' annotation and determination responses to the latents. Dozens of additional variants of these models were fitted and generally yielded similar results.

1 Ulery B, Hicklin R, Kiebuzinski G, Roberts M, Buscaglia J (2013) Understanding the sufficiency of information for latent fingerprint value determinations. *Forensic Sci Int* **230(1)**:99-106. (http://www.noblis.org/media/3c760709-5971-4efe-8edf-f00435fcdf1b/docs/article_understanding_sufficiency_information_latent_pdf)

2 Ulery BT, Hicklin RA, Buscaglia J, Roberts MA (2011) Accuracy and reliability of forensic latent fingerprint decisions. Proc Natl Acad Sci USA 108(19): 7733-7738. (http://www.pnas.org/content/108/19/7733.full.pdf)
