# Supplementary material for: Measuring What Latent Fingerprint Examiners Consider Sufficient Information for Individualization Determinations
Source: PLoS One. 2014 Nov 5;9(11):e110179. doi: 10.1371/journal.pone.0110179 (PMC4221158; doi:10.1371/journal.pone.0110179)
Supplement: Appendix S12 — Models of sufficiency for individualization. (PDF) [file pone.0110179.s012.pdf]

Appendix SI-12 Models of sufficiency for individualization

The following tables evaluate logistic regression models describing associations between examiner markup and examiner determinations {Individualization, Insufficient}; insufficient includes both inconclusive and no value determinations. Each model was fit to the 2671 valid responses on mated pairs (which omit 125 false exclusions). In these models, all terms are additive; asterisks denote cross-product terms expressing interactions between pairs of explanatory variables.

The base rate in these models is the percentage of the 2671 mated pairs that were ***not*** individualized (1018 were NV or inconclusive); i.e., a trivial model that assumes examiners will always individualize mated pairs would have a 38.1% misclassification rate.

Statistical measures such as AICc and Generalized R^2 unrealistically assume that responses are independent. Each examiner and image contributed to multiple responses. Consequently, these statistics may be substantially biased (and indicate the models are better than they really are). Such biases were considered when selecting models for inclusion in the tables.

**ImagePair**	Nominal variable identifying the mated pair (n=231)
**Examiner**	Nominal variable identifying the examiner (n=165)
**A1, A2, A3, A4, A5**	Area of red, yellow, green, blue, aqua from this examiner's corresponding clarity map
**any_CDMP**	Number of corresponding and debatably corresponding features marked by this examiner (Cores, Deltas, Minutiae, and Points)
**any_CMin**	Number of corresponding and debatably corresponding minutiae marked by this examiner(=CMin+deb_CMin)
**CD_rate**	Continuous voted metric indicating presence of core or delta (107 voting examiners)
**CDMP**	Number of corresponding features marked by this examiner (Cores, Deltas, Minutiae, and Points)
**CMin**	Number of corresponding minutiae marked by this examiner (Analysis phase NV responses were counted as having 0 corresponding minutiae)
**CMin0, …, CMin5**	Number of corresponding minutiae marked in black, …, aqua clarity regions
**complexity**	Determined during preliminary screening, based on distortion, background or processing
**deb_CMin**	Debatably corresponding minutiae as marked by this examiner
**deb_CMin_green**	Debatably corresponding minutiae as marked by this examiner annotated as green (or higher) clarity
**deb_CMin_yellow**	Debatably corresponding minutiae as marked by this examiner annotated as yellow (or lower) clarity
**CMin_green**	Corresponding minutiae that this examiner annotated as green (or higher) clarity (=CMin3 + CMin4 + CMin5)
**CMin_yellow**	Corresponding minutiae that this examiner annotated as yellow (or lower) clarity (=CMin0 + CMin1 + CMin2)
**Has_CC_map**	examiner marked at least 3 corresponding or debatable features
**Marked_CD**	Nominal metric indicating whether this examiner marked any cores or deltas
**Marked_CMin**	Whether any corresponding minutiae were marked (nominal variable)
**Mean(CMin)**	Mean(corresponding minutiae) among all examiners
**Median(CMin)**	Median(corresponding minutiae) among all examiners
**MedianOverallClarity**	Overall Clarity from median clarity map (calculated among all examiners)
**OverallClarity**	Overall Clarity from this examiner's corresponding clarity map (compare to MedianOverallClarity)
**otherPts**	Number of corresponding "other" features (other than minutiae, cores, and deltas) that were marked by this examiner
**PtStd**	whether examiner followed a 12-point standard
**VCMP**	latent rated VEO or VID
**VID**	latent rated VID

Table S9: Legend of predictor terms

In the following tables, DF = degrees of freedom; R^2 = entropy R^2; AICc = corrected Akaike Information Criterion; Gen R^2 = Generalized R^2; Misclass = misclassification rate; AUC = area under the (receiver operating characteristic) curve.

Predictors	DF	AICc	R^2	Gen R^2	Misclass	AUC
None (base rate)	0	3552	0.0000	0.0000	0.3811	0.5000
ImagePair	230	3295	0.5854	0.7354	0.1303	0.9458
Examiner	164	3546	0.1004	0.1699	0.3280	0.7024
ImagePair; Examiner	394	1696	0.7837	0.8801	0.0629	0.9865
complexity	1	3518	0.0103	0.0185	0.3811	0.5600
Median(Difficulty)	1	3407	0.0414	0.0728	0.2493	0.5225
CD_rate	1	3053	0.1413	0.2328	0.3164	0.7399
Median clarity A1; A2; A3; A4; A5	5	2764	0.2250	0.3515	0.2396	0.8179
CD_rate; MedianOverallClarity	2	2479	0.3033	0.4513	0.2314	0.8457
Median(CMin)	1	1806	0.4925	0.6533	0.1355	0.9219
Median(CMin); CD_rate	2	1808	0.4925	0.6533	0.1355	0.9216
Median(CMin); MedianOverallClarity	2	1786	0.4985	0.6589	0.1359	0.9252
Mean(CMin)	1	1751	0.5079	0.6676	0.1449	0.9298
Median(CMin); Examiner	165	1577	0.6555	0.7909	0.0947	0.9657
Median(CMin); Examiner; (Median(CMin) * Examiner)	329	1700	0.7333	0.8468	0.0940	0.9787

Table S10: Individualization determination {Individualization, Insufficient} as a dependent response to (A) image pairs and examiners; (B) attributes of the image pairs as estimated by median statistics (n=2671). These models are intended to address questions of causality and therefore do not include same-examiner associations between the predictor variables and the determinations. Predictors such as Median(CMin) do not vary by image and therefore describe attributes of the images themselves (albeit based on examiners' responses to the image).

Fig. S18 reveals the goodness of fit of the *Median(CMin)* model from Table S10.

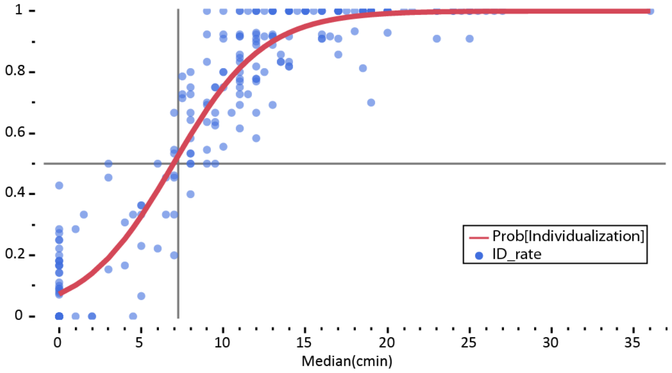

Fig. S18: Overlay plot showing the fitted logistic model for Median(CMin) against a scatterplot of the individualization rates for 231 mated image pairs. The cross-hairs indicate how the misclassification rate is calculated based on whether the probability estimate from the fitted model exceeds 50%.

Predictors	DF	AICc	R^2	Gen R^2	Misclass	AUC
None (base rate)	0	3552	0.0000	0.0000	0.3811	0.5000
Marked_CD	1	3312	0.0684	0.1181	0.3811	0.6245
VCMP	1	2477	0.3034	0.4513	0.2055	0.7304
A1; A2; A3; A4; A5	5	2184	0.3883	0.5483	0.1617	0.8977
Difficulty	5	2148	0.3983	0.5590	0.2014	0.8643
A1; A2; A3; A4; A5; CD_rate; complexity	7	2081	0.4184	0.5802	0.1569	0.9063
Has_CC_map	1	1992	0.4401	0.6023	0.1355	0.8252
OverallClarity	1	1976	0.4446	0.6068	0.1711	0.9092
Marked_CMin	1	1834	0.4846	0.6458	0.1262	0.8349
OverallClarity; Has_CC_map	2	1796	0.4959	0.6564	0.1382	0.9092
Has_CC_map; A1; A2; A3; A4; A5	6	1798	0.4976	0.6581	0.1359	0.9060
VID	1	1596	0.5517	0.7068	0.1041	0.8635
any_CDMP	1	1092	0.6936	0.8191	0.0786	0.9695
any_CMin	1	1084	0.6959	0.8207	0.0738	0.9698
CDMP	1	870	0.7560	0.8621	0.0592	0.9803
cdm; otherPts	2	872	0.7561	0.8621	0.0592	0.9804
CMin	1	862	0.7584	0.8637	0.0595	0.9806
CMin; CD	2	864	0.7584	0.8637	0.0595	0.9807
CD_rate; CMin	2	862	0.7590	0.8641	0.0577	0.9813
CMin; OverallClarity	2	855	0.7609	0.8654	0.0580	0.9811
CMin0; CMin1; CMin2; CMin3; CMin4; CMin5	6	858	0.7623	0.8663	0.0573	0.9817
CMin; PtStd	2	848	0.7628	0.8665	0.0573	0.9812
CMin; A1; A2; A3; A4; A5	6	839	0.7677	0.8698	0.0577	0.9821
CMin_green; CMin_yellow	2	854	0.7611	0.8655	0.0595	0.9814
CMin_green; CMin_yellow; (CMin_green*CMin_yellow)	3	851	0.7625	0.8664	0.0547	0.9812
CMin_green; CMin_yellow; deb_CMin_green; deb_CMin_yellow	4	857	0.7614	0.8657	0.0562	0.9813
CMin_green; CMin_yellow; g_point; y_point	4	846	0.7647	0.8678	0.0588	0.9820
CMin_green; CMin_yellow *[from median clarity map]* §	2	825	0.7693	0.8708	0.0539	0.9825
CMin0; CMin1; CMin2; CMin3; CMin4; CMin5 *[from median clarity map]* §	6	829	0.7705	0.8716	0.0539	0.9828
CMin; Difficulty (ordinal)	6	799	0.7790	0.8771	0.0539	0.9842
CMin; examiner	165	827	0.8668	0.9303	0.0296	0.9943

§ *These two models use the median clarity map (generated across multiple examiners) to describe the clarity of the images where this examiner marked the minutiae.*

Table S11: Logistic regression models describing associations between annotations and determinations {Individualization, Insufficient} made by the same examiner (n=2671). These models describe associations between examiners' annotation and determination responses to the image pairs. Dozens of additional variants of these models were fitted and generally yielded similar results. Such models included alternate measures of features and clarity (such as largest contiguous areas at each level of clarity), cross terms and transforms of terms.
