# Supplementary material for: Measuring What Latent Fingerprint Examiners Consider Sufficient Information for Individualization Determinations
Source: PLoS One. 2014 Nov 5;9(11):e110179. doi: 10.1371/journal.pone.0110179 (PMC4221158; doi:10.1371/journal.pone.0110179)
Supplement: Appendix S13 — Repeatability of minutia counts (Analysis phase). (PDF) [file pone.0110179.s013.pdf]

Appendix SI-13 Repeatability of minutia counts (Analysis phase)

Differences in minutia counts understate the variability among examiners: annotations may have similar minutia counts but differ greatly in which specific minutiae were marked. Some of the variability in minutia selection may be due to the examiners themselves not being consistent in their minutia selection. In this study, there were 13 instances in which latents were presented twice to the same examiner, once in a mated pair, once in a nonmated pair (Table S4). The examiners were permitted to review their previous work, so they could have compared their responses if they noticed the duplication during the Analysis phase. A manual review of these annotations was conducted to determine whether the same features were marked in the two presentations. One latent (#1) was presented to two different examiners (twice each) and neither examiner marked any features. However, for none of the other 11 latents did the examiner mark the same set of features both times, and value determinations differed in three cases. This small sample of data suggests that much of the lack of interexaminer reproducibility of minutiae could be explained by a lack of intraexaminer repeatability; this echoes our previous findings with examiners' determinations [1].

Latent	Value A	Features A	Value B	Features B	Features in common
1	NV	0	NV	0	-
1	NV	0	NV	0	-
2	NV	2+D	VEO	3+D	2+D
3	VID	9	VID	9 + C	8
4	VID	7+D	VID	8+D	6+D
5	VID	7+C+D	VID	8+C+D	5+C+D
6	VID	11	VID	11+C+D	10
7	VID	11	VID	11	8
8	VID	21	VID	22+C	17
9	VID	7+C+D	VEO	7+D	4+D
10	VID	10+1 other	VID	15	8
11	VID	11+C	VEO	4	4
12	VID	9	VID	12+5 others	8

Table S4: Analysis phase responses for 13 latents that were presented twice to the same examiner. "A" and "B" in the column headers refer to the two presentations of each print. "Features" columns indicate minutiae (total count), cores (C), deltas (D), and "other" points marked. The column "Features in common" counts the number of features marked in both presentation A and presentation B (including several cases where the placement shifted to an adjacent ridge).

1 Ulery BT, Hicklin RA, Buscaglia J, Roberts MA (2012), Repeatability and Reproducibility of Decisions by Latent Fingerprint Examiners. PLoS ONE 7:3. (http://www.plosone.org/article/info:doi/10.1371/journal.pone.0032800)
