# Supplementary material for: Measuring What Latent Fingerprint Examiners Consider Sufficient Information for Individualization Determinations
Source: PLoS One. 2014 Nov 5;9(11):e110179. doi: 10.1371/journal.pone.0110179 (PMC4221158; doi:10.1371/journal.pone.0110179)
Supplement: Appendix S14 — Reproducibility of determinations by median corresponding minutia count. (PDF) [file pone.0110179.s014.pdf]

Appendix SI-14 Reproducibility of determinations by median corresponding minutia count

In the Black Box study [1,2], we described reproducibility of determinations as overall averages for large datasets. In this study, we are able to refine our description of reproducibility as a function of examiner descriptions of specific images. We use percentage agreement to describe reproducibility of determinations. This commonly used statistic simply describes the proportion of times paired responses (different examiners on the same image pair) are in agreement. Percentage agreement on the $\boldsymbol{i}^{\text{th}}$ image pair is defined as

$$P_i = \frac{1}{n(n-1)} \sum_{j=1}^{k} n_{ij}(n_{ij} - 1)$$

where $\boldsymbol{n}$ is the number of examiners assigned the image pair, $\boldsymbol{k}$ is the number of determination categories, and $\boldsymbol{n_{ij}}$ is the number of responses assigning the $\boldsymbol{i}^{\text{th}}$ image pair to the $\boldsymbol{j}^{\text{th}}$ determination category. k=2 for agreement on {Individualization, Not Individualization} or k=3 for {Individualization, Exclusion, Inconclusive or NV}. For 3-way agreement, percentage agreement implicitly treats all disagreements as being equally serious. So, for example, the disagreement “individualization vs. exclusion” is not weighted differently than the disagreement “individualization vs. inconclusive.”

Fig. S12 and Table S3 show the percentage agreement among examiners on mated comparison conclusions as a function of the median number of corresponding minutiae marked by all examiners. Percentage agreement was approximately 50% when the median was near 7 to 9 corresponding minutiae, and was below 90% for all categories except when the median was above 16 minutiae.

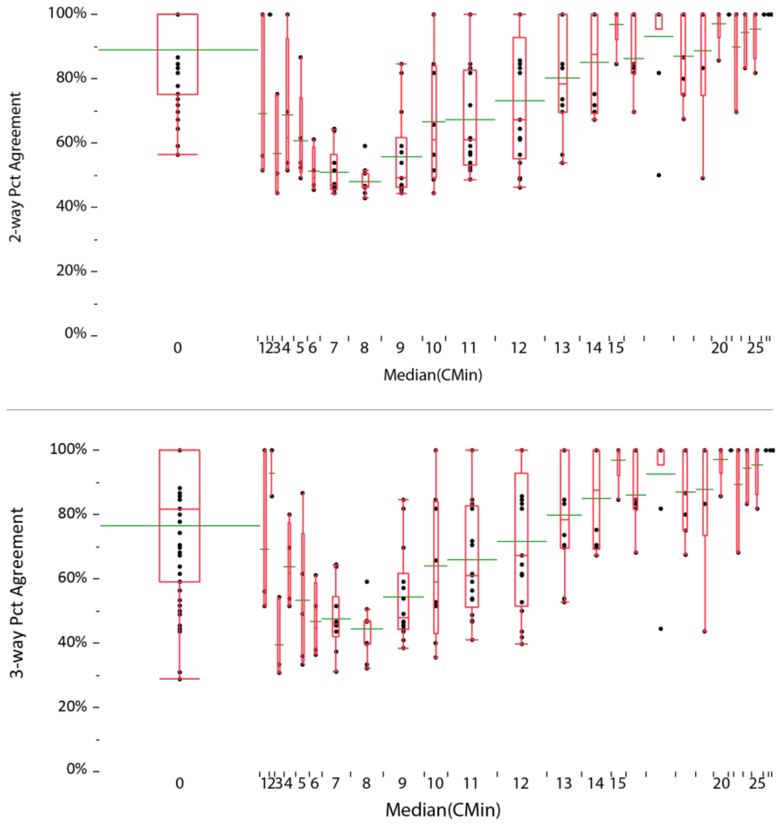

Fig. S12: Percentage agreement by median corresponding minutia count (n=2796 responses by 165 examiners to 231 mated image pairs). Fractional medians are rounded down.

Median(CMin)	N	Mean 2-way Pct Agreement	Mean 3-way Pct Agreement
0	55	89.0%	76.5%
1-3.5	8	72.2%	64.0%
4-6.5	13	60.3%	54.5%
7-9.5	35	51.9%	49.3%
10-12.5	42	69.5%	67.9%
13-15.5	27	85.1%	84.9%
16+	51	92.1%	91.8%
Overall	231	77.9%	73.5%

Table S3: Percentage agreement by median corresponding minutia count (n=2796 responses by 165 examiners to 231 mated image pairs).

1 Ulery BT, Hicklin RA, Buscaglia J, Roberts MA (2011) Accuracy and reliability of forensic latent fingerprint decisions. Proc Natl Acad Sci USA 108(19): 7733-7738. (http://www.pnas.org/content/108/19/7733.full.pdf)

2 Ulery BT, Hicklin RA, Buscaglia J, Roberts MA (2012), Repeatability and Reproducibility of Decisions by Latent Fingerprint Examiners. PLoS ONE 7:3. (http://www.plosone.org/article/info:doi/10.1371/journal.pone.0032800)
