# Supplementary material for: Measuring What Latent Fingerprint Examiners Consider Sufficient Information for Individualization Determinations
Source: PLoS One. 2014 Nov 5;9(11):e110179. doi: 10.1371/journal.pone.0110179 (PMC4221158; doi:10.1371/journal.pone.0110179)
Supplement: Appendix S15 — Reproducibility of responses. (PDF) [file pone.0110179.s015.pdf]

Appendix SI-15 Reproducibility of responses

Fig. S6 shows associations between examiners' markup and their determinations, and also interexaminer variability from the Analysis phase. These patterns are similar to those from the Comparison/Evaluation phase shown in Figure 5. Counts of zero minutiae are much more common in the corresponding minutia counts shown in Figure 5 than in the latent print minutia counts shown in Fig. S6 in part because Figure 5 counts image pairs whose latents were determined NV and not compared as having zero corresponding minutiae. For mated pairs, zero corresponding minutiae are likely to be marked for inconclusive determinations if the examiner fails to see the proper alignment of the latent to the exemplar.

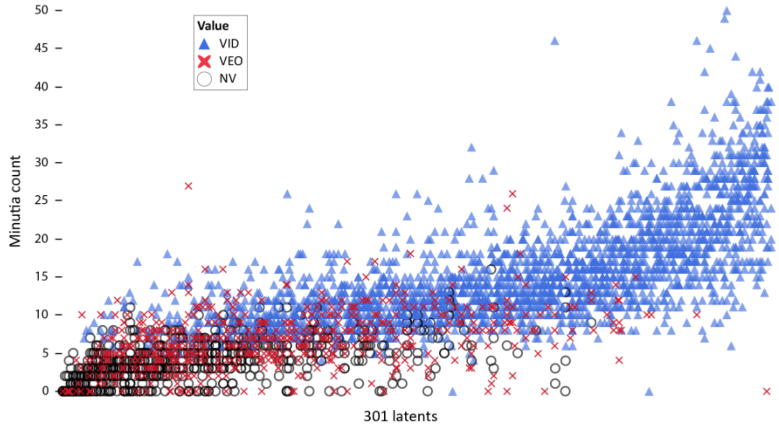

Fig. S6: Individual examiner latent print minutia counts (y-axis) and value determinations (color) by latent print (x-axis, sorted by mean minutia count); n=3730 responses by 170 examiners to 301 latents; 11 points representing minutia counts > 50 not shown (10 of which were for a single latent).

Fig. S7 is similar to Figure 5, but the x-axis is scaled to show how the mean number of corresponding minutiae relates to comparison determinations.

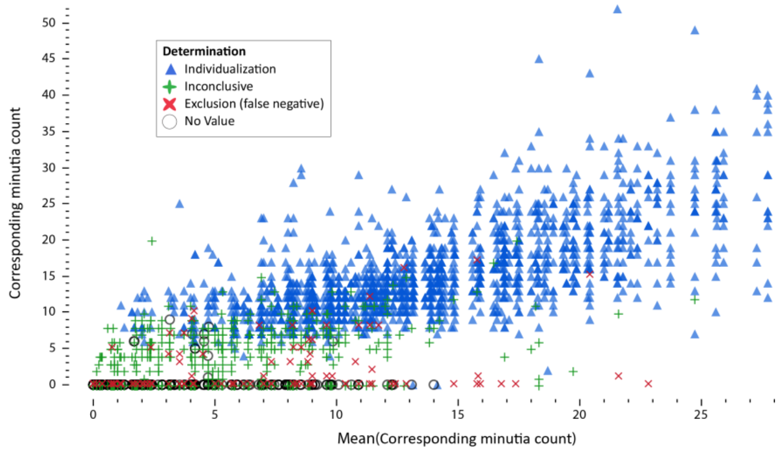

Fig. S7: Corresponding minutia counts (y-axis) and determinations (color) by mean corresponding minutia count (x-axis); n=2796 responses by 165 examiners to 231 mated image pairs. This chart plots the same data as Figure 5, but by mean count instead of by image pair.

Fig. S8, differs from Figure 5 by plotting NV determinations using the number of minutiae marked in the latent; Figure 5 represents those image pairs as having zero corresponding minutiae.

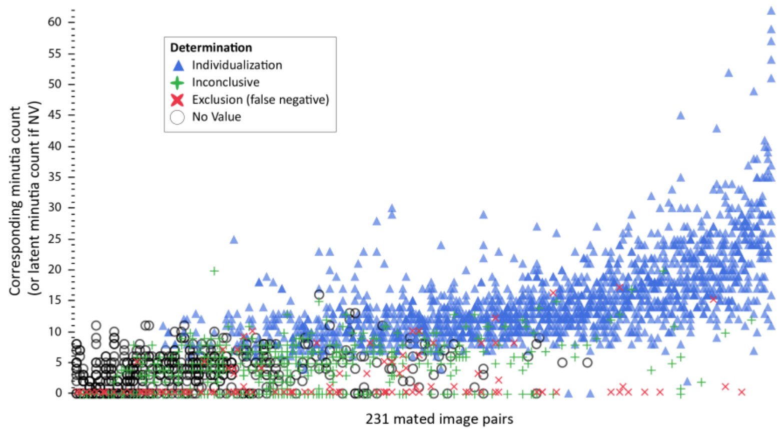

Fig. S8: Corresponding minutia counts (y-axis) and comparison determinations (color) by image pair (x-axis, sorted by mean corresponding minutia count); n=2796 responses by 165 examiners to 231 mated image pairs. This chart plots the same data as Figure 5, except that instead of reporting zero corresponding minutiae for NV determinations, the latent minutia count is reported.

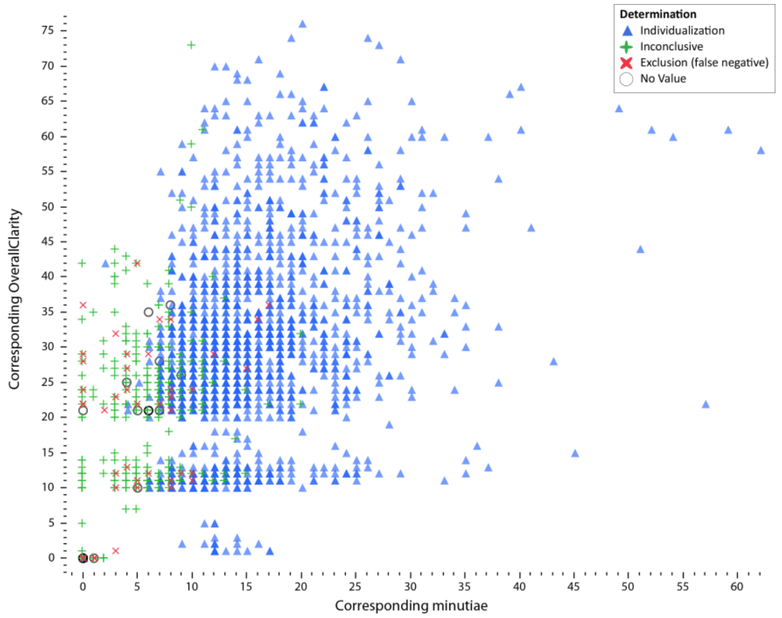

Fig. S9: Examiner determinations in relation to their corresponding minutia counts and OverallClarity as measured on the corresponding clarity map (n=2796 responses by 165 examiners on 231 mated pairs).

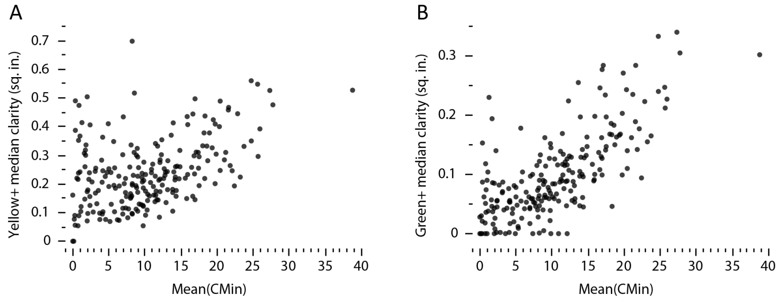

Fig. S10: Associations between corresponding minutia counts and clarity on 231 mated image pairs. Median corresponding minutia count (x-axis) by area in square inches on the median clarity maps that were marked (A) yellow or higher clarity, and (B) green or higher clarity (y-axis).
