# Supplementary material for: Measuring What Latent Fingerprint Examiners Consider Sufficient Information for Individualization Determinations
Source: PLoS One. 2014 Nov 5;9(11):e110179. doi: 10.1371/journal.pone.0110179 (PMC4221158; doi:10.1371/journal.pone.0110179)
Supplement: Appendix S16 — Corresponding minutia counts and determinations for nonmated image pairs. (PDF) [file pone.0110179.s016.pdf]

Appendix SI-16 Corresponding minutia counts and determinations for nonmated image pairs

Fig. S11 shows the corresponding minutia counts associated with determinations and their reproducibility among examiners for nonmated image pairs; see Figure 5 for the equivalent chart for mated image pairs. Among the nonmated image pairs, 89% of the annotations had no corresponding minutiae marked, and few had more than seven corresponding minutiae marked. The single erroneous individualization (false positive) had 14 corresponding minutiae marked (the highest count among 582 comparisons of nonmated pairs).

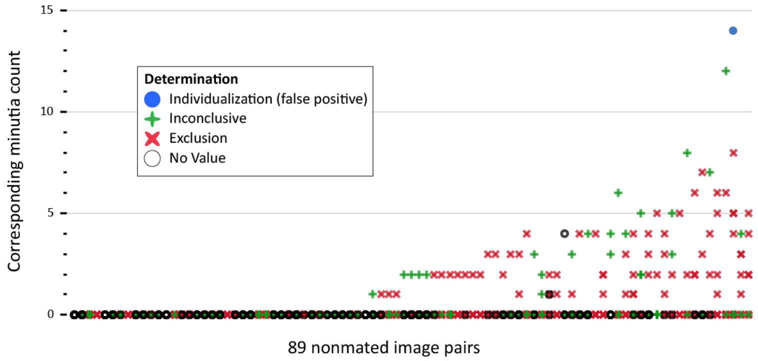

Fig. S11: Corresponding minutia count (y-axis) and determination (color) by nonmated image pair (x-axis). X-axis is sorted by median, then by mean corresponding minutia count. (n=847 responses by 165 examiners to 89 nonmated image pairs, mean of 9.5 responses per image pair; one invalid response omitted)
