# Supplementary material for: Measuring What Latent Fingerprint Examiners Consider Sufficient Information for Individualization Determinations
Source: PLoS One. 2014 Nov 5;9(11):e110179. doi: 10.1371/journal.pone.0110179 (PMC4221158; doi:10.1371/journal.pone.0110179)
Supplement: Appendix S17 — Estimating repeatability of determinations. (PDF) [file pone.0110179.s017.pdf]

Appendix SI-17 Estimating repeatability of determinations

In [1] we measured interexaminer percentage agreement on mated pairs to be 86.6% and intraexaminer percentage agreement to be 92.2%. We also observed a proportional relation between repeatability and reproducibility. If we assume a similar relation holds here and that these numbers are driven by the proportion of borderline sufficiency image pairs in the test, then we can estimate a repeatability rate for this study. Based on the observed interexaminer percentage agreement of 79.5% in this study, this simple model predicts an intraexaminer percentage agreement of 88%, which would correspond to a misclassification rate of about 6% (i.e., approximately (1-0.88)/2).

In Table 5, we showed that the *Median(CMin) + Examiner* model resulted in a misclassification rate of 9.5%, most of which can be attributed to the lack of repeatability of individualization determinations.

1 Ulery BT, Hicklin RA, Buscaglia J, Roberts MA (2012), Repeatability and Reproducibility of Decisions by Latent Fingerprint Examiners. *PLoS ONE* **7**:3. (http://www.plosone.org/article/info:doi/10.1371/journal.pone.0032800)
