# Supplementary material for: Measuring What Latent Fingerprint Examiners Consider Sufficient Information for Individualization Determinations
Source: PLoS One. 2014 Nov 5;9(11):e110179. doi: 10.1371/journal.pone.0110179 (PMC4221158; doi:10.1371/journal.pone.0110179)
Supplement: Appendix S18 — Effect of point standard. (PDF) [file pone.0110179.s018.pdf]

Appendix SI-18 Effect of point standard

In the participant survey (Appendix SI-7, question #20), 19 participants self-identified as having point standards. The free-text responses were sometimes ambiguous. Four examiners specified a seven or eight point standard; given that nearly all of the individualizations had at least seven corresponding minutiae, such a standard presumably would have had negligible influence on the results of this test. Six of the examiners who specified a point count did not conform to that standard in the test, individualizing two or more times with fewer total features annotated. Therefore, for the purposes of analysis, ten participants were identified as having and following a standard: all ten had 12-point standards; most of these were from a single country, none from the U.S..

As was noted in Figure 8, one effect of a point standard is that it has a substantial effect on the number of minutia marked. For the examiners with a 12-point standard, Fig. S13B shows the strikingly disproportionate number of latents with 12 minutiae marked in Analysis. For the examiners without point count standards, we saw an abrupt step at seven corresponding minutiae in Figure 8A; a similar step is not clearly evident in Fig. S13A.

A:

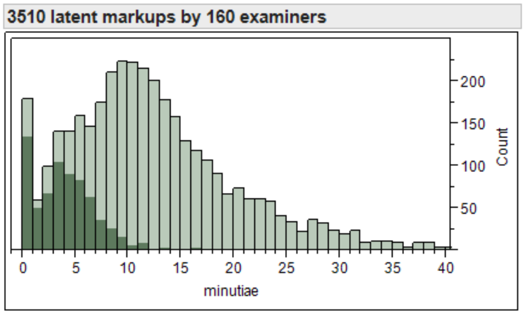

B:

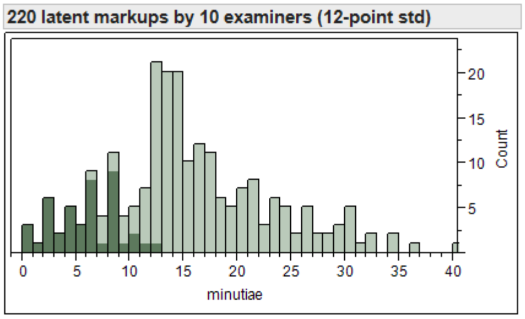

Fig. S13: Effect of point count standards on minutia count. (A) Analysis phase minutia counts by 160 examiners without point count standards; (B) Analysis phase minutia counts by 10 examiners who followed a 12-point standard. NV determinations shaded; plots truncated at 40 minutiae.
