# Supplementary material for: Measuring What Latent Fingerprint Examiners Consider Sufficient Information for Individualization Determinations
Source: PLoS One. 2014 Nov 5;9(11):e110179. doi: 10.1371/journal.pone.0110179 (PMC4221158; doi:10.1371/journal.pone.0110179)
Supplement: Appendix S19 — Minimum number of corresponding minutiae. (PDF) [file pone.0110179.s019.pdf]

Appendix SI-19 Minimum number of corresponding minutiae

The minimum number of corresponding minutiae that each examiner reported when individualizing varied across examiners as shown in Fig. S14. We investigated the reasons for this wide variation in the data and what it tells us about meaningful differences among examiners as opposed to artifacts of how the data was collected. Specifically, we investigated four sources of variation in the data:

- outliers that are not indicative of extreme decision criteria (discussed in Appendix SI-9);
- random variations due to small sample sizes;
- variations associated with differing individualization rates from examiner to examiner; and
- variations associated with subjective differences in marking minutiae.

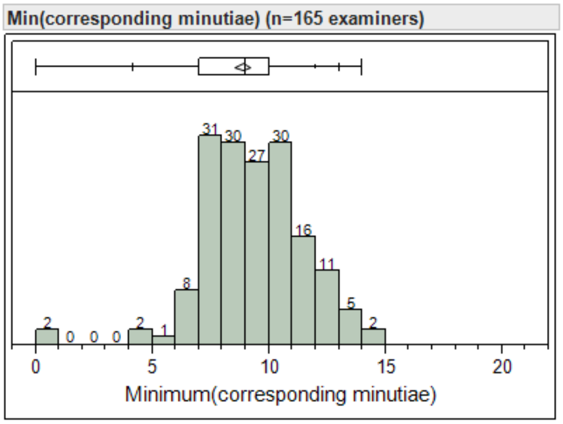

Fig. S14: Distribution of Minimum(corresponding minutiae) marked by examiners when they individualized (n=165).

In order to understand the substantial dispersion in Fig. S14, we performed three simulations to isolate contributing factors.

The minimum is an extreme statistic and biased upwards: if each examiner had been assigned many more comparisons, the minimum count for some examiners would have been lower. In the first simulation (Fig. S15), we assume there are no examiner differences in the number of corresponding minutiae reported, but that there are real differences in the numbers of individualizations per examiner. That is, the simulated examiners differ in sample sizes (up to 17 individualizations per examiner) but not in reported minutia counts. This simulation demonstrates that most of the observed variation is a random effect associated with the small sample sizes. Each simulation run shows nearly as much dispersion as the actual data even though the counts were randomly assigned to simulated examiners. We conclude that most of the dispersion in Fig. S14 is a consequence of the limited number of measurements obtained per examiner.

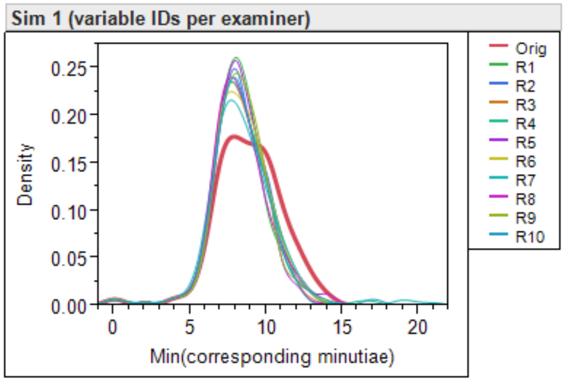

Fig. S15: Simulation #1: variable number of individualizations per examiner. Ten simulation runs in which the actual corresponding minutia counts were randomly assigned to 165 simulated examiners; the number of individualizations per simulated examiner matches the actual distribution of individualizations per examiner. In each run, the random reassignments were performed by permuting the original counts of the 1653 individualizations. The (bold) reference line shows the actual distribution of Minimum(corresponding minutiae). Data are summarized as smoothed kernel density estimates. This simulation demonstrates that most of the observed interexaminer variation in minimum number of corresponding minutiae is a random effect associated with the small sample size.

Next we investigated how much the variable number of individualizations per examiner contributed to the dispersion. In this simulation (Fig. S16), we assume there are no examiner differences in the number of corresponding minutiae reported or in individualization rates. Comparing Fig. S15 and Fig. S16, we see that the varying number of individualizations per simulated examiner had a very minor effect on the distribution. As demonstrated via the logistic regression models (Table 5A), there are important real differences among examiners' individualization rates (more than can be explained by the random test assignments). Fig. S16 demonstrates that these real differences in individualization rates, although they have great effect on the sample size from which the extreme statistic, minimum(corresponding minutiae), was calculated, contribute very little to the dispersion in Fig. S14.

The first simulation (Fig. S15) shows that the actual sample distribution differs substantially from the simulations. The second simulation (Fig. S16) shows that these differences are not due to differences in individualization rates. This means that there are real examiner differences in the number of corresponding minutiae that examiners mark when individualizing. Some examiners will not make individualization determinations if they only mark 7 or 8 corresponding minutiae.

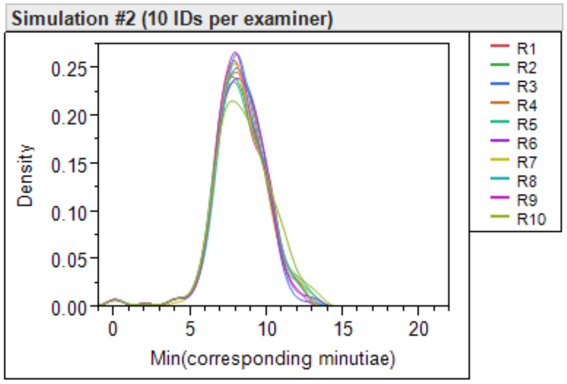

Fig. S16: Simulation #2: constant number of individualizations per examiner. Ten simulation runs in which the actual corresponding minutia counts were randomly assigned to 165 simulated examiners; each simulated examiner made 10 individualizations. In each run, the random reassignments were performed by permuting the original counts of the 1653 individualizations. The random reassignments were performed by permuting the original set of 1653 actual counts (3 high counts were omitted to reduce the number to 1650 = 10 * 165 and because omitting high counts would not affect the minima). The similarity of this outcome to the previous simulation demonstrates that real differences in examiners' individualization rates contribute little to the measured dispersion.

The final simulation (Fig. S17) investigates whether the real differences among examiners revealed in Fig. S15 pertain more to differences in how many minutiae they marked or to which image pairs they individualized. In order to perform this analysis, we remove subjectivity in the reported counts by measuring a fixed statistic for each image pair, mean(corresponding minutiae). The results show only subtle differences between the actual distribution of minimum(mean(corresponding minutiae)) and the simulated distribution. In other words, the real examiner effect on minimum(corresponding minutiae) relates largely to differences in how the examiners count minutiae, not to differences in the images.

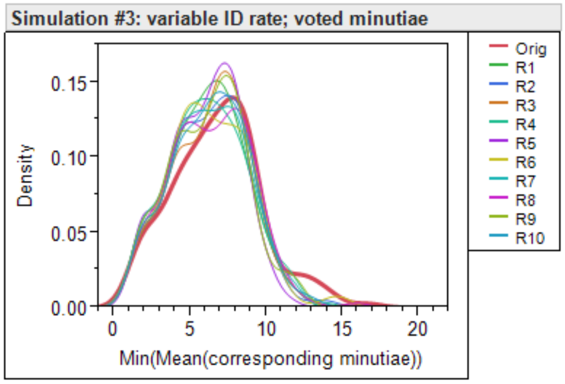

Fig. S17: Simulation #3: variable number of individualizations per examiner, fixed minutia count for each image pair. Ten simulation runs in which the actual mean(corresponding minutia) counts were randomly assigned to 165 simulated examiners; the number of individualizations per simulated examiner matches the actual distribution of individualizations per examiner. In each run, the random reassignments were performed by permuting the original mean counts of the 1653 individualizations. The (bold) reference line shows the actual distribution of Minimum(Mean(corresponding minutiae)). This simulation demonstrates that the real examiner effect on Minimum(corresponding minutiae) relates largely to differences in how the examiners count minutiae, not to differences in the images that they individualized.

In summary, the minimum number of corresponding minutiae required to individualize varied by examiner. More than one-third of examiners individualized with as few as seven or eight minutiae. Most of the observed differences among examiners pertain to the small sample size; that is, more opportunities would have lowered the observed minimum for many examiners. The observed differences in the minimum number of corresponding minutiae relate primarily to differences in the minutia counts that these examiners attributed to the images, not to differences in the images themselves or to differences in the examiners' individualization rates. Examiners do differ substantially in their individualization rates, but differences in their minimum minutia counts do not appear to be an important factor contributing to differing individualization rates.
