# Supplementary material for: Measuring What Latent Fingerprint Examiners Consider Sufficient Information for Individualization Determinations
Source: PLoS One. 2014 Nov 5;9(11):e110179. doi: 10.1371/journal.pone.0110179 (PMC4221158; doi:10.1371/journal.pone.0110179)
Supplement: Appendix S20 — White Box Latent Examiner Study — Instructions. (PDF) [file pone.0110179.s020.pdf]

Appendix SI-20 White Box Latent Examiner Study — Instructions

1 Summary of Test Procedure 2
1.1 Steps to be performed ... 2
1.2 Latent analysis ... 3
1.3 Comparison and evaluation ... 5
2 Markup 7
2.1 Ridge quality painting ... 7
2.2 Features ... 9
2.3 Corresponding features ... 11
3 Analysis and Comparison Determinations 13
3.1 Value determinations ... 13
3.2 Comparison determinations ... 14
3.3 Reason for exclusion ... 14
3.4 Difficulty ... 15
3.5 Comments ... 15
4 Image Processing and Markup Controls 15
4.1 Temporary lines ... 16
4.2 Erase ... 16
4.3 Show/Hide markup ... 17
4.4 Tonal reversal ... 17
4.5 Zoom ... 17
4.6 Rotation ... 17
4.7 Grayscale control ... 17
4.8 Display settings ... 17
4.9 Shortcuts ... 18

1 SUMMARY OF TEST PROCEDURE

In the White Box Study, you will be asked to perform twenty-two friction ridge impression examinations. For each examination, you will document your analyses and comparison of the latent and exemplar impressions and record your value and comparison determinations.

This Study is a follow-on to the Black Box Study performed two years ago. In Black Box, we focused on *accuracy* and *reproducibility* of examiners' decisions. In White Box, the focus is on understanding the basis for these determinations. You will be asked to make analysis (value) and comparison determinations for latent-exemplar fingerprint pairs, and to mark the features (quantity and quality) used in making those determinations. The test data will be analyzed to characterize what constitutes sufficient quantity and quality to support a decision.

Anonymity will be assured through the use of examiner IDs and by controlling the flow of information. The analysis data will be kept separate from personally identifiable information (names and email addresses) so that results cannot be associated with specific individuals even by members of the team involved in test administration or analysis. Information associating participants and examiner IDs will be destroyed upon completion of the study. The background survey does not request participants' names, employer, or any other personally identifying information.

Your careful adherence to test guidelines is critical to achieving these objectives: it is important that you apply the same diligence that you use in casework when taking the test.

1.1 Steps to be performed

The White Box test software includes a status window and a comparison tool as shown in Figure 1.

The status window shows the list of examinations to be performed and the status of your work on each. You may work through the examinations in any order. Each examination proceeds through five stages: To Do, Latent in progress (optional), Latent Complete, Comparison in progress (optional), and Comparison complete. To begin or continue an examination, launch the comparison tool by double-clicking on any item in the list.

For each examination, the comparison tool will first present a latent for analysis, then (unless the latent is deemed No Value) the latent and exemplar will be presented together for comparison and evaluation. A copy of your markup and latent value decision will be recorded when you indicate that the latent markup is complete, however, these may be changed during comparison and evaluation.

After all examinations are complete, you will complete a survey and submit your work. Once you have received a confirmation that your work has been received, you will be asked to Remove User Data. The confirmation email will provide instructions on this and how to uninstall the software.

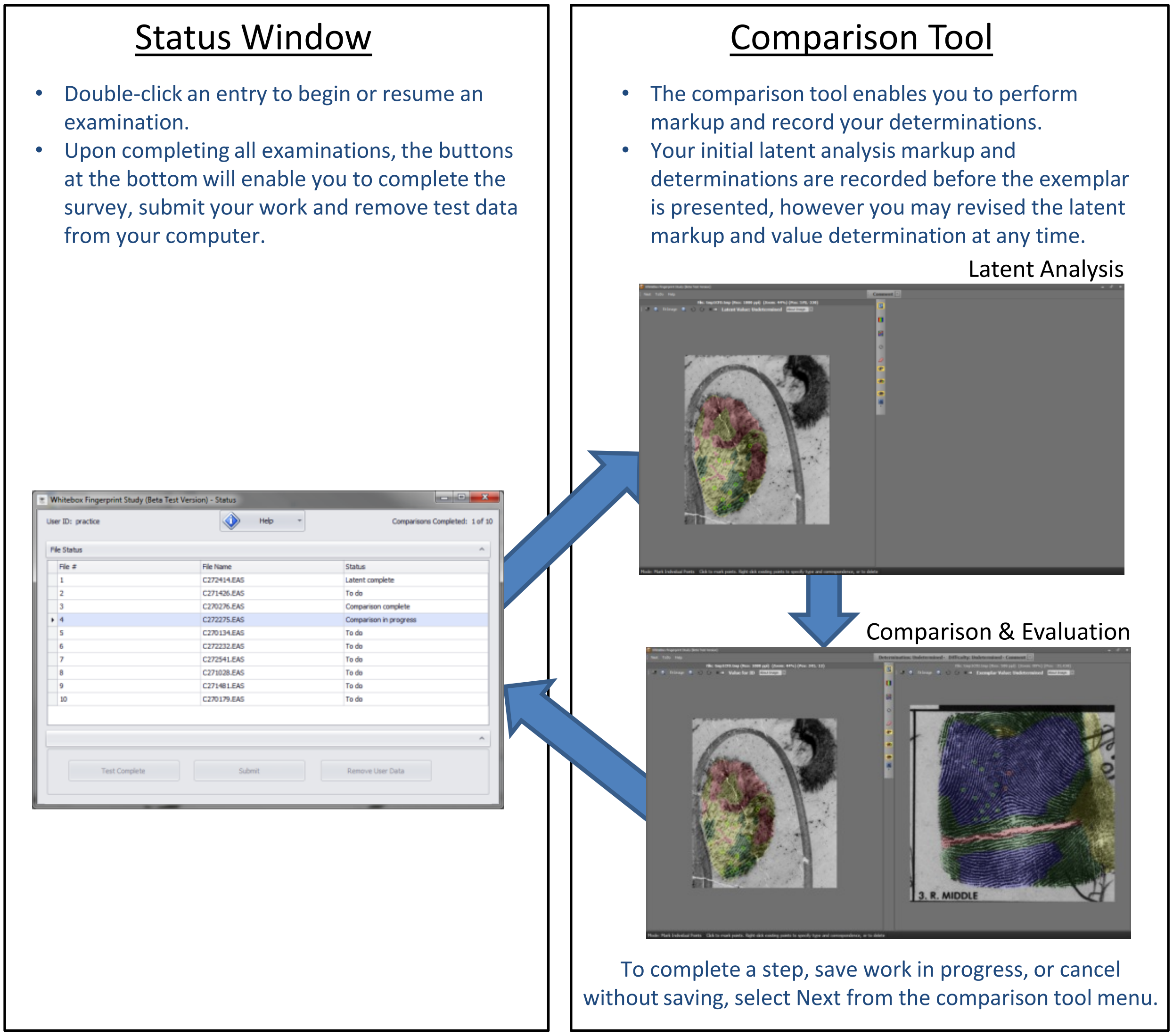

Figure 1: When you launch the White Box application, the status window presents a list of all assigned examinations together with your work status on each. From this window, you will launch the comparison tool to perform each examination. The status window also provides access to help documentation and, at the end of the test, will enable you to complete the survey, submit your work and remove test data from your computer.

1.2 Latent analysis

During latent analysis, you are required to perform the following steps:

- Paint the quality (clarity) of the latent (throughout the entire region of interest).
- Mark all minutiae, cores, and deltas in the latent.
- Record your value determination.

Figure 2 indicates how the comparison tool is used to perform these steps.

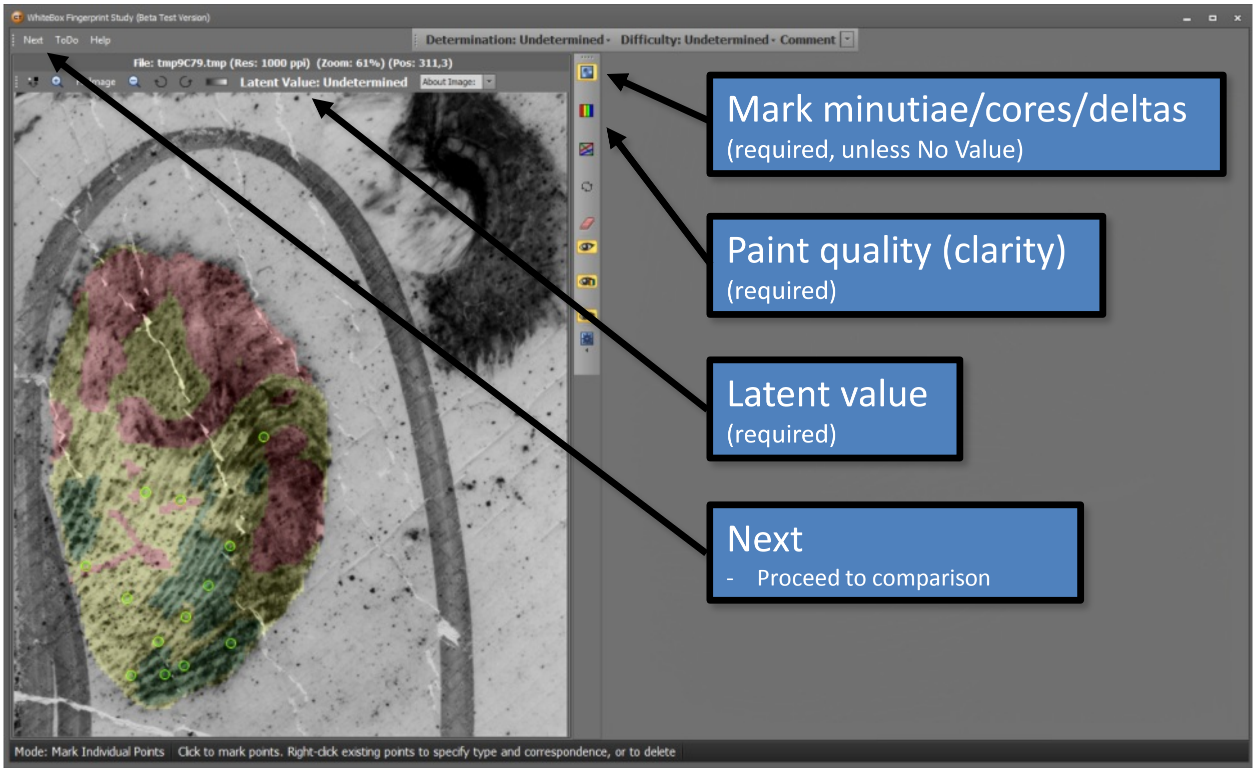

Figure 2: Latent analysis.

After latent analysis, hit Next (or close). You will see a “Close Latent” window (Figure 3) displaying a summary of your markup; warning/errors, if any (such as saying a print is of value without any minutiae marked); and options on saving.

After you have completed latent analysis, you may proceed to comparison and evaluation. Once the exemplar is presented, a copy of your initial latent markup and value determination will be saved for White Box analysis.

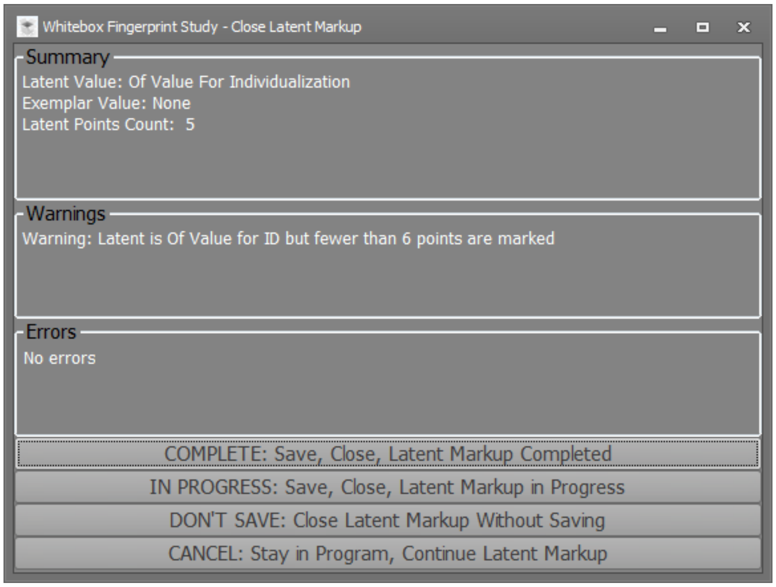

Figure 3: Example of the “Close Latent” window.

1.3 Comparison and evaluation

During comparison and evaluation, you may revise features that you marked during analysis of the latent by adding, deleting or repositioning features, or changing quality markup; you may also change your latent value determination.

During comparison and evaluation, you are required to perform the following steps:

- Paint the quality (clarity) of the exemplar.
- Mark all features in the exemplar that correspond to those marked in the latent.
- Mark any discrepancies used to support an exclusion determination.
- Mark other features whose correspondence (or non-correspondence) you use in making your comparison determination.
- Record your value determination for the exemplar.
- Record your comparison determination.

In the exemplar, you only have to mark correspondences or discrepancies – you do not have to mark every minutia in the exemplar!

Figure 4 indicates how the comparison tool is used to perform these steps.

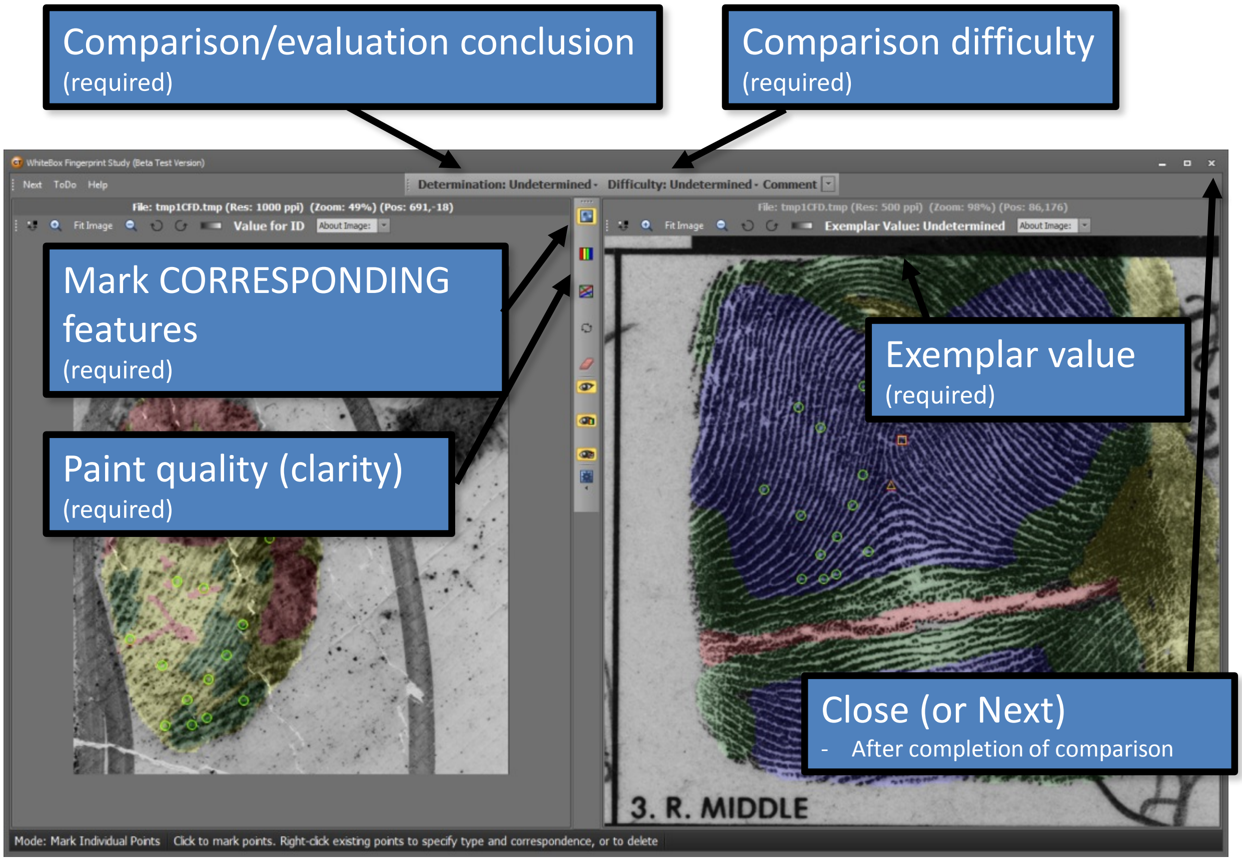

Figure 4: Comparison and evaluation.

When making your determinations, please make the following assumptions:

- Assume that the images provided are the only images available, and that physical evidence, lift cards, fingerprint cards, additional exemplars, and different images of these prints are not available.
- Assume that every impression is a fingerprint, not a palmprint or lower joint.
- For an inconclusive determination, it is assumed that additional exemplars would have been requested and were not available; it is not necessary to state this in a comment.
- Make latent or exemplar value determinations under the assumption that a good-quality exemplar with a large corresponding area may be available for comparison. The specific exemplar shown for comparison may or may not meet these criteria, but that should have no bearing on your value assessment.
- No images have already been claimed "Of Value." In a few images, impressions were marked to indicate which impressions were to be scanned; such marks do not indicate that another examiner necessarily determined that the print is of value.

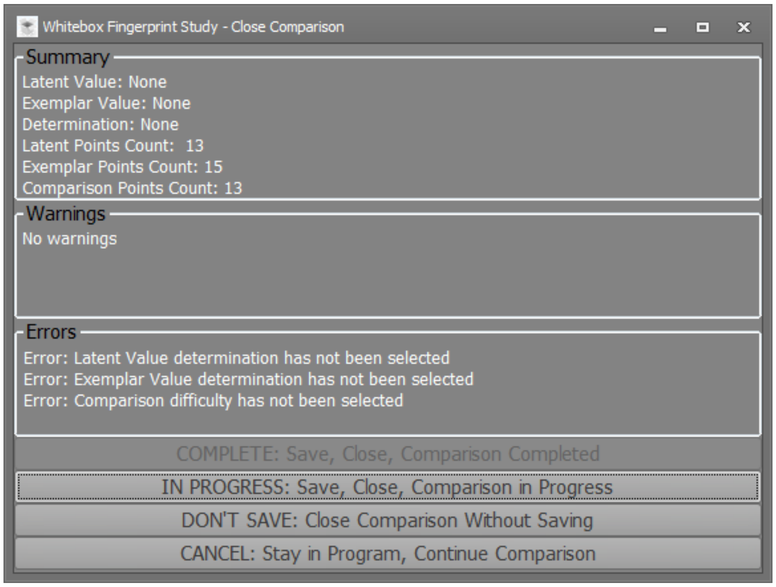

Figure 5: Example of the "Close Comparison" window.

2 MARKUP

Your markup of the fingerprint images is of central interest in this study, which will be looking at the relation between markup and ACE decisions. [1] This markup complies with a subset of features from the Extended Feature Set (EFS), which is the standard for feature markup in the American National Standard ANSI/NIST 2011.

2.1 Ridge quality painting

The ridge quality map is used to document your level of confidence in the marked features. Image quality is documented by painting over the image using standard colors as defined Figure 6. When each image is first presented, the entire image is painted black (denoting background) by default. Paint the all of the impression being evaluated, while leaving the background and other impressions (if any) black. Include all of the impression of interest even when it is superimposed with another impression.

Note particularly two critical distinctions:

- Green (or better) means that you are **certain** of the presence of all of the minutiae you mark in that region AND you are certain that there are no unmarked minutiae.
- Continuous areas marked as yellow or better indicate a single simultaneous impression — any discontinuities (e.g., smears) must be marked in red.

[1] The ridge quality map and markup of features that you will provide on this test correspond to a subset of the Extended Feature Set (EFS), which is part of the ANSI/NIST-ITL standard (fingerprint.nist.gov/standard/). A detailed discussion of EFS markup including examples is available in Markup Instructions for Extended Friction Ridge Features (http://www.noblis.org/interop).

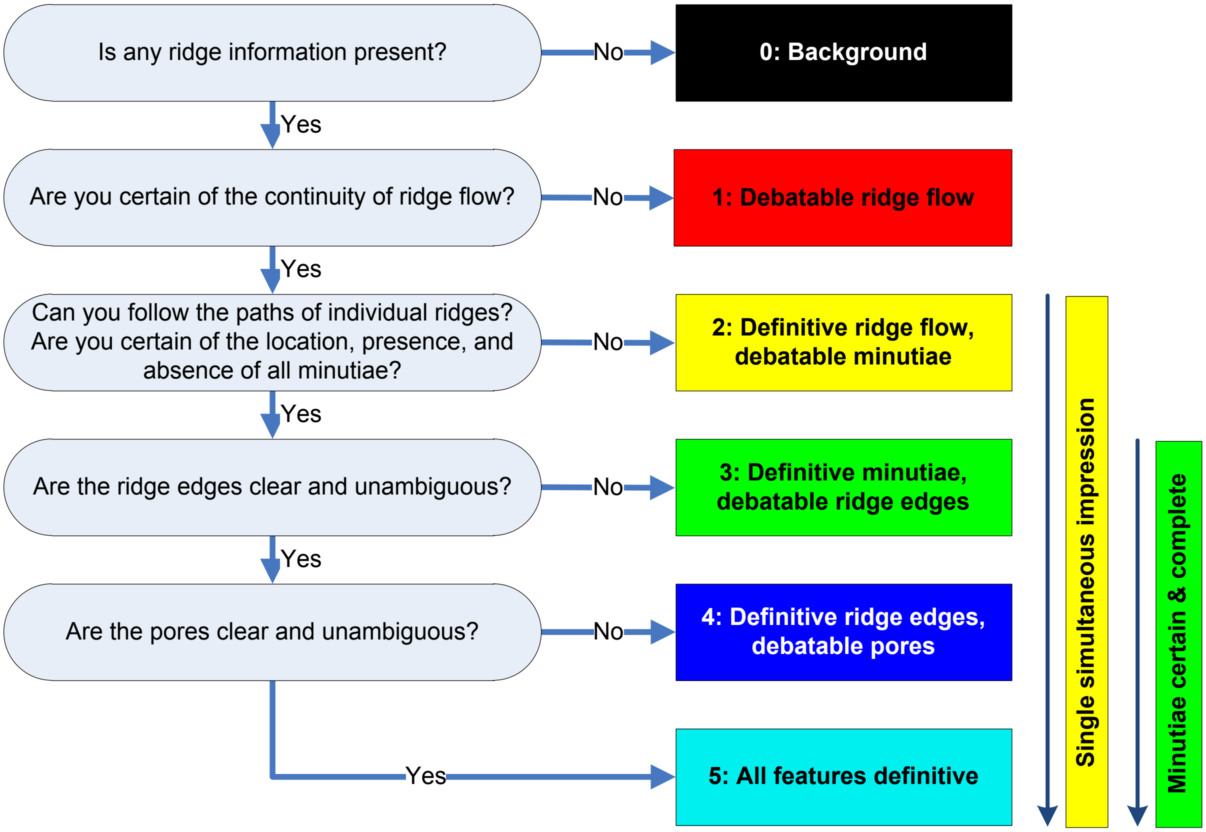

Figure 6: Definitions of ridge quality map colors.

Figure 7 through Figure 9 show examples of ridge quality markup.

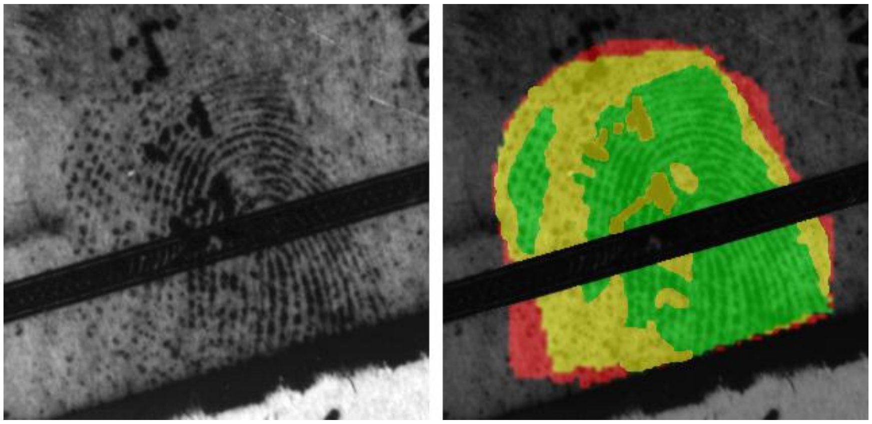

Figure 7: Ridge quality markup on a 500ppi latent.

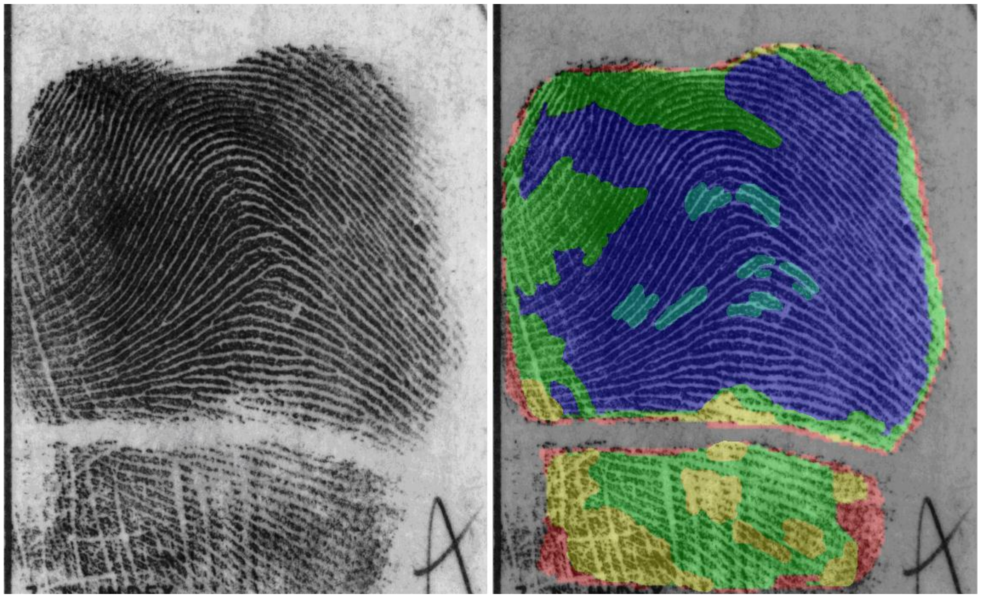

Figure 8: Ridge quality markup on an inked 500ppi exemplar.

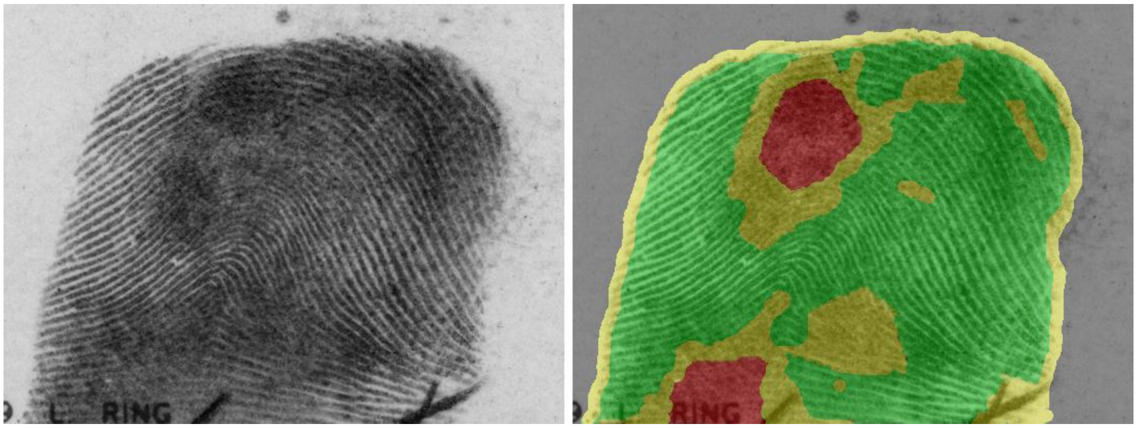

Figure 9: Ridge quality markup on an inked 500ppi exemplar.

How to paint ridge quality:

- Enable painting mode by left-clicking on the Paint Quality Map icon on the vertical toolbar.
- Change the color or size of the paint brush by right-clicking in the image while in Paint Quality Mode.
- To "erase" ridge quality, use black (background) to paint the area.

2.2 Features

When in Mark Points mode, click in the image to mark a feature. By default, the type will be minutia. To indicate a core, delta, or "other" type (such as dots or incipients), you must next right-click the point and make a selection. Cores and deltas should only be marked when they can be accurately located to within approximately three ridge intervals. Marking of "other" features is optional during latent analysis; it is only necessary for features you use as the basis for comparison determinations.

Minutiae

- The location for a **bifurcation** shall be at the "Y" of the ridge.

- The location for a **ridge ending** or **unknown type** shall be at the "Y" of the valley. Note that this definition of ridge ending location corresponds to markup for IAFIS (and NGI), but differs from some vendor-specific approaches, which may mark ridge endings on the ridge itself.

Cores are marked at the focus of the innermost recurving ridge. Note that the core is not on the innermost recurving ridgeline itself.

Deltas are marked at the center of the triradius. Deltas are marked in fingerprints for loops, whorls, and (when present) in tented arches.

Other may be used to mark features such as scars, dots, incipient ridges, creases and linear discontinuities, ridge edge features, or pores.

Direction (theta) does not need to be indicated for minutiae, cores, or deltas.

Remember that quality painting is used to indicate confidence in the presence, absence and location of minutiae:

- Green indicates that you are certain that each and every minutia in the area is marked;
- Yellow indicates that you are not confident in the presence or location of marked minutiae and there may be minutiae in the area that you did not mark.

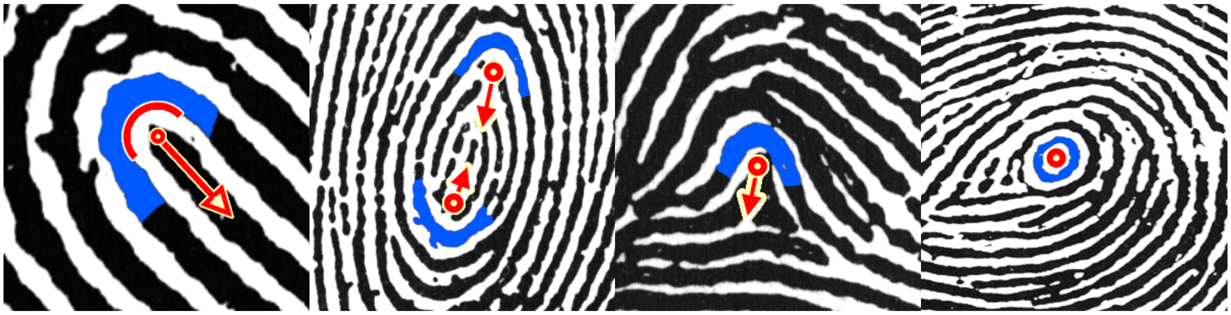

Figure 10: Cores are marked at the focus of the innermost recurving ridgeline. Examples of core locations for a right slant loop, plain whorl, tented arch, and central pocket loop whorl.

How to mark features:

- Enable feature marking by left-clicking on the Mark Points icon on the vertical toolbar.
- To mark a feature (core, delta, minutia, other) on an image, left-click at that position on the image. By default, the feature type will be minutia; to specify core, delta, or other, right click on the feature and select the appropriate type.
- To delete a marker, right-click and select "Delete Point."
- Change the color or size of markers by clicking the Display Settings button.

2.3 Corresponding features

You can indicate that two features correspond by clicking to select a feature in one image, then clicking a previously marked feature or a new point in the other image as illustrated in Figure 11. When you assess the correspondence of features during comparison, indicate the type of correspondence as summarized in Table 1. Correspondence applies to any type of feature (minutia, core, delta, other).

Type of correspondence	Description / What to mark	How to mark
Definite correspondence (both images)	The feature is definitely visible in both images.<br><br>For each feature marked in the latent, mark the corresponding feature if present in the exemplar. Mark any additional features you use in making your determination (such as incipients or level-3 detail).	Click to select a point in one image, then click a preexisting or new point in the other image.
Definite non-correspondence (single image)	A discrepancy – the feature exists in one print and is definitely not present in the other print.<br><br>Indicate points in one print that definitely do not exist in the other print as needed to support an exclusion determination	Right-click a point and select "Definite non-correspondence". Feature is displayed with "X".
Inconclusive correspondence (single image)	The feature is not visible in the other print (either obscured, or outside the corresponding area)	Mark a point in one image (and do nothing else).
Debatable correspondence (both images)	Optional: An apparent correspondence that does not rise to the threshold of definite correspondence.<br><br>Points that potentially correspond, but do not meet your threshold for supporting an ID, should be marked as debatable. This may occur, for example, when the determination is inconclusive or in low-clarity areas. In exclusion determinations, use debatable correspondence to mark reference points used to establish a discrepancy	After marking two points as corresponding, right-click either point, then select "Debatable correspondence." Feature is displayed with "?".

Table 1: Types of correspondence

- Move the cursor over a point – it will grow.
- Select the point by clicking on it – it will now stay large after you move the mouse.
- Move the cursor over the corresponding point in the other image – it will grow.
- Click the point – both points will now change colors.

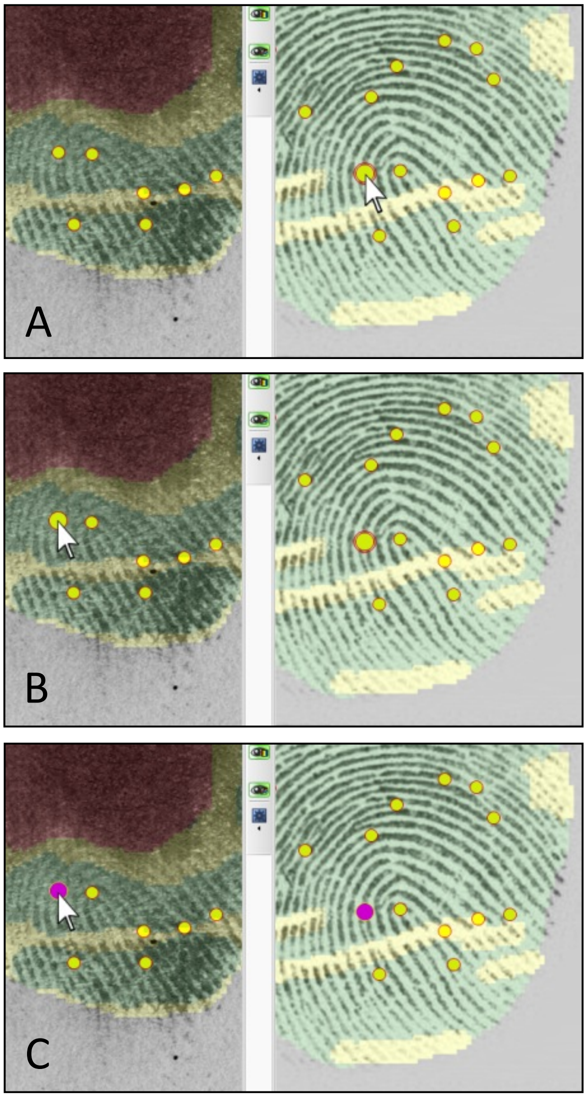

Figure 11: Marking corresponding points. Once corresponding points have been marked, moving the cursor over one point will cause it and the corresponding point to grow; this can be useful in reviewing correspondences. To break a correspondence, right-click on one point and select "unmatch point."

How to mark corresponding features:

- Left-click an existing point to select it; notice that the selected point will be larger than the others. Then click the corresponding point in the other image. The color of the two points will change to indicate that they (definitely) correspond.
- To indicate a debatable correspondence between two points, first mark the points as corresponding, then right-click either of the points and select "Debatable Correspondence" from the context menu.
- To indicate that a point definitely does not exist in the other image, first mark the point (left-click), then right-click on the marker and select "Definite Non-correspondence" from the context menu.
- Change the color or size of markers by clicking the Display Settings button.

3 ANALYSIS AND COMPARISON DETERMINATIONS

The determinations as defined here (Table 2, Table 3) may not correspond precisely to the procedures you normally follow. Please try to follow the guidance in this section as closely as possible so that responses from different people/organizations are comparable.

3.1 Value determinations

You must indicate the value of each image (latent or exemplar).

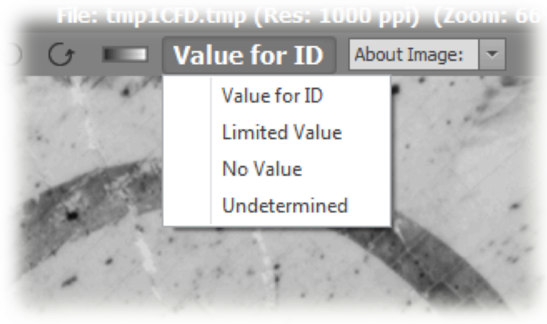

Determination	Definition
Of value for individualization *(abbreviated "Value for ID")*	The impression is of value and is appropriate for potential individualization if an appropriate exemplar is available.
Of value for exclusion only *(abbreviated "Limited value")*	The impression is NOT of value for individualization. The impression contains some friction ridge information (level 1 and/or level 2) that may be appropriate for exclusion if an appropriate exemplar is available.
No Value	The impression contains insufficient friction ridge information to reach a conclusion.

Table 2: Latent value assessment

3.2 Comparison determinations

For each comparison you must indicate a comparison determination (does not apply to latents that you determined were or no value).

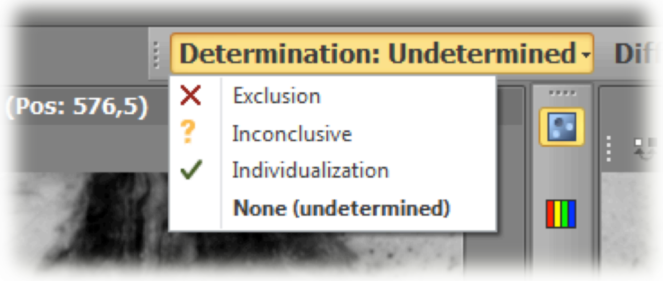

Determination	Definition
Individualization	The two fingerprints originated from the same finger.
Inconclusive	Neither individualization nor exclusion is possible.
Exclusions (of finger)	The two fingerprints did not come from the same finger.

Table 3: Comparison determination

3.3 Reason for exclusion

When the comparison determination is exclusion (of finger), you will be asked to indicate what observed differences led to that determination (Table 4). Please select the **first** option that applies.

Basis for exclusion	Definition
Pattern classes differ	The exclusion could be made based on pattern class alone.
Core or delta differences	The exclusion could be made based on one or more of the following: differing ridge flow in the cores or deltas; differing core-delta ridge counts; or differing relations among the deltas.
One or more minutiae differ	The exclusion determination could be made based on a comparison of Level-2 information.
Level 3 features differ	The exclusion determination required comparison of Level-3 information.
Other	None of the above categories satisfactorily explains the basis for the exclusion. Please briefly indicate the basis for the exclusion.

Table 4: Exclusion reason (provided only when the comparison determination is exclusion)

3.4 Difficulty

For each comparison, indicate how difficult the comparison was. Routine comparisons should be indicated as "Moderate".

Determination	Definition
Very Easy/Obvious	The comparison determination was obvious.
Easy	The comparison was easier than most latent comparisons.
Moderate	The comparison was a typical latent comparison.
Difficult	The comparison was more difficult than most latent comparisons.
Very Difficult	The comparison was unusually difficult, involving high distortion and/or other red flags.

Table 5: Comparison difficulty

3.5 Comments

For each examination, a comment box is provided to allow you to communicate concerns about the test process to the test administrators. Examples include software issues, data entry errors, particularly problematic images, any problems taking the test. The comment box is ***not*** intended to routinely capture your thought process in reaching determinations: comments should be reserved for exceptional circumstances. General comments about the White Box study will be solicited in the participant survey.

4 IMAGE PROCESSING AND MARKUP CONTROLS

The comparison tool interface is organized into several regions: latent (left side), exemplar (right side), image enhancement functions (above each image), markup functions (vertical toolbar between the two images), categorical test responses (above center), and a menu bar (at the top). A short descriptor of each button can be revealed by positioning the cursor over that button.

Figure 12 identifies software features that were not previously described.

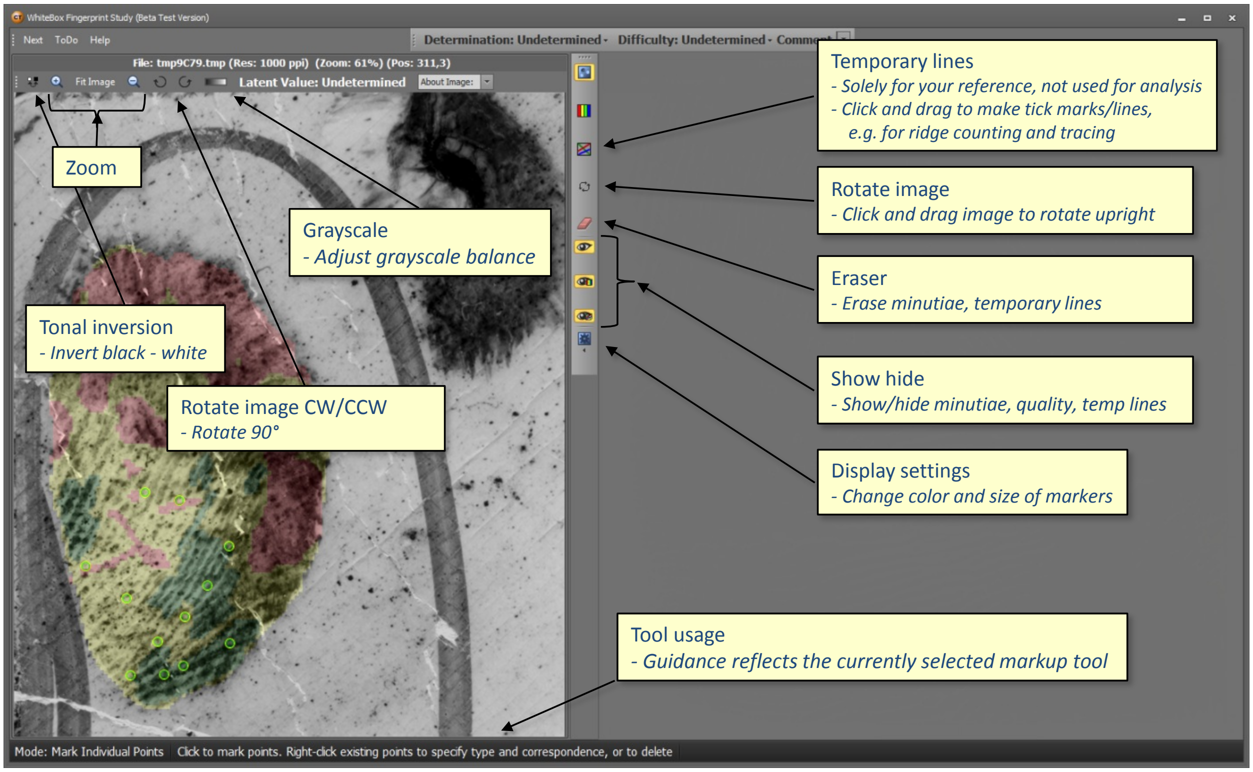

Figure 12. Image processing and markup tools. Controls specific to either the latent or exemplar are located above each image; controls on the vertical (center) toolbar apply to both images. During markup, the comparison tool will always be in one of five modes: mark points, paint quality, draw lines, dynamic rotate, or erase. Controls to select the current mode and to set display preferences are located on the vertical toolbar. Depending on the current mode and the location of the cursor, right-clicking will display a context-sensitive menu of additional options.

Painting ridge quality, marking of features, and marking of corresponding features were discussed previously; see

- Section 2.1 (Ridge quality).
- Section 2.2 (Features).
- Section 2.3 (Corresponding features).

4.1 Temporary lines

- Enable line drawing mode by left-clicking on the Draw Lines icon on the vertical toolbar.
- This function allows users to draw lines on the image, which may be helpful for ridge counting or tracing. This tool is provided as a convenience; the lines will be ignored during analysis of test results.

4.2 Erase

- Enable erasing mode by left-clicking on the Erase icon on the vertical toolbar. This tool erases marked features and temporary lines.
- With the left mouse button depressed, drag the eraser over the features or lines to be erased. Matched features will become unmatched if one of the corresponding features is erased. Note that this will not erase the corresponding feature; it will simply remove the corresponding status.

4.3 Show/Hide markup

- Three separate controls are provided to toggle whether points (marked features), quality map (painting), and temporary lines are displayed on the image(s).

- You can show/hide minutiae by hitting the spacebar (or by using the show/hide minutiae button in the middle toolbar).
- You can show/hide the quality markup by hitting ctrl-q (or by using the show/hide quality button in the middle toolbar)
- You can change the marker size, color and fill by using the Display Settings button in the middle toolbar.

4.4 Tonal reversal

- Inverts the colors in the image, so that black becomes white and white becomes black.

4.5 Zoom

The latent image and the exemplar image are linked so that when a zoom function is clicked, both images react. There are three different zoom controls:

- Zoom In or Out — Click either the "+" or the "–" buttons next to the Zoom button to zoom in or out on both images.
- Fit image — Fit the current image (latent or exemplar) to the window.

4.6 Rotation

- Fixed Rotation (above each image) – Rotate image 90 degrees
- Arbitrary Rotation (vertical toolbar) – Rotate image with hand cursor while holding down left mouse button.

4.7 Grayscale control

- Grayscale enhancement settings are reversible and not saved.
- The slider adjusts the grayscale balance of the image.
- The white and black markers define the lightest and darkest levels shown in the image.
- The gray marker defines Gamma, which defines the distribution of gray tones in the image

4.8 Display settings

Allows changing of the colors and sizes used to display features.

4.9 Shortcuts

Function	Shortcut
Zoom	Ctrl+ and Ctrl-<br>Or roll the mouse wheel
Show/hide point features	Space bar
Show/hide lines	Ctrl-L
Show/hide quality map	Ctrl-Q

In addition, the right-click menu includes a "Center Here" option as an alternative to using the scrollbars in positioning the images.
