# Supplementary material for: Measuring What Latent Fingerprint Examiners Consider Sufficient Information for Individualization Determinations
Source: PLoS One. 2014 Nov 5;9(11):e110179. doi: 10.1371/journal.pone.0110179 (PMC4221158; doi:10.1371/journal.pone.0110179)
Supplement: Information S1 — Single-file concatenation of Appendices S1 through S20. (PDF) [file pone.0110179.s021.pdf]

Measuring what latent fingerprint examiners consider sufficient information for individualization determinations

Supporting Information: Appendices

TABLE OF CONTENTS

Appendix SI-1	Glossary
Appendix SI-2	Test procedure
Appendix SI-3	Fingerprint data
Appendix SI-4	Post-processing of response data
Appendix SI-5	Summary of examiner determinations
Appendix SI-6	Timing
Appendix SI-7	Participant background survey responses
Appendix SI-8	Associations between examiners' annotations and their determinations
Appendix SI-9	Low count individualizations
Appendix SI-10	Reproducibility of individualization determinations
Appendix SI-11	Models of latent value sufficiency
Appendix SI-12	Models of sufficiency for individualization
Appendix SI-13	Repeatability of minutia counts (Analysis phase)
Appendix SI-14	Reproducibility of determinations by median corresponding minutia count
Appendix SI-15	Reproducibility of responses
Appendix SI-16	Corresponding minutia counts and determinations for nonmated image pairs
Appendix SI-17	Estimating repeatability of determinations
Appendix SI-18	Effect of point standard
Appendix SI-19	Minimum number of corresponding minutiae
Appendix SI-20	White Box Latent Examiner Study — Instructions

Appendix SI-1 Glossary

This section defines terms and acronyms as they are used in this paper.

**ACE**	The phases of ACE-V prior to verification: Analysis, Comparison, Evaluation.
**ACE-V**	The prevailing method for latent print examination: Analysis, Comparison, Evaluation, Verification.
**AFIS**	Automated Fingerprint Identification System (generic term)
**Analysis phase**	The first phase of the ACE-V method. In this test, the examiner annotated the latent and made a value determination before seeing the exemplar print. In this report, we capitalize Analysis when referring to the ACE phase.
**ANSI/NIST-ITL**	An electronic file and interchange format that is the basis for biometric and forensic standards used around the world, including the FBI's EBTS and Interpol's INT-I, among others. As of 2011, this incorporates the Extended Feature Set (EFS) definition of friction ridge features used in this study [1].
**Clarity**	The clarity of a friction ridge impression refers to the fidelity with which anatomical details are represented in a 2D impression, and directly corresponds to an examiner's confidence that the presence, absence, and details of the anatomical friction ridge features in that area can be correctly discerned in that impression. (Note: The term "clarity" is used here instead of "quality" to avoid ambiguity, since the latter term as used in biometrics and forensic science is often used to include not only clarity but also the quantity or distinctiveness of features.)
**Comparison/Evaluation phase**	The second and third phases of the ACE-V method. In this test, there was no procedural demarcation between the Comparison and Evaluation phases of the ACE-V method; hence, this refers to the single combined phase during which both images were presented side-by-side. For brevity, in this report we use "Comparison" to refer to the Comparison/Evaluation phase.
**Comparison determination**	The determination of individualization, exclusion, or inconclusive reached in the Comparison/Evaluation phase of the test. SWGFAST [2] refers to this determination as the Evaluation Conclusion.
**Corresponding clarity map**	The corresponding clarity map represents the minimum clarity at each location in the aligned latent and exemplar clarity maps, as described in [3]. These maps were constructed from the examiners' annotations by post-processing software whenever at least three corresponding features were marked by the examiner. A thin-plate spline algorithm was used to align the latent and exemplar prints. (See local clarity map)
**Corresponding features**	A 1:1 relationship between a feature in a latent and a feature in the exemplar in which the feature is present in both images.
**Debatable correspondence**	A relationship between a feature in a latent and a feature in the exemplar in which there is an apparent correspondence between a feature in the latent and a feature in the exemplar that does not rise to the threshold of definite correspondence. (Not to be confused with debatable ridge flow or debatable features, which were indicated by painting the image clarity.)
**Determination**	An examiner's decision: the Analysis phase results in a Value determination, and the Comparison/Evaluation phase results in a Comparison determination.
**Exclusion**	The comparison determination that the latent and exemplar fingerprints did not come from the same finger. For our purposes, this is *exclusion of source*, which means the two impressions originated from different sources of friction ridge skin, but the subject cannot be excluded, whereas *exclusion of subject* means the two impressions originated from different subjects.
**Exemplar**	A fingerprint from a known source, intentionally recorded.
**Extended Feature Set**	The definition of friction ridge features incorporated into the ANSI/NIST-ITL standard.
**False negative**	An erroneous exclusion of a mated image pair by an examiner.
**False positive**	An erroneous individualization of a nonmated image pair by an examiner.
**Feature**	Minutia, core, delta, or "other" point marked by examiners. In this study, a feature has a location (x,y coordinate) but no direction.
**IAFIS**	The FBI's Integrated Automated Fingerprint Identification System (as of 2013, IAFIS latent print services have been replaced by the FBI's Next Generation Identification (NGI) system).
**IAI**	International Association for Identification
**Image**	A fingerprint as presented on the computer screen to test participants. The test software permitted rotating, panning, zooming, tonal inversion, and grayscale adjustment of the image.
**Incipient ridge**	A friction ridge not fully formed that may appear shorter and thinner in appearance than fully developed friction ridges.
**Inconclusive**	The comparison determination that neither individualization nor exclusion is possible.
**Individualization**	The comparison determination that the latent and exemplar fingerprints originated from the same source. Individualization is synonymous with identification for latent print determinations in the U.S. Both are defined as: "the decision by an examiner that there are sufficient discrimination friction ridge features in agreement to conclude that two areas of friction ridge impressions originated from the same source. Individualization of an impression to one source is the decision that the likelihood the impression was made by another (different) source is so remote that it is considered as a practical impossibility." [2,4]
**Insufficient**	When referring to examiner determinations (response data), "Insufficient" responses include both no value and inconclusive determinations.
**Latent (or latent print)**	A friction ridge impression from an unknown source. In North America, "print" is used to refer generically to known or unknown impressions [5]. Outside of North America, an impression from an unknown source (latent) is

	often described as a "mark" or "trace," and "print" is used to refer only to known impressions (exemplars).
**Level-3 detail**	Friction ridge dimensional attributes such as width, edge shapes, and pores.
**Local clarity map**	A color-coded annotation of a friction ridge image indicating the clarity for every location in the print, as described in [3] and defined in the ANSI/NIST-ITL standard [1].
**Mated**	A pair of images (latent and exemplar) known *a priori* to derive from impressions of the same source (finger). Compare with "individualization," which is an examiner's ***determination*** that the prints are from the same source.
**Median clarity map**	A local clarity map combining the annotations from multiple examiners, based on the median clarity at each location across the clarity maps from all examiners who annotated the clarity of an image (or image pair, for median corresponding clarity maps).
**Minutiae**	Events along the path of a single path, including bifurcations and ending ridges. In this study, examiners did not differentiate between bifurcations and ending ridges. Dots are considered minutiae in some uses, but not for AFIS usage; in this study, examiners were instructed to mark dots as "other" features.
**Misclassification rate**	The proportion of responses that would be incorrectly classified as individualization or not individualization for a given model.
**Missed ID**	Failure by an examiner to individualize a mated pair that was individualized by any other examiners (also known as a "missed individualization" or "missed identification").
**Noncorresponding feature**	A discrepancy – a feature that exists in one print and is definitely not present in the other print. Participants were instructed to indicate points in one print that definitely do not exist in the other print as needed to support an exclusion determination.
**Nonmated**	A pair of images (latent and exemplar) known *a priori* to derive from impressions of different sources (different fingers and/or different subjects).
**NV (No value)**	The impression is not of value for individualization and contains no usable friction ridge information. See also VEO and VID.
**Other point**	In this study, features such as scars, dots, incipient ridges, creases and linear discontinuities, ridge edge features, or pores (i.e., features other than minutiae, cores, and deltas).
**Overall Clarity**	A metric based on the size and consistency of the areas of the various levels of clarity in a local clarity map, described in [3]. Overall Clarity ranges from 0-100 and was developed to correspond to human examiner assessments of the value and difficulty of an image.
**Qualified examiner**	Determined by an agency to be appropriately qualified as a latent print examiner. Used instead of "certified" in some organizations to differentiate from the IAI certification, "Certified Latent Print Examiner."
**Repeatability**	Intraexaminer agreement: when one examiner provides the same response (annotation or determination) to a stimulus (image or image pair) on multiple occasions.
**Reproducibility**	Interexaminer agreement: when multiple examiners provide the same response (annotation or determination) to a stimulus (image or image pair).
**Source**	An area of friction ridge skin from which an impression is left. Two impressions are said to be from the "same source" when they have in common a region of overlapping friction ridge skin.
**Sufficient**	An examiner's assessment that the quality and quantity of information in a print (or image pair) justifies a specific determination (especially used with respect to individualization).
**SWGFAST**	Scientific Working Group on Friction Ridge Analysis, Study and Technology
**ULW**	The FBI's Universal Latent Workstation software [6].
**Unassociated feature**	A feature marked in one print for which the examiner did not indicate any level of correspondence or non-correspondence with respect to the other print (often either obscured or outside the corresponding area).
**Value determination**	An examiner's determination of the suitability of an impression for comparison: value for individualization (VID), value for exclusion only (VEO), or no value (NV). A latent value determination is made during the Analysis phase. Agency policy often reduces the three value categories into two, either by combining VID and VEO into a value for comparison (VCMP) category or by combining VEO with NV into a "not of value for individualization" (Not VID) category [survey in 7].
**VCMP**	Value determination based on the analysis of a latent that the impression is of value for comparison (either VEO or VID).
**VEO**	Value determination based on the analysis of a latent that the impression is of value for exclusion only and contains some friction ridge information that may be appropriate for exclusion if an appropriate exemplar is available. See also NV and VID.
**Verification**	The final phase of ACE-V: the independent application of the ACE process by a subsequent examiner to either support or refute the conclusions of the original examiner. Not addressed in this study.
**VID**	Determination based on the analysis of a latent that the impression is of value and is appropriate for potential individualization if an appropriate exemplar is available. See also VEO and NV.

1 National Institute of Standards (2011) American National Standard for Information Systems: Data format for the interchange of fingerprint, facial & other biometric information. ANSI/NIST-ITL 1-2011. (http://fingerprint.nist.gov/standard)

2 SWGFAST (2013) Standards for Examining Friction Ridge Impressions and Resulting Conclusions, Version 2.0. (http://www.swgfast.org/documents/examinations-conclusions/130427_Examinations-Conclusions_2.0.pdf)

3 Hicklin RA, Buscaglia J, Roberts MA (2013) Assessing the clarity of friction ridge impressions. *Forensic Sci Int* **226(1)**:106-117.

4 SWGFAST (2012) Individualization / Identification Position Statement, Version 1.0. (http://swgfast.org/Comments-Positions/120306_Individualization-Identification.pdf)

5 SWGFAST (2011) Standard terminology of friction ridge examination, Version 3.0. (http://swgfast.org/documents/terminology/110323_Standard-Terminology_3.0.pdf)

6 Federal Bureau of Investigation; Universal Latent Workstation (ULW) Software. (https://www.fbibiospecs.org/Latent/LatentPrintServices.aspx)

7 Ulery BT, Hicklin RA, Buscaglia J, Roberts MA (2011) Accuracy and reliability of forensic latent fingerprint decisions. Proc Natl Acad Sci USA **108(19)**: 7733-7738. (http://www.pnas.org/content/108/19/7733.full.pdf)

Appendix SI-2 Test procedure

The tests were administered using a customized version of the FBI's Universal Latent Workstation (ULW) Comparison Tool, distributed on DVDs [1]. Participants were required to sign and return an informed consent form approved by the FBI's Institutional Review Board before receiving the disks. The fingerprint files were encrypted to limit reuse; all participants agreed to return or destroy the DVDs, and uninstall the data and software from all computers. Each participant was assigned a unique identifier, which was associated with a randomized assignment of image comparisons and was used to anonymize results. Test instructions were provided in paper and digital formats (Appendix SI-20) and as an introductory video (Supporting Information S21). Examiners outside the U.S. were advised that a moderate level of English fluency was necessary to understand the test instructions. A set of practice data was included so that participants could become familiar with the software and procedures before starting the test. After completing all examinations, the examiners were required to complete a survey (Appendix SI-7) before submitting test responses. When each participant completed the test, the test software packaged the response data as encrypted files with the fingerprint images removed to limit file size for transmission.

The differences between the White Box software and the ULW Comparison Tool were generally to enable test administration (e.g., handling encrypted data files, presenting specific image pairs to each participant, and submission of results); the White Box software did not include ULW's "ghost cursor" tool, which assists examiners in locating correspondences.

The White Box software included tools for annotation of features, correspondence of features, and ridge clarity. One tool permitted drawing of temporary lines to assist with ridge counting and following ridges; these were not used in analysis. A minimal set of tools was provided for viewing the images (zoom, rotation, scrollbars, tonal reversal, and grayscale controls); more sophisticated tools were not included to limit the effect of differences among examiners regarding expertise with software tools. Additional controls were provided to show or hide the annotation.

Participants were instructed that the computer screen must accurately display the images presented by the software, and the software included a check for that purpose. They were instructed that it was imperative that they conduct the examinations in this study with the same diligence that they would use in performing casework. The test environment was not controlled. Participants were given several weeks to complete the test, but there were no specific time requirements. They were not told what proportion of the image pairs were mated, nor was any feedback on the correctness of responses provided during the test. Participants were able to review and revise their responses before proceeding to the next comparison, revisit previous comparisons, and skip analyses and comparisons and return to them later. Once an examiner indicated that the Analysis phase was complete and proceeded to comparison, the Analysis phase markup and determination were recorded; any subsequent revisions were recorded as part of the Comparison/Evaluation phase. Examiners were able to leave comments associated with specific test responses. Based on these comments, results of one examiner were excluded from analysis (see Appendix SI-5); no other irregularities were mentioned that required excluding data or special handling.

Alpha and beta test releases were used to solidify the software, instructions, and process. The test was distributed to participants in tiered releases to minimize project risks.

Results are anonymous. A coding system was used to ensure anonymity so that even the analysis team could not associate test responses to individual participants. Because of this, there is no way to cross-reference any participants who may have taken both the Black Box and White Box studies.

The white box software enforced the following errors and warnings, which were shown as dialog boxes by the software when the examiner attempted to complete Analysis or Comparison. Errors prevented the examiner from concluding Analysis or Comparison; for warnings the examiner had to click "OK but could be ignored.

Errors and warnings in Latent Analysis:

- Error: Latent Value determination has not been selected
- Error: Latent Quality is not painted
- Warning: Latent is Of Value for ID but fewer than 6 points are marked

Errors and warnings in Comparison:

- Error: Exemplar Value determination has not been selected
- Error: Exclusion determination and latent is of no value
- *[If latent value is set to No Value during Comparison]* Error: Latent value determination has not been selected
- Error: A comparison determination has not been selected
- Error: Individualization determination and latent is of limited value (value for exclusion only)
- Error: A comparison determination has not been selected
- Error: Exclusion determination and exemplar is of no value
- Error: Individualization determination and exemplar is of no value
- Error: A comparison determination has not been selected
- Error: Individualization determination and exemplar is of limited value (value for exclusion only)
- Error: A comparison determination has not been selected
- Error: Exemplar Quality is not painted
- Error: Comparison difficulty has not been selected
- Warning: Individualization determination and fewer than 6 corresponding features marked
- Warning: Individualization determination and no corresponding features marked
- Warning: Individualization determination, but non-corresponding points were also marked

1 Federal Bureau of Investigation; Universal Latent Workstation (ULW) Software.
(https://www.fbibiospecs.org/Latent/LatentPrintServices.aspx)

Appendix SI-3 Fingerprint data

The fingerprints for the study were collected at the FBI Laboratory and at Noblis under controlled conditions, and from operational casework datasets collected by the FBI. The collection of fingerprint data from human subjects was approved by the FBI Institutional Review Board and the Noblis Institutional Review Board.

The latents used in the study were selected from a broader set of several thousand latent fingerprints. They were processed using black powder, ninhydrin, physical developer, cyanoacrylate, RUVIS (Reflective Ultra Violet Imaging Systems), RAM Cyanoacrylate fluorescent dye (Rhodamine 6G; Ardrox; 7-p-methoxybenzylamino-4-nitrobenz-2-oxa-1, 3-diazole), or DFO (1,8-diazafluoren-9-one). The processed latents were captured electronically at 8-bit grayscale, uncompressed, at a resolution of 1000 pixels per inch: lift cards and porous materials (processed using ninhydrin or DFO) were scanned using flatbed scanners; latents on nonporous materials (processed using black powder, or cyanoacrylate and light gray powder) were captured using digital cameras.

The exemplars included rolled and plain impressions and were captured as inked prints on paper cards or using FBI-certified livescan devices; exemplars were 8-bit grayscale, 1000 or 500 pixels per inch and were either uncompressed or compressed using Wavelet Scalar Quantization [1]. Nonmated pairs were selected to result in challenging comparisons. They were prepared by down-selecting among exemplar prints returned by searches of the FBI's Integrated AFIS (IAFIS) and among neighboring fingers from the same subject.

The test was initially released to a small subset of the final participants to minimize project risks. Some of these early participants noted a few of the latents were scanned from images that included physical marks indicating minutiae. In response, we reviewed our data and identified seven such images. Each of the seven image pairs was replaced by another pair of the same mating and same preliminary bin classification. The ten responses to the removed image pairs were excluded from all analyses.

The final test design included 320 image pairs (231 mated and 89 nonmated), for an average of five sample image pairs in each of the 64 bins; the actual number of image pairs per bin varied from three to six, as samples of some combinations of fingerprint attributes were relatively difficult to obtain. Some of the latents were used in both mated and nonmated pairs: the 320 image pairs were constructed from 301 latents (from 247 distinct fingers) and 319 exemplars (from 276 distinct fingers). Of the 301 latents, 184 were collected under controlled conditions and 117 were from operational casework. All prints were impressions of distal segments of fingers, including some sides and tips.

The assignments of fingerprint images to examiners were randomized based on an Incomplete Block Design (with examiners as blocks, image pairs as factor levels), balanced to the extent possible using the criterion of D-Optimality, yielding an the experimental design with nearly the same statistical properties as a fully balanced design. Separate designs were constructed for mated and for nonmated pairs. Note that image pairs were used as the factor levels rather than the bins themselves. This decision was based in part on the fact that the same source fingers often contributed to multiple bins and we did not want to assign any one participant two prints from the same finger. Given that (1) there was no "ground truth" for the preliminary bin assignments and those assignments were expected to be imprecise and (2) each examiner was assigned only 22 image pairs from a design space of 64 bins, this seemed a safe approach. After creating the design, we confirmed the validity of this approach: the D-Efficiency, a measure of balance, was calculated for the design with respect to models involving 1-, 2-, 3-, and 4-way interactions of the 4 factors defining the bins. The metric was reasonably close to 100% for models up to 2-way interactions, though somewhat less for models with higher-order interactions.

Early in design we collected fingerprints and assigned those prints to bins before we knew how many participants to expect. Trainees at the FBI were given sets of approximately 25 mated image pairs, informed that they were in fact mated pairs, and asked to quickly characterize each pair per the questions shown in Table S1; one of the authors performed a similar procedure for nonmates. This rapid screening was used for data selection and assignment to bins, not for analysis. Additional prints were collected as needed until we had at least three sample pairs per bin. Late in the design process, after many volunteers had formally expressed interest in the test, we finalized the incomplete block design sized to 210 participants. The number of people who responded to the call for participation, and actually returned results, remained in flux during the testing period.

Can a corresponding area be determined? (if not, there is no overlap, no basis for comparison)

- Yes - there is a potentially corresponding area
- No - there is no overlap, no basis for comparison

Estimated number of minutiae in correspondence

- No minutiae
- 1-4 minutiae
- 5-8 minutiae
- 9-12 minutiae
- 13-20 minutiae
- 21-30 minutiae
- 31+ minutiae

Number of cores present in both prints

- 0
- 1
- 2

Number of deltas present in both prints

- 0
- 1
- 2

Overall clarity of latent

- Low: at least 3/4 of the usable area is limited to ridge flow information; minutiae in this area are “debatable.”
- Medium: neither Low nor High (mixed clarity)
- High: the presence or absence of minutiae is certain throughout least 3/4 of the usable area

Is the exemplar quality unusually poor?

- Poor quality exemplar
- Normal exemplar

Is there substantial distortion in the latent? (e.g. twisting, slippage, compressed/stretched ridges)

- High: Distortion is a major factor complicating the comparison
- Medium: Distortion is moderate, but not a major factor complicating the comparison
- Low: Distortion does not complicate the comparison

Does the processing method make interpretation difficult?

- Yes - the processing method makes interpretation particularly difficult
- No

Do the two images have unusually distinctive features in common (e.g., scar, unusual pattern)

- Yes - distinctive features in common
- No

Do the two images have substantial level 3 data in common?

- Yes - substantial level 3 in common
- No

Are there superimpositions, discontinuous impressions or other red flags?

- Yes - there are superimpositions, discontinuous impressions or other red flags
- No

Is either image flipped left-right?

- Yes (flipped)
- No

These two images are supposed to have come from the same finger. If you think this is not correct, please indicate:

- Obvious exclusion (not the same finger)
- Probable exclusion (I am reasonably sure these are not from the same finger)
- Fine as is (no reason to believe these are not the same finger)

Are there any other data problems such as blank or invalid images, multiple impressions where it's not obvious which impression to use, or anything else that we should review for this pair of images?

- Yes (data problem)
- No

Table S1: Questions asked of latent print examiner trainees to screen mated pairs for possible inclusion in the study. Responses to these questions were used to select image pairs for the test and to assign selected image pairs to preliminary bins.

1. Criminal Justice Information Services (1997) Wavelet Scalar Quantization (WSQ) Gray-Scale Fingerprint Image Compression Specification, Version 3.1. (https://www.fbibiospecs.org/docs/WSQ_Gray-scale_Specification_Version_3_1_Final.pdf)

Appendix SI-4 Post-processing of response data

For each image pair assigned to an examiner, two data files were produced: one saved upon completion of the Analysis phase (before the exemplar print was presented) and a second after completion of Comparison/Evaluation phase. The files complied with the ANSI/NIST-ITL [1] standard, using the COMP transaction described in the Latent Interoperability Transmission Specification [2]. All collected data was included in standard fields, with the exception of the comparison difficulty, the exclusion reason (collected in temporary fields), and the temporary lines (not used for evaluation; field expected to be added to a 2013 revision of the standard).

Upon receipt, the files were decrypted, images were reinserted, and the contents were validated. Validation included some of the processing steps below to perform a cursory check that the data was intact and appeared to contain complete annotation and determinations.

Post-processing software derived tabular data from the ANSI/NIST COMP files. Initially, summary data was produced for each fingerprint (latent and exemplar separately) and separately for each feature annotated. The summary data for a fingerprint includes counts of features with some breakdown by feature type and the local clarity for the features, various measures pertaining to the area annotated at each clarity level, and the categorical responses (determinations and difficulty). The summary data for each feature includes its type, clarity, and location; data for features annotated as corresponding between the latent and exemplar were linked. For a full description of the available annotation data (feature types, correspondence types, clarity levels, etc.), see the test Instructions (Appendix SI-20).

The summary data describing clarity annotation includes the area at each clarity level, measures of largest contiguous area at each clarity level, and related measures [3]. For each type of annotation (latent Analysis, latent and exemplar Comparison, and derived corresponding clarity) median clarity maps were produced. Each median clarity map represents the median clarity at each (x, y) location on an image as calculated from a set of clarity maps produced by individual examiners for the same image. The set of clarity maps used to produce the median maps excludes responses from four examiners whose clarity annotation did not comply with the test instructions. The median clarity maps conformed well to our expectations of proper and careful characterizations of latent clarity, by reducing the impact of outlier opinions and imprecision.

The tabular summary response data was joined with data from the experimental design, including in particular whether or not the image pairs were mated, and also meta-information about the fingerprint images and the screening bins used to balance the assignments. These tables, together with the actual images and visualization of the annotation, were the primary basis for analyses. Additional tools and information were used to identify and analyze outliers.

Numerous anomalies were observed in the examiner response files. Most of these pertained to specific aspects of the annotation and often there was no objective criterion for differentiating extreme, but legitimate responses from outright errors and failures to comply with specific instructions. Most of these anomalous annotations were perfectly usable in most regards. Some common examples include annotating nonminutia features as minutiae (the default feature type), failing to expressly correspond pairs of features that were annotated in both the latent and exemplar, and using clarity colors improperly (e.g., annotating debatable ridge flow as background). We chose to keep records of anomalies we discovered and be cognizant of such outliers when interpreting the results rather than exclude them from analysis. Anomalous responses were sometimes excluded from voted metrics. For example, many examiners never annotated cores or deltas, so these examiners were excluded from the construction of metrics describing the proportion of examiners who marked cores or deltas. As a rule, the percentage of responses containing problematic measurements was sufficiently low as to have limited deleterious effects on the models; also, robust statistics, such as median, were often selected to limit these effects and help interpret the data.

Statistical analyses and modeling were conducted with SAS JMP® version 10 [4].

1 National Institute of Standards (2011) American National Standard for Information Systems: Data format for the interchange of fingerprint, facial & other biometric information. ANSI/NIST-ITL 1-2011. (http://fingerprint.nist.gov/standard)

2 Chapman W, et al (2013) Latent Interoperability Transmission Specification. NIST Special Publication 1152. US Department of Commerce, National Institute of Standards and Technology, Washington, DC. (http://nvlpubs.nist.gov/nistpubs/SpecialPublications/NIST.SP.1152.pdf)

3 Hicklin RA, Buscaglia J, Roberts MA (2013) Assessing the clarity of friction ridge impressions. Forensic Sci Int 226(1):106-117.

4 JMP® statistical software, SAS Institute Inc., Cary, NC, USA, Copyright 2012 (www.jmp.com)

Appendix SI-5 Summary of examiner determinations

We received data from 172 examiners: one examiner did not take the test seriously and commented that the results should be disregarded; another examiner returned responses to practice problems only. This resulted in a total of 3740 responses received and fully processed (170 examiners x 22 assignments) for each of Analysis and Comparison. As discussed in Appendix SI-3, responses on seven image pairs were excluded, yielding a total of 3730 valid responses from the Analysis phase (mean 12.4 examiners per latent).

For our analyses of mated pairs, we required valid annotations and determinations from both phases. Among the 2882 responses to mated pairs (see Table S2), we additionally excluded one file that was missing a Comparison determination and all responses from five examiners who routinely did not annotate correspondences. This resulted in 2796 valid responses (mean 12.1 responses per mated pair). Some analyses omit the 125 erroneous exclusions, resulting in 2671 mated image pairs. We did not omit any additional responses that we considered to be improperly annotated.

Comparison Determination	Latent Value	Total	Mates (Invalid)	Mates (valid)	Nonmates (all)
	NV (in Analysis)	713	21	441	251
	NV (in Comparison)	43	1	28	14
Exclusion	VEO	131	2	27	102
Exclusion	VID	430	4	98	328
Inconclusive	VEO	359	5	268	86
Inconclusive	VID	346	6	275	65
Individualization	VID	1700	46	1653	1
(Exemplar NV)	VEO	3	0	3	0
(Exemplar NV)	VID	3	0	3	0
(invalid determination)	VEO	1	0	0	1
(invalid determination)	VID	1	1	0	0
Totals		3730	86	2796	848
Total comparisons	VCMP (either VEO or VID)	2974	64	2327	583
Total comparisons	VID	2480	57	2029	394

Table S2: Final determinations by 170 examiners on 320 image pairs. The latent value column reports the final value determination (after any Comparison phase changes in value assessments).

Fig. S1 shows that the sample fingerprints selected for the test were well balanced with respect to our objective of better understanding what constitutes sufficiency for individualization. The majority of latents were of marginal value for individualization but included a small proportion in which examiners were unanimous in their determinations. Likewise, the mated image pairs were predominantly of marginal sufficiency for individualization and there were samples at either extreme. This distribution ensured an efficient design for answering our research questions. One effect of this design (focusing on marginal cases), is that the rates of interexaminer reproducibility of determinations were reduced relative to our previous Black Box study [1].

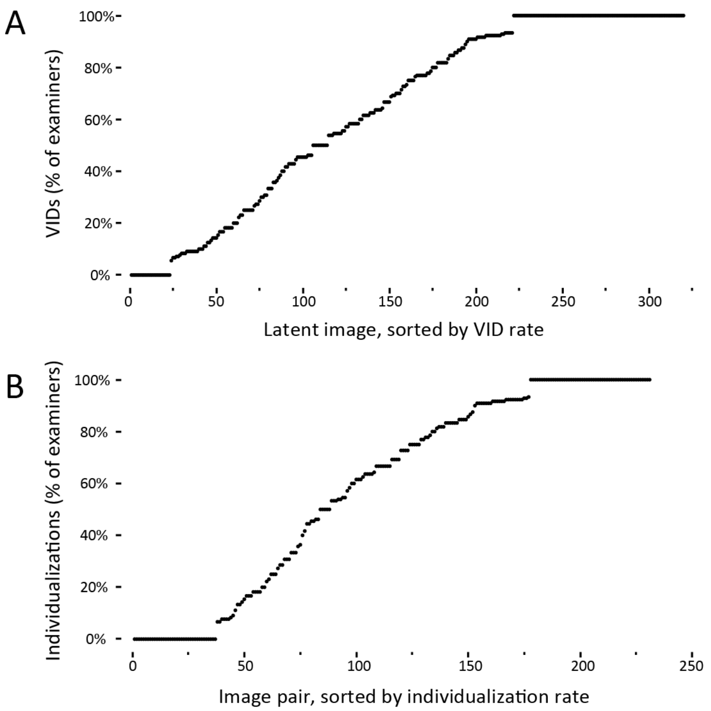

Fig. S1: Reproducibility of latent value and individualization determinations. Percentage of examiners (y-axis) who made (A) VID determinations for each latent (x-axis) and (B) Individualization determinations for each mated pair (x-axis). Includes 3730 latent value determinations from 170 examiners on 301 latents, and 2796 responses by 165 examiners on 231 mated pairs.

This data sampling strategy is neither designed, nor well-suited, for estimating operational error rates; however, we note that the error rates on this test were similar to those we measured in our Black Box study. The single false positive error (among 583 nonmated comparisons) was consistent with the rate of 6/4083 observed in the Black Box study [2]. The false negative error rate was lower on this test (5.5% of mated comparisons vs. 7.5% in the Black Box study). In addition to differences in data sampling, other factors might have contributed to this lower false negative error rate: the Black Box study alerted the fingerprint community to a high false negative rate, so the examiners may have modified their behavior; the process of providing detailed markup may have influenced some of the determinations; there were some differences in participants between the two studies, notably a greater number of non-U.S. participants in this study.

1 Ulery BT, Hicklin RA, Buscaglia J, Roberts MA (2012), Repeatability and Reproducibility of Decisions by Latent Fingerprint Examiners. PLoS ONE 7:3. (http://www.plosone.org/article/info:doi/10.1371/journal.pone.0032800)

2 Ulery BT, Hicklin RA, Buscaglia J, Roberts MA (2011) Accuracy and reliability of forensic latent fingerprint decisions. Proc Natl Acad Sci USA 108(19): 7733-7738. (http://www.pnas.org/content/108/19/7733.full.pdf)

Appendix SI-6 Timing

Fig. S2 summarizes the time taken by examiners for Analysis and Comparison: 80% of examiners took 3 to 26 minutes per image for latent analysis; 80% of examiners took 5 to 46 minutes per image pair for comparison. Some examiners reported spending up to about 20 hours to complete the study. Times are based on the examiner's opening and closing of images for Analysis or image pairs for Comparison. Examiners were allowed to revisit work; hence, elapsed times were constructed as totals across multiple sessions. Interruptions, such as phone calls or lunch breaks may have substantially affected many of these measurements, so they should be taken as upper bounds on the time actually taken for examination itself. Examiners were not informed that timing measurements were being recorded.

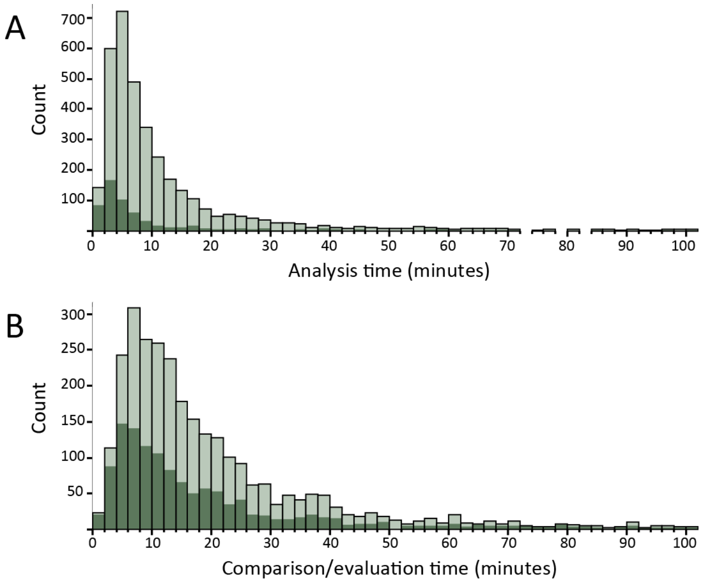

Fig. S2: Elapsed times in minutes for (A) Analysis phase, NV determinations highlighted; and (B) Comparison/Evaluation phase, non-individualization determinations highlighted.

Appendix SI-7 Participant background survey responses

The following table summarizes responses to the background survey, which participants completed after submitting their test data. One participant did not complete the survey, so responses total 169.

	Count	*%*
**1. Sex**		
• Female	98	58.0%
• Male	71	42.0%
**2. Age**		
• 18-29	28	16.6%
• 30-39	71	42.0%
• 40-49	29	17.2%
• 50-59	30	17.8%
• 60-69	11	6.5%
**3. Highest level of education achieved**		
• High School Diploma/GED	13	7.7%
• Associate Degree / some college	20	11.8%
• Bachelor's Degree	76	45.0%
• Graduate Degree / Professional Degree	60	35.5%
**4. Current Employment**		
• U.S. Federal government	71	42.0%
• U.S. State government	24	14.2%
• U.S. City/County government	36	21.3%
• U.S. Private sector (non-government)	7	4.1%
• Non-U.S. National/Federal government	24	14.2%
• Non-U.S. State/Provincial government	5	3.0%
• Non-U.S. Local/ City/County government	1	0.6%
• Non-U.S. Private sector (non-government)	1	0.6%
**5. Has your agency received accreditation in latent prints?**		
• Yes - for example, by American Society of Crime Laboratory Directors/Laboratory Accreditation Board (ASCLD/LAB), Forensic Quality Services (FQS), or International Organization for Standardization (ISO/IEC 17025)	113	66.9%
• No	50	29.6%
• Don't know	6	3.6%
**6. Total number of years employed as a latent examiner**		
• Less than 1	0	0.0%
• 1-4	40	23.7%
• 5-9	59	34.9%
• 10-19	37	21.9%
• 20-29	20	11.8%
• 30-39	11	6.5%
• 40 or more	2	1.2%
**7. Type of latent training received**		
• Formal program of instruction for 1 year or more	106	62.7%
• Formal program of instruction for 6 months to 1 year	35	20.7%
• Limited formal training (courses, workshops) for less than 6 months	19	11.2%
• Other	9	5.3%

	Count	%
**8. Are you certified as a latent print examiner? (Check all that apply - may add up to more than 100%)**		
• International Association for Identification (IAI) Certified Latent Print Examiner (CLPE)	56	33.1%
• Certified or qualified as a latent print examiner by a current or previous employer	97	57.4%
• National certification (non-US only)	19	11.2%
• Other certification	5	3.0%
• No certification	19	11.2%
**9. Are you currently conducting latent examinations on a regular basis (at least weekly over an extended period?)**		
• No	2	1.2%
• No, but I have previously conducted latent examinations on a regular basis	16	9.5%
• Yes	151	89.4%
**10. What percentage of time have you spent over the last year doing latent comparisons?**		
• None: I am not performing comparisons	0	0.0%
• Less than 10%	12	7.1%
• 10-25%	21	12.4%
• 25-50%	23	13.6%
• 50-75%	46	27.2%
• 75-100%	67	39.6%
**11. Of the latent-to-exemplar comparisons you have performed over the last year, what proportion do you perform on computer screens, as opposed to looking at physical evidence/paper cards?**		
• 0% computer	12	7.1%
• 1-30% computer	68	40.2%
• 30-60% computer	35	20.7%
• 60-99% computer	42	24.9%
• 100% computer	12	7.1%
**12. Of the latent-to-exemplar comparisons you have performed over the last year, what proportion of the conclusions were based on a single exemplar print (e.g. based on the rolled exemplar without reference to the plain exemplar or additional sets of exemplar fingerprints)?**		
• None	13	7.7%
• Less than 10%	48	28.4%
• 10-25%	16	9.5%
• 25-50%	23	13.6%
• 50-75%	40	23.7%
• 75-100%	29	17.2%
**13. Does your organization permit an official conclusion of less than individualization, more than inconclusive, such as "limited match" or "qualified identification"? (Given the standard operating procedures that you/your agency currently use)**		
• No	149	88.2%
• Yes	20[1]	11.8%
**14. In determining the value/sufficiency of a latent impression, how do you define an impression that is not suitable for individualization but could potentially be used for exclusion? (Given the standard operating procedures that you/your agency currently use)**		
• It has its own category used in standard practice, such as "Of value for exclusion only" or "Limited value"	33	19.5%
• It has its own category, such as "Of value for exclusion only" or "Limited value" - but only used upon request	42	24.9%
• No value	81	47.9%
• Of value	13	7.7%

[1] *Eleven of these were from non-U.S. examiners.*

	Count	%
**15. Do you have a different value threshold for AFIS searches than for non-AFIS casework, such as "AFIS quality" or "of value for AFIS"? (Given the standard operating procedures that you/your agency currently use)**		
• No	53	31.4%
• Yes, used informally	70	41.4%
• Yes, used as formal designation	46	27.2%
**16. Are the latent prints in the White Box Study similar to those that you might encounter in casework?**		
• Some of them	20	11.8%
• Most of them	47	27.8%
• All but one or two	6	3.6%
• All of them	96	56.8%
**17. Are the exemplar prints in the White Box Study similar to those that you might encounter in casework?**		
• Some of them	14	8.3%
• Most of them	45	26.6%
• All but one or two	9	5.3%
• All of them	101	59.8%
**18. Overall, how do the comparisons in the White Box study compare to your casework? In other words, is the distribution of difficulty of the comparisons in the White Box study similar to the distribution of difficulty in your casework?**		
• MUCH EASIER than casework	4	2.4%
• EASIER than casework	11	6.5%
• SIMILAR to casework	128	75.7%
• HARDER than casework	25	14.8%
• MUCH HARDER than casework	1	0.6%
**19. Would you consider using markup such as this for actual casework?**		
• No	36	21.3%
• Possibly	42	24.9%
• Yes, for general practice	39	23.1%
• Yes, Only for complex cases	52	30.8%
**20. If your country or agency has a minimum number of minutiae required to make an individualization (or identification) decision, please indicate that minimum point standard here. (Text response)[2]**		
• No point standard (no response or said they had no point standard for individualization)	150	88.8%
• 16-point standard	2	1.2%
• 12-point standard	11	6.5%
• 10-point standard "for a criminal case"	1	0.6%
• 8-point standard	3	1.8%
• 7-point standard	2	1.2%

[2] *See Appendix SI-18 for discussion of how the point standard text responses were categorized for analysis.*

Appendix SI-8 Associations between examiners' annotations and their determinations

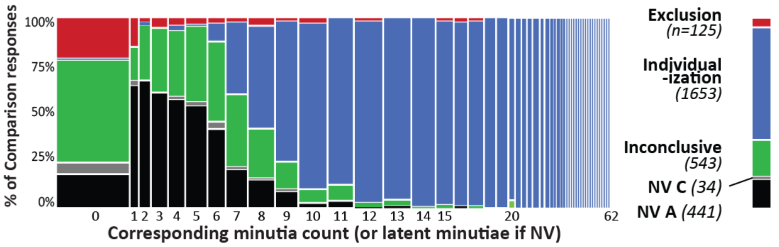

Fig. S3: Associations of corresponding minutia counts and determinations (n=2796 responses by 165 examiners on 231 mated image pairs). This differs from Figure 3B by including the Analysis phase minutia count for NV latents rather than showing zero corresponding minutia count. "NV A" denotes NV in the Analysis phase; "NV C" denotes NV in the Comparison phase.

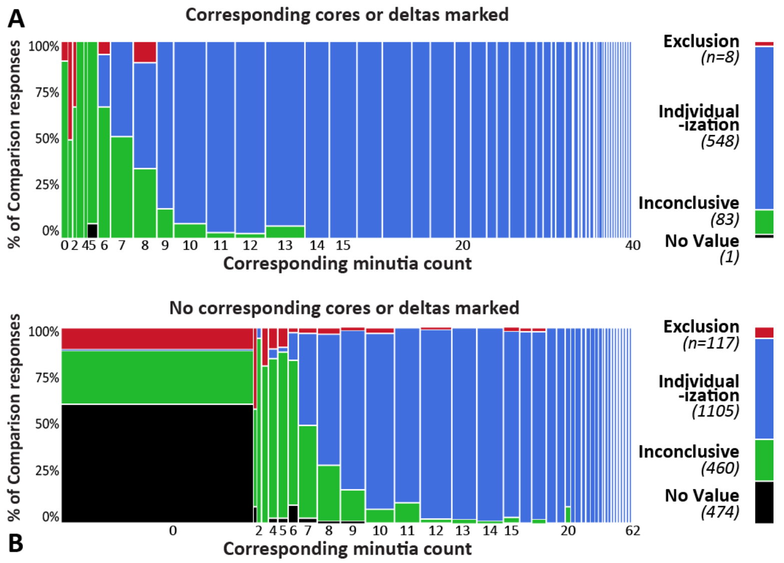

Fig. S4: Associations of corresponding minutia counts and determinations by 165 examiners on 231 mated image pairs, conditioned on (A) at least one corresponding core or delta marked (n=640 responses); and (B) no corresponding cores or deltas marked (n=2156 responses). NV decisions from the Analysis phase are treated as having zero corresponding minutiae.

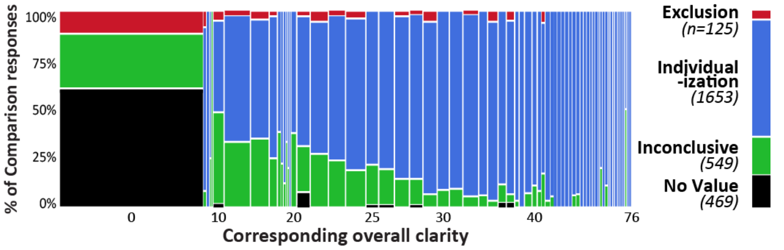

Fig. S5: Associations of corresponding Overall Clarity and determinations (n=2796 responses by 165 examiners on 231 mated image pairs).

Appendix SI-9 Low count individualizations

Table S5 details our review of the individualization determinations with fewer than seven corresponding minutiae. In all of the annotations of image pairs with fewer than five corresponding minutiae marked, examiners marked but did not link (correspond) additional features (improper annotation). Most of the image pairs with five or six corresponding minutiae marked included additional nonminutia features. After discounting the outliers that we believe were due to improper annotation, we did find examples of individualizations with as few as six corresponding minutiae or five minutiae with two level-3 points marked on incipient ridges.

Image Pair	Corresponding Features: Minutiae	Corresponding Features: Cores, Deltas	Corresponding Features: Other Features	Corresponding Features: Debatably Corresponding Features	ID Rate	Median corresponding minutiae among IDs (overall)	Our comments on the annotation
CW083	0	0	0	0	100%	15 (15)	13 apparently corresponding minutiae were marked but not linked
*CW262	0	0	0	1	100%	14 (14)	10 apparently corresponding minutiae were marked but not linked; obvious errors in using software
*CW167	2	0	0	8	100%	19 (19)	a total of 15 apparently corresponding minutiae marked, but obvious errors in using software to link
CW156	4	0	0	0	75%	10 (9.5)	7 apparently corresponding incipient ridge features were marked but not linked (in green clarity area)
CW306	4	0	0	0	64%	10 (9)	corresponding delta & its features not marked (in blue clarity area)
CW173	5	0	2	0	53%	8 (7)	small area of green clarity
CW022	6	0	0	0	55%	9 (7)	3 apparently corresponding minutiae were marked but not linked (in yellow clarity area)
CW081	6	0	4	3	45%	8 (6)	
CW141	6	0	0	0	8%	6 (0)	this examiner made the sole ID for this image pair (out of 12)
CW154	6	0	0	0	79%	9 (7.5)	
CW185	6	1	0	5	64%	13 (12)	
CW201	6	1	0	0	36%	6.5 (5)	
CW201	6	0	0	0	36%	6.5 (5)	core not marked; 3 apparently corresponding minutiae marked (in green clarity area), but not linked
CW218	6	1	0	0	23%	17 (5)	minimal overlap
CW249	6	0	0	3	91%	9.5 (10)	
CW326	6	1	0	0	44%	12.5 (3)	

* Image pair comparisons were by the same examiner.

Table S5: Descriptions of individualizations with fewer than seven corresponding minutiae marked; comments are based on the authors' manual review of the original annotations. In some cases, features were marked in the latent and exemplar that clearly corresponded, but the pairs were not explicitly linked by the examiner. Not linking features could have been an oversight or a deliberate expression of uncertainty. This table excludes five examiners who did not correspond features.

Appendix SI-10 Reproducibility of individualization determinations

Predictors		DF	AICc	R^2	Gen R^2	Misclass.	AUC
None		0	312	0.0000	0.0000	0.3976	0.5000
CD>0		1	303	0.0378	0.0671	0.3976	0.5943
Difficulty		5	255	0.2185	0.3443	0.2632	0.7926
OverallClarity		1	241	0.2379	0.3703	0.2370	0.8143
OverallClarity + CMin		2	223	0.3024	0.4518	0.2088	0.8483
Determination	*{Individualization, Insufficient}*	1	237	0.2492	0.3850	0.2054	0.7819
CMin		1	225	0.2877	0.4339	0.2035	0.8408
CMin_green + CMin_yellow		2	222	0.3037	0.4534	0.2000	0.8497
CMin + Difficulty		6	226	0.3175	0.4699	0.1992	0.8565
Determination + CMin		2	224	0.2981	0.4466	0.1995	0.8428

Table S12: Logistic regression models predicting whether a second examiner will individualize {Indiv, Insuff} based on first examiner's response (n=2671). Constructed from all distinct pairs of examiners on each mated image pair; each image pair weighted equally (n=231).

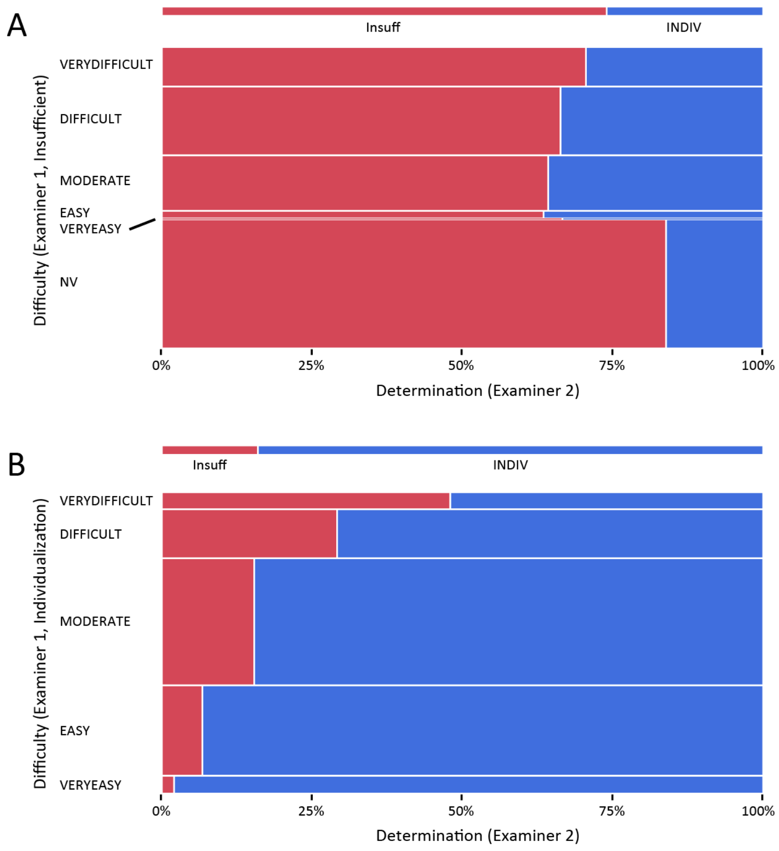

Fig. S19: Reproducibility of sufficiency decisions by difficulty. Mosaic charts showing proportions of examiners who reproduced determinations (x-axis), conditioned on the determination and difficulty assessment of another examiner who determined (A) insufficient or (B) individualization. Chart A: n=1018 insufficient determinations on 168 mated pairs (10,927 paired examiner responses). Chart B: n=1653 individualization determinations on 194 mated pairs (constructed from 18,289 paired examiner responses).

Appendix SI-11 Models of latent value sufficiency

The data on latent value sufficiency contributes to our understanding of sufficiency for individualization in two important ways:

- When modeling sufficiency for individualization, our measures of corresponding clarity and corresponding feature counts were often zero because responses to image pairs were not provided when the latent was determined to be NV. The Analysis phase data is sometimes easier to interpret than the Comparison/Evaluation phase data because it is neither conditioned on a prior response (Analysis determination), nor on the examiner's ability to align the latent and exemplar prints. The associations between Analysis annotations and VID are very similar to the associations between Comparison annotations and individualizations.
- In a previous study [1], we reported similar results for latent value determinations. The data collected in this experiment includes examiner effects (many responses to each latent), a much larger sample of latents that are of borderline value, and more independent variation in latent clarity and minutia counts. These differences allow us to observe additional and more subtle effects. These results generally support our previous findings.

The following analysis is based on logistic regression models describing the association between examiner markup and determinations by the same examiner of latent value for individualization (VID) and value for comparison (VCMP); n=3730 responses to 301 latents by 170 examiners.

Examiners rated most latents VID. Therefore, the percentage of latent determinations that were not VID (35.1%) represents the base misclassification rate (see Table S7). Predicting individual VID determinations based on the majority determination for each latent reduces the misclassification rate to 15.5%. This latter statistic represents a theoretical limit to how well any model based exclusively on properties of the latents can perform on this dataset. This limit is imposed by the lack of agreement among examiners. Both of these reference numbers reflect our deliberate selection of data for the test: we wanted abundant data from the Comparison phase, and we concentrated data selection on borderline cases in order to measure decision thresholds (see Fig. S1A). The corresponding statistics for VCMP determinations are 19.1% base misclassification rate, and 11.3% theoretical limit for models based exclusively on latent characteristics.

A simple model based on the median number of minutia that examiners marked on the latents performs close to the theoretical limits, with misclassification rates of 17.0% (VID) and 13.7% (VCMP). This strong performance leaves little room for improving the model using other descriptors of the latents, such as measures of clarity. Median minutia count, which is a constant for each latent, captures none of the variability due to differences among examiners, interaction effects between examiners and latents, or any other factors contributing to disagreements; it is based exclusively on properties of the latents. Further substantial improvements to the models must account for these other sources of variability.

Using each examiner's minutia count to predict that examiner's latent value determination, results in misclassification rates of 7.3% (VID) and 11.5% (VCMP). The misclassification rate for VID is much lower than the theoretical limit for any metric describing only the latent. That is, individual examiners' counts and value determinations co-vary to an extent beyond what can be explained by properties of the latent.

- Much of the variability (disagreement) in value determinations is associated with variability (disagreement) in minutia counts.
- One implication is that "minutia count" must be understood as a subjective measure (depending on the examiner) and not strictly as a property of the latent.

Examiners' counts are subjective and biased in relation to their determinations. Insofar as this bias is captured in the median minutia count, one might describe the *median* as a "subjective" measure. It is important to understand, however, that this subjectivity pertains to which properties of the latent are actually being measured and does not pertain to variability among examiner responses.

Examiner subjectivity may manifest in various ways, such as a general tendency toward (or from) VID determinations, conformance to a point standard, or tending to mark more (or fewer) features. This experiment was not specifically designed to resolve these questions, but offers some insight.

- The *Latent + Examiner* model gives misclassification rates of 8.2% (VID) and 5.9% (VCMP) [DF=469]. Adding examiners' subjective minutia counts substantially reduces the misclassification rates to 3.0%

(VID) and 2.7% (VCMP). This demonstrates that minutia count captures a substantial proportion of the Latent-Examiner interaction effects (as opposed to merely additive effects due to general examiner tendencies). Any overfitting (due to the 469 degrees of freedom) would bias all four of these estimates downward, potentially giving a misleadingly low indication of the remaining lack of repeatability.

- Individualizations were frequently made with fewer than 12 marked features. As a group, the 10 examiners following a 12-point standard actually rated a slightly greater proportion of latents VID than did other examiners: 157/220 (75%) vs. 2263/3510 (65%) [unconvincing p=0.0381]. We have no evidence that this is causally related to the point standard itself, as this variable is confounded with other effects that were not controlled in the experimental design.
- The mere association between number of minutiae marked and value determinations does not necessarily imply a causal relation. The data appear consistent with various possible explanations. Determinations might methodically follow Analysis phase annotations; however, alternatively, a preliminary determination (possibly subconscious) might influence how an examiner marks a latent.

The following models describing latent value determinations extend our previous work [1]. The fingerprints in this study were selected to include a much higher proportion rated NV or VEO; they were selected to more easily discriminate effects of feature counts, image clarity, complexity, and the presence of cores and deltas. We also obtained multiple examiner responses to each latent in order to investigate subjectivity in examiner determinations. The fingerprints in this study were concentrated more toward those characteristics that would be of borderline sufficiency; this resulted in lower rates of agreement on value determinations than measured in our Black Box study [2]. Examiners were unanimous on whether 118/301 (39.2%) of the latents were VID (23 Not VID; 95 VID), with a mean percentage agreement of 77.5%. Examiners were unanimous on whether 151/301 (50.2%) of the latents were of value for comparison (5 NV; 146 CMP), with a mean percentage agreement of 81.8%.

In the following tables, the Predictors column describes the independent variables used in logistic regression models predicting examiner latent value determinations {VID, Not VID} and {VCMP, NV}. Each model was fit to 3730 responses by 170 examiners on 301 latents. In these models, all terms are additive; asterisks denote cross-product terms expressing interactions between pairs of explanatory variables. Table S7 summarizes explanatory models describing causal relations between the stimuli (in this case, image and examiner) and the response (determination): the models also describe the contribution of fixed attributes of each image such as median minutia count. The models in Table S8 describe associations between examiners' annotations and value determinations (same examiner); these models do not presume causality.

General caution: statistical measures such as the corrected Akaike Information Criterion (AICc), Generalized R^2 and p-values (not shown, but considered in model selection) assume that the 3730 responses are independent. This assumption is not valid because each examiner and image contributed to multiple responses; as a result, these statistics may be substantially biased (to indicate the models are better than they really are). Such biases were considered when selecting models for inclusion in the tables. Dozens of additional variants of these models were fitted and generally yielded similar results. Such models included alternate measures of features and clarity (such as largest contiguous areas at each level of clarity), cross terms and transforms of terms.

**Latent**	Nominal variable identifying the latent (n=301)
**Examiner**	Nominal variable identifying the examiner (n=170)
**MC1, .., MC5**	Areas of red, yellow, green, blue, aqua from median clarity map
**CD_rate**	Continuous variable indicating of the proportion of examiners who marked at least one core or delta (138 voting examiners)
**cdm**	Total number of minutiae, cores, and deltas marked by this examiner
**green_MC**	Area of green or higher clarity from median clarity map = AA3 + AA4 + AA5
**green_MC_LCA**	Largest contiguous area of green or higher clarity from median clarity map
**Min_green**	Minutiae that this examiner annotated as green or higher clarity
**Min_green_MC**	Minutiae of this examiner in green or higher clarity from median clarity map
**green**	Area of green or higher clarity annotated by this examiner = A3 + A4 + A5
**MedianMin**	Median(Min) across all responses to the same latent
**Min**	Minutia count for the latent print
**OC**	Overall Clarity from this examiner's annotation
**OC(MC)**	Overall Clarity from median clarity map
**PtStd**	Whether the examiner followed a 12-point standard
**YY**	Area of yellow or higher clarity from median clarity map = AA2 + AA3 + AA4 + AA5

Table S6: Legend of variables used to predict latent value determinations.

In these tables, DF= degrees of freedom; R^2 = entropy R^2; AICc = corrected Akaike Information Criterion (AICc); Gen R^2=Generalized R^2; Misclass= misclassification rate; AUC = area under the (receiver operating characteristic) curve.

Predictors	DF	VID					VCMP				
		AICc	R^2	Gen R^2	Misclass	AUC	AICc	R^2	Gen R^2	Misclass	AUC
None (base rate)	0	4838	0.0000	0.0000	0.3512	0.5000	3642	0.0000	0.0000	0.1912	0.5000
Examiner	169	4769	0.0874	0.1474	0.3201	0.6923	3593	0.1108	0.1644	0.1906	0.7245
Latent	300	3005	0.5141	0.6696	0.1547	0.9278	2458	0.5046	0.6240	0.1134	0.9349
Latent; Examiner	469	2447	0.7164	0.8327	0.0815	0.9772	2079	0.7245	0.8134	0.0590	0.9820
CD_rate	1	4561	0.0576	0.0990	0.3619	0.6596	3176	0.1285	0.1891	0.1912	0.7450
CD_rate; Examiner	170	4458	0.1521	0.2464	0.2960	0.7564	3099	0.2470	0.3437	0.1788	0.8363
MC1; MC2; MC3; MC4; MC5	5	3475	0.2838	0.4237	0.2080	0.8474	2657	0.2732	0.3756	0.1657	0.8543
green_MC	1	3506	0.2757	0.4137	0.2169	0.8444	2730	0.2511	0.3488	0.1818	0.8395
log(YY); green_MC; log(YY)*green_MC	3	3326	0.3139	0.4601	0.2105	0.8562	2607	0.2858	0.3906	0.1595	0.8564
green_MC_LCA	1	3469	0.2835	0.4233	0.2113	0.8476	2722	0.2532	0.3513	0.1826	0.8397
OC(MC)	1	3427	0.2920	0.4338	0.2121	0.8506	2682	0.2643	0.3648	0.1708	0.8503
OC(MC); Examiner	170	3171	0.4184	0.5763	0.1759	0.8993	2526	0.4045	0.5234	0.1332	0.9054
MedianMin	1	2784	0.4250	0.5831	0.1697	0.9014	2239	0.3860	0.5037	0.1367	0.8988
MedianMin; CD_rate	2	2785	0.4252	0.5833	0.1697	0.9012	2132	0.4159	0.5353	0.1196	0.9079
MedianMin; PtStd	2	2798	0.4265	0.5846	0.1697	0.9019	2240	0.3861	0.5038	0.1357	0.8989
MedianMin; OC(MC)	2	2753	0.4318	0.5901	0.1705	0.9042	2222	0.3912	0.5093	0.1351	0.9012
MedianMin; Median(Min_green_MC)	2	2735	0.4356	0.5939	0.1702	0.9061	2232	0.3885	0.5063	0.1386	0.9002
MedianMin; YY; green_MC	3	2739	0.4353	0.5936	0.1681	0.9063	2232	0.3890	0.5069	0.1351	0.9007
MedianMin; Examiner	170	2355	0.5872	0.7335	0.1142	0.9517	1950	0.5626	0.6780	0.0909	0.9522

Table S7: Latent value determination as a dependent response to (A) the image pairs and examiners; (B) attributes of the image pairs as estimated by median statistics (n=3730). These models are intended to address questions of causality and therefore do not include same-examiner associations between the predictor variables and the determinations. Predictors such as MedianMin do not vary by image and therefore describe something about the image itself (albeit something about examiners' collective responses to the image).

Predictors	DF	VID					VCMP				
		AICc	R^2	Gen R^2	Misclass	AUC	AICc	R^2	Gen R^2	Misclass	AUC
None (base rate)	0	4838	0.0000	0.0000	0.3512	0.5000	3642	0.0000	0.0000	0.1912	0.5000
CD	1	4717	0.0254	0.0445	0.3512	0.5873	3328	0.0867	0.1302	0.1912	0.6571
CD; Examiner	170	4631	0.1163	0.1927	0.3048	0.7252	3289	0.1948	0.2779	0.1863	0.8004
cdm; points	2	2088	0.5694	0.7185	0.1134	0.9463	1813	0.5036	0.6230	0.1099	0.9409
green	1	3828	0.2091	0.3268	0.2466	0.8052	2879	0.2102	0.2976	0.1912	0.8100
OC	1	3771	0.2211	0.3430	0.2504	0.8148	2867	0.2134	0.3016	0.1858	0.8228
OC; CD_rate	2	3607	0.2553	0.3879	0.2255	0.8316	2491	0.3172	0.4272	0.1552	0.8724
OC; Examiner	170	3408	0.3693	0.5237	0.1834	0.8836	2673	0.3642	0.4800	0.1440	0.8908
Min	1	2044	0.5782	0.7260	0.1150	0.9486	1895	0.4804	0.6005	0.1147	0.9348
Min; CD_rate	2	2027	0.5821	0.7293	0.1126	0.9499	1686	0.5385	0.6560	0.0962	0.9476
Min; Examiner	170	1672	0.7284	0.8411	0.0617	0.9793	1538	0.6758	0.7749	0.0617	0.9752
Min; Examiner; (Examiner*Min)	339	1661	0.8114	0.8957	0.0601	0.9891	1630	0.7577	0.8386	0.0598	0.9854
Min; Examiner; CD_rate	171	1654	0.7325	0.8439	0.0598	0.9799	1312	0.7386	0.8243	0.0469	0.9839
Min; Min_green	2	1985	0.5908	0.7365	0.1083	0.9526	1871	0.4877	0.6077	0.1150	0.9363
Min; Latent	301	2149	0.6916	0.8149	0.0845	0.9736	1956	0.6431	0.7480	0.0783	0.9694
Min; Latent; Examiner	470	770	0.8570	0.9233	0.0303	0.9946	1596	0.8577	0.9099	0.0271	0.9954
Min; MedianMin	2	1950	0.5979	0.7424	0.1080	0.9531	1812	0.5039	0.6233	0.1099	0.9406
Min; OC	2	1974	0.5930	0.7384	0.1064	0.9527	1846	0.4945	0.6144	0.1102	0.9382
Min; OC(MC)	2	1959	0.5961	0.7409	0.1054	0.9529	1844	0.4951	0.6149	0.1110	0.9383
Min; PtStd	2	2012	0.5852	0.7319	0.1126	0.9502	1854	0.4922	0.6121	0.1107	0.9380

Table S8: Logistic regression models describing associations between latent annotation and value determinations made by the same examiner (n=3730). These models describe associations between examiners' annotation and determination responses to the latents. Dozens of additional variants of these models were fitted and generally yielded similar results.

1 Ulery B, Hicklin R, Kiebuzinski G, Roberts M, Buscaglia J (2013) Understanding the sufficiency of information for latent fingerprint value determinations. *Forensic Sci Int* **230(1)**:99-106. (http://www.noblis.org/media/3c760709-5971-4efe-8edf-f00435fcdf1b/docs/article_understanding_sufficiency_information_latent_pdf)

2 Ulery BT, Hicklin RA, Buscaglia J, Roberts MA (2011) Accuracy and reliability of forensic latent fingerprint decisions. Proc Natl Acad Sci USA 108(19): 7733-7738. (http://www.pnas.org/content/108/19/7733.full.pdf)

Appendix SI-12 Models of sufficiency for individualization

The following tables evaluate logistic regression models describing associations between examiner markup and examiner determinations {Individualization, Insufficient}; insufficient includes both inconclusive and no value determinations. Each model was fit to the 2671 valid responses on mated pairs (which omit 125 false exclusions). In these models, all terms are additive; asterisks denote cross-product terms expressing interactions between pairs of explanatory variables.

The base rate in these models is the percentage of the 2671 mated pairs that were ***not*** individualized (1018 were NV or inconclusive); i.e., a trivial model that assumes examiners will always individualize mated pairs would have a 38.1% misclassification rate.

Statistical measures such as AICc and Generalized R^2 unrealistically assume that responses are independent. Each examiner and image contributed to multiple responses. Consequently, these statistics may be substantially biased (and indicate the models are better than they really are). Such biases were considered when selecting models for inclusion in the tables.

**ImagePair**	Nominal variable identifying the mated pair (n=231)
**Examiner**	Nominal variable identifying the examiner (n=165)
**A1, A2, A3, A4, A5**	Area of red, yellow, green, blue, aqua from this examiner's corresponding clarity map
**any_CDMP**	Number of corresponding and debatably corresponding features marked by this examiner (Cores, Deltas, Minutiae, and Points)
**any_CMin**	Number of corresponding and debatably corresponding minutiae marked by this examiner(=CMin+deb_CMin)
**CD_rate**	Continuous voted metric indicating presence of core or delta (107 voting examiners)
**CDMP**	Number of corresponding features marked by this examiner (Cores, Deltas, Minutiae, and Points)
**CMin**	Number of corresponding minutiae marked by this examiner (Analysis phase NV responses were counted as having 0 corresponding minutiae)
**CMin0, …, CMin5**	Number of corresponding minutiae marked in black, …, aqua clarity regions
**complexity**	Determined during preliminary screening, based on distortion, background or processing
**deb_CMin**	Debatably corresponding minutiae as marked by this examiner
**deb_CMin_green**	Debatably corresponding minutiae as marked by this examiner annotated as green (or higher) clarity
**deb_CMin_yellow**	Debatably corresponding minutiae as marked by this examiner annotated as yellow (or lower) clarity
**CMin_green**	Corresponding minutiae that this examiner annotated as green (or higher) clarity (=CMin3 + CMin4 + CMin5)
**CMin_yellow**	Corresponding minutiae that this examiner annotated as yellow (or lower) clarity (=CMin0 + CMin1 + CMin2)
**Has_CC_map**	examiner marked at least 3 corresponding or debatable features
**Marked_CD**	Nominal metric indicating whether this examiner marked any cores or deltas
**Marked_CMin**	Whether any corresponding minutiae were marked (nominal variable)
**Mean(CMin)**	Mean(corresponding minutiae) among all examiners
**Median(CMin)**	Median(corresponding minutiae) among all examiners
**MedianOverallClarity**	Overall Clarity from median clarity map (calculated among all examiners)
**OverallClarity**	Overall Clarity from this examiner's corresponding clarity map (compare to MedianOverallClarity)
**otherPts**	Number of corresponding "other" features (other than minutiae, cores, and deltas) that were marked by this examiner
**PtStd**	whether examiner followed a 12-point standard
**VCMP**	latent rated VEO or VID
**VID**	latent rated VID

Table S9: Legend of predictor terms

In the following tables, DF = degrees of freedom; R^2 = entropy R^2; AICc = corrected Akaike Information Criterion; Gen R^2 = Generalized R^2; Misclass = misclassification rate; AUC = area under the (receiver operating characteristic) curve.

Predictors	DF	AICc	R^2	Gen R^2	Misclass	AUC
None (base rate)	0	3552	0.0000	0.0000	0.3811	0.5000
ImagePair	230	3295	0.5854	0.7354	0.1303	0.9458
Examiner	164	3546	0.1004	0.1699	0.3280	0.7024
ImagePair; Examiner	394	1696	0.7837	0.8801	0.0629	0.9865
complexity	1	3518	0.0103	0.0185	0.3811	0.5600
Median(Difficulty)	1	3407	0.0414	0.0728	0.2493	0.5225
CD_rate	1	3053	0.1413	0.2328	0.3164	0.7399
Median clarity A1; A2; A3; A4; A5	5	2764	0.2250	0.3515	0.2396	0.8179
CD_rate; MedianOverallClarity	2	2479	0.3033	0.4513	0.2314	0.8457
Median(CMin)	1	1806	0.4925	0.6533	0.1355	0.9219
Median(CMin); CD_rate	2	1808	0.4925	0.6533	0.1355	0.9216
Median(CMin); MedianOverallClarity	2	1786	0.4985	0.6589	0.1359	0.9252
Mean(CMin)	1	1751	0.5079	0.6676	0.1449	0.9298
Median(CMin); Examiner	165	1577	0.6555	0.7909	0.0947	0.9657
Median(CMin); Examiner; (Median(CMin) * Examiner)	329	1700	0.7333	0.8468	0.0940	0.9787

Table S10: Individualization determination {Individualization, Insufficient} as a dependent response to (A) image pairs and examiners; (B) attributes of the image pairs as estimated by median statistics (n=2671). These models are intended to address questions of causality and therefore do not include same-examiner associations between the predictor variables and the determinations. Predictors such as Median(CMin) do not vary by image and therefore describe attributes of the images themselves (albeit based on examiners' responses to the image).

Fig. S18 reveals the goodness of fit of the *Median(CMin)* model from Table S10.

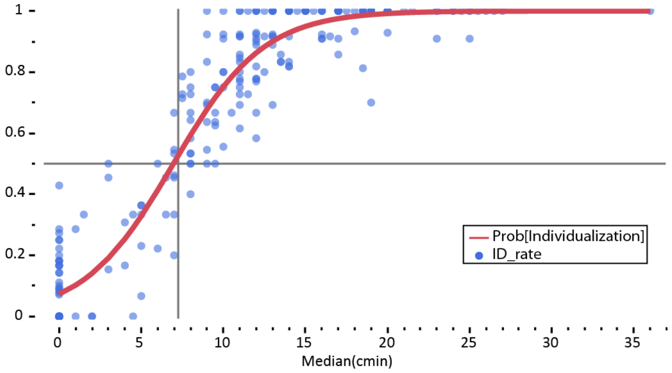

Fig. S18: Overlay plot showing the fitted logistic model for Median(CMin) against a scatterplot of the individualization rates for 231 mated image pairs. The cross-hairs indicate how the misclassification rate is calculated based on whether the probability estimate from the fitted model exceeds 50%.

Predictors	DF	AICc	R^2	Gen R^2	Misclass	AUC
None (base rate)	0	3552	0.0000	0.0000	0.3811	0.5000
Marked_CD	1	3312	0.0684	0.1181	0.3811	0.6245
VCMP	1	2477	0.3034	0.4513	0.2055	0.7304
A1; A2; A3; A4; A5	5	2184	0.3883	0.5483	0.1617	0.8977
Difficulty	5	2148	0.3983	0.5590	0.2014	0.8643
A1; A2; A3; A4; A5; CD_rate; complexity	7	2081	0.4184	0.5802	0.1569	0.9063
Has_CC_map	1	1992	0.4401	0.6023	0.1355	0.8252
OverallClarity	1	1976	0.4446	0.6068	0.1711	0.9092
Marked_CMin	1	1834	0.4846	0.6458	0.1262	0.8349
OverallClarity; Has_CC_map	2	1796	0.4959	0.6564	0.1382	0.9092
Has_CC_map; A1; A2; A3; A4; A5	6	1798	0.4976	0.6581	0.1359	0.9060
VID	1	1596	0.5517	0.7068	0.1041	0.8635
any_CDMP	1	1092	0.6936	0.8191	0.0786	0.9695
any_CMin	1	1084	0.6959	0.8207	0.0738	0.9698
CDMP	1	870	0.7560	0.8621	0.0592	0.9803
cdm; otherPts	2	872	0.7561	0.8621	0.0592	0.9804
CMin	1	862	0.7584	0.8637	0.0595	0.9806
CMin; CD	2	864	0.7584	0.8637	0.0595	0.9807
CD_rate; CMin	2	862	0.7590	0.8641	0.0577	0.9813
CMin; OverallClarity	2	855	0.7609	0.8654	0.0580	0.9811
CMin0; CMin1; CMin2; CMin3; CMin4; CMin5	6	858	0.7623	0.8663	0.0573	0.9817
CMin; PtStd	2	848	0.7628	0.8665	0.0573	0.9812
CMin; A1; A2; A3; A4; A5	6	839	0.7677	0.8698	0.0577	0.9821
CMin_green; CMin_yellow	2	854	0.7611	0.8655	0.0595	0.9814
CMin_green; CMin_yellow; (CMin_green*CMin_yellow)	3	851	0.7625	0.8664	0.0547	0.9812
CMin_green; CMin_yellow; deb_CMin_green; deb_CMin_yellow	4	857	0.7614	0.8657	0.0562	0.9813
CMin_green; CMin_yellow; g_point; y_point	4	846	0.7647	0.8678	0.0588	0.9820
CMin_green; CMin_yellow *[from median clarity map]* [§]	2	825	0.7693	0.8708	0.0539	0.9825
CMin0; CMin1; CMin2; CMin3; CMin4; CMin5 *[from median clarity map]* [§]	6	829	0.7705	0.8716	0.0539	0.9828
CMin; Difficulty (ordinal)	6	799	0.7790	0.8771	0.0539	0.9842
CMin; examiner	165	827	0.8668	0.9303	0.0296	0.9943

[§] *These two models use the median clarity map (generated across multiple examiners) to describe the clarity of the images where this examiner marked the minutiae.*

Table S11: Logistic regression models describing associations between annotations and determinations {Individualization, Insufficient} made by the same examiner (n=2671). These models describe associations between examiners' annotation and determination responses to the image pairs. Dozens of additional variants of these models were fitted and generally yielded similar results. Such models included alternate measures of features and clarity (such as largest contiguous areas at each level of clarity), cross terms and transforms of terms.

Appendix SI-13 Repeatability of minutia counts (Analysis phase)

Differences in minutia counts understate the variability among examiners: annotations may have similar minutia counts but differ greatly in which specific minutiae were marked. Some of the variability in minutia selection may be due to the examiners themselves not being consistent in their minutia selection. In this study, there were 13 instances in which latents were presented twice to the same examiner, once in a mated pair, once in a nonmated pair (Table S4). The examiners were permitted to review their previous work, so they could have compared their responses if they noticed the duplication during the Analysis phase. A manual review of these annotations was conducted to determine whether the same features were marked in the two presentations. One latent (#1) was presented to two different examiners (twice each) and neither examiner marked any features. However, for none of the other 11 latents did the examiner mark the same set of features both times, and value determinations differed in three cases. This small sample of data suggests that much of the lack of interexaminer reproducibility of minutiae could be explained by a lack of intraexaminer repeatability; this echoes our previous findings with examiners' determinations [1].

Latent	Value A	Features A	Value B	Features B	Features in common
1	NV	0	NV	0	-
1	NV	0	NV	0	-
2	NV	2+D	VEO	3+D	2+D
3	VID	9	VID	9 + C	8
4	VID	7+D	VID	8+D	6+D
5	VID	7+C+D	VID	8+C+D	5+C+D
6	VID	11	VID	11+C+D	10
7	VID	11	VID	11	8
8	VID	21	VID	22+C	17
9	VID	7+C+D	VEO	7+D	4+D
10	VID	10+1 other	VID	15	8
11	VID	11+C	VEO	4	4
12	VID	9	VID	12+5 others	8

Table S4: Analysis phase responses for 13 latents that were presented twice to the same examiner. "A" and "B" in the column headers refer to the two presentations of each print. "Features" columns indicate minutiae (total count), cores (C), deltas (D), and "other" points marked. The column "Features in common" counts the number of features marked in both presentation A and presentation B (including several cases where the placement shifted to an adjacent ridge).

1 Ulery BT, Hicklin RA, Buscaglia J, Roberts MA (2012), Repeatability and Reproducibility of Decisions by Latent Fingerprint Examiners. PLoS ONE 7:3. (http://www.plosone.org/article/info:doi/10.1371/journal.pone.0032800)

Appendix SI-14 Reproducibility of determinations by median corresponding minutia count

In the Black Box study [1,2], we described reproducibility of determinations as overall averages for large datasets. In this study, we are able to refine our description of reproducibility as a function of examiner descriptions of specific images. We use percentage agreement to describe reproducibility of determinations. This commonly used statistic simply describes the proportion of times paired responses (different examiners on the same image pair) are in agreement. Percentage agreement on the $\boldsymbol{i}^{th}$ image pair is defined as

$$P_i = \frac{1}{n(n-1)} \sum_{j=1}^{k} n_{ij}(n_{ij} - 1)$$

where $\boldsymbol{n}$ is the number of examiners assigned the image pair, $\boldsymbol{k}$ is the number of determination categories, and $\boldsymbol{n_{ij}}$ is the number of responses assigning the $\boldsymbol{i}^{th}$ image pair to the $\boldsymbol{j}^{th}$ determination category. k=2 for agreement on {Individualization, Not Individualization} or k=3 for {Individualization, Exclusion, Inconclusive or NV}. For 3-way agreement, percentage agreement implicitly treats all disagreements as being equally serious. So, for example, the disagreement "individualization vs. exclusion" is not weighted differently than the disagreement "individualization vs. inconclusive."

Fig. S12 and Table S3 show the percentage agreement among examiners on mated comparison conclusions as a function of the median number of corresponding minutiae marked by all examiners. Percentage agreement was approximately 50% when the median was near 7 to 9 corresponding minutiae, and was below 90% for all categories except when the median was above 16 minutiae.

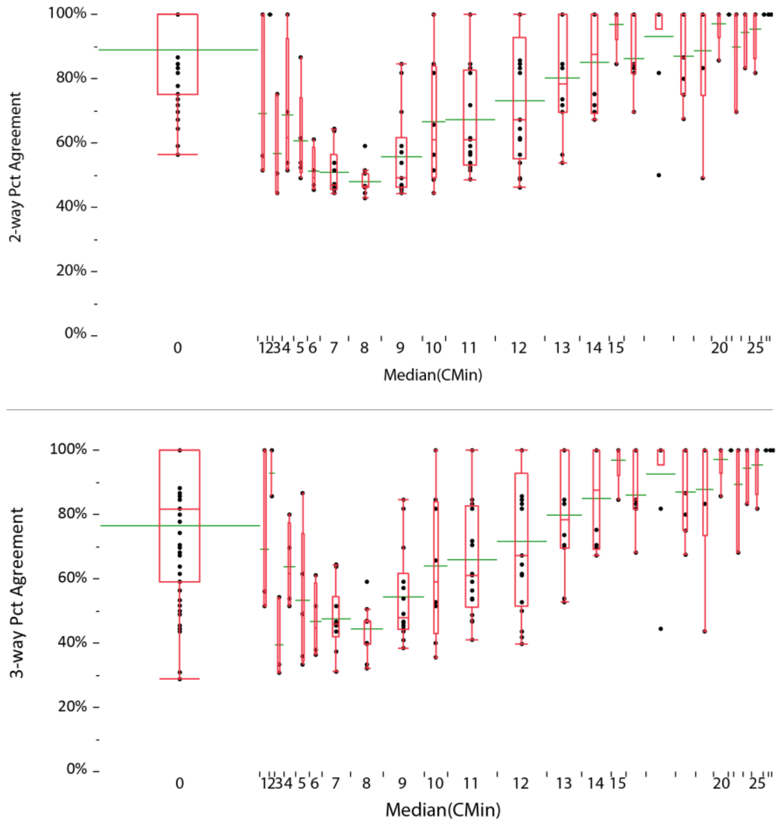

Fig. S12: Percentage agreement by median corresponding minutia count (n=2796 responses by 165 examiners to 231 mated image pairs). Fractional medians are rounded down.

Median(CMin)	N	Mean 2-way Pct Agreement	Mean 3-way Pct Agreement
0	55	89.0%	76.5%
1-3.5	8	72.2%	64.0%
4-6.5	13	60.3%	54.5%
7-9.5	35	51.9%	49.3%
10-12.5	42	69.5%	67.9%
13-15.5	27	85.1%	84.9%
16+	51	92.1%	91.8%
Overall	231	77.9%	73.5%

Table S3: Percentage agreement by median corresponding minutia count (n=2796 responses by 165 examiners to 231 mated image pairs).

1 Ulery BT, Hicklin RA, Buscaglia J, Roberts MA (2011) Accuracy and reliability of forensic latent fingerprint decisions. Proc Natl Acad Sci USA 108(19): 7733-7738. (http://www.pnas.org/content/108/19/7733.full.pdf)

2 Ulery BT, Hicklin RA, Buscaglia J, Roberts MA (2012), Repeatability and Reproducibility of Decisions by Latent Fingerprint Examiners. PLoS ONE 7:3. (http://www.plosone.org/article/info:doi/10.1371/journal.pone.0032800)

Appendix SI-15 Reproducibility of responses

Fig. S6 shows associations between examiners' markup and their determinations, and also interexaminer variability from the Analysis phase. These patterns are similar to those from the Comparison/Evaluation phase shown in Figure 5. Counts of zero minutiae are much more common in the corresponding minutia counts shown in Figure 5 than in the latent print minutia counts shown in Fig. S6 in part because Figure 5 counts image pairs whose latents were determined NV and not compared as having zero corresponding minutiae. For mated pairs, zero corresponding minutiae are likely to be marked for inconclusive determinations if the examiner fails to see the proper alignment of the latent to the exemplar.

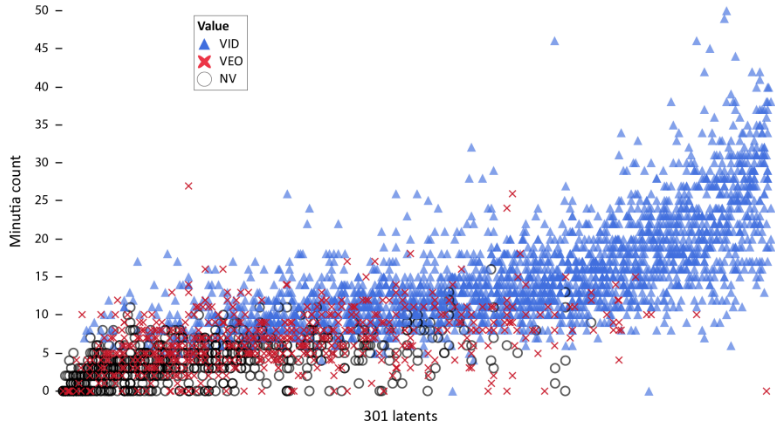

Fig. S6: Individual examiner latent print minutia counts (y-axis) and value determinations (color) by latent print (x-axis, sorted by mean minutia count); n=3730 responses by 170 examiners to 301 latents; 11 points representing minutia counts > 50 not shown (10 of which were for a single latent).

Fig. S7 is similar to Figure 5, but the x-axis is scaled to show how the mean number of corresponding minutiae relates to comparison determinations.

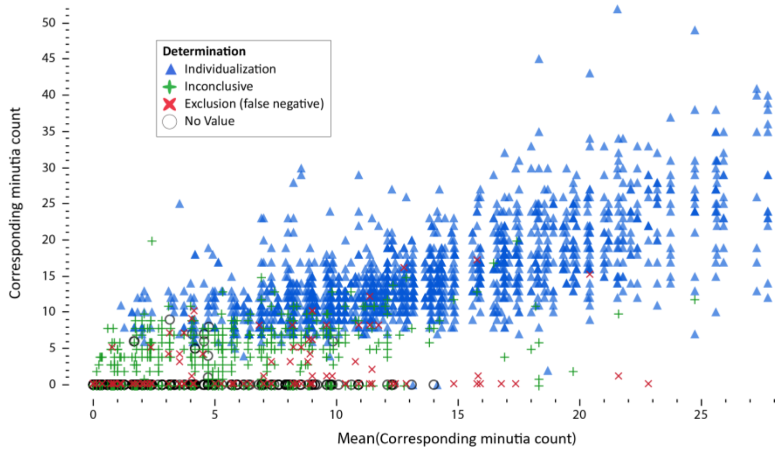

Fig. S7: Corresponding minutia counts (y-axis) and determinations (color) by mean corresponding minutia count (x-axis); n=2796 responses by 165 examiners to 231 mated image pairs. This chart plots the same data as Figure 5, but by mean count instead of by image pair.

Fig. S8, differs from Figure 5 by plotting NV determinations using the number of minutiae marked in the latent; Figure 5 represents those image pairs as having zero corresponding minutiae.

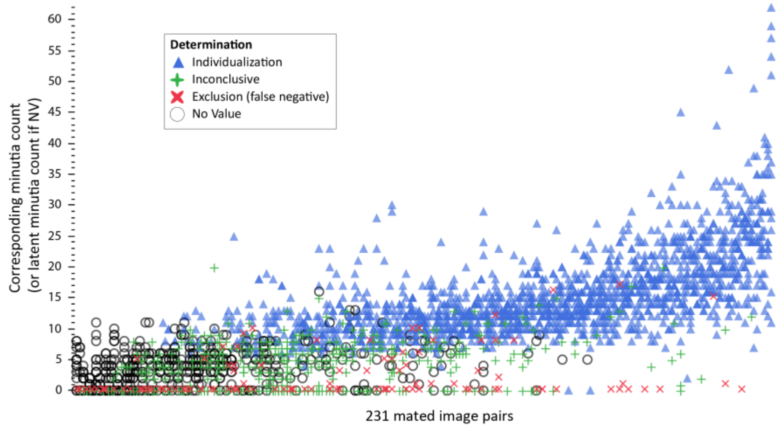

Fig. S8: Corresponding minutia counts (y-axis) and comparison determinations (color) by image pair (x-axis, sorted by mean corresponding minutia count); n=2796 responses by 165 examiners to 231 mated image pairs. This chart plots the same data as Figure 5, except that instead of reporting zero corresponding minutiae for NV determinations, the latent minutia count is reported.

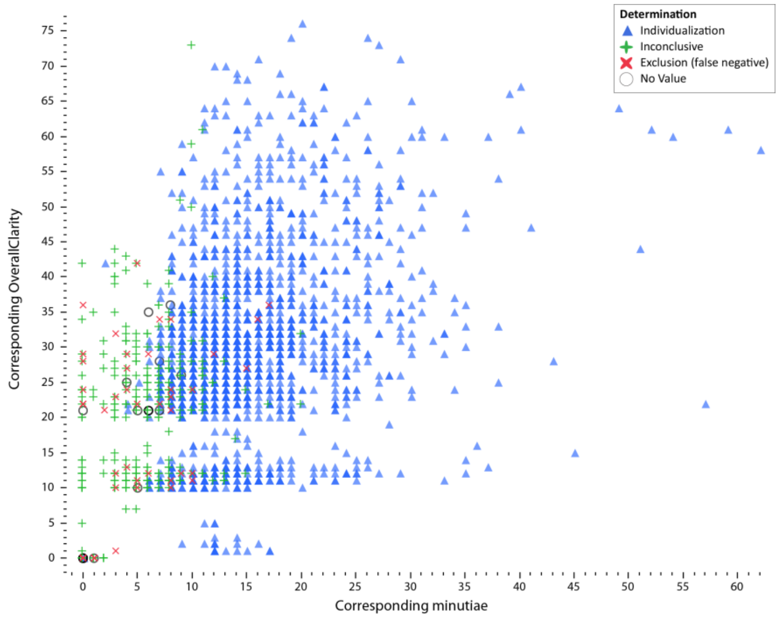

Fig. S9: Examiner determinations in relation to their corresponding minutia counts and OverallClarity as measured on the corresponding clarity map (n=2796 responses by 165 examiners on 231 mated pairs).

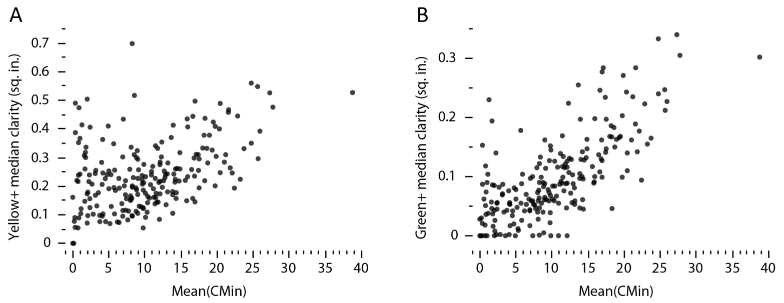

Fig. S10: Associations between corresponding minutia counts and clarity on 231 mated image pairs. Median corresponding minutia count (x-axis) by area in square inches on the median clarity maps that were marked (A) yellow or higher clarity, and (B) green or higher clarity (y-axis).

Appendix SI-16 Corresponding minutia counts and determinations for nonmated image pairs

Fig. S11 shows the corresponding minutia counts associated with determinations and their reproducibility among examiners for nonmated image pairs; see Figure 5 for the equivalent chart for mated image pairs. Among the nonmated image pairs, 89% of the annotations had no corresponding minutiae marked, and few had more than seven corresponding minutiae marked. The single erroneous individualization (false positive) had 14 corresponding minutiae marked (the highest count among 582 comparisons of nonmated pairs).

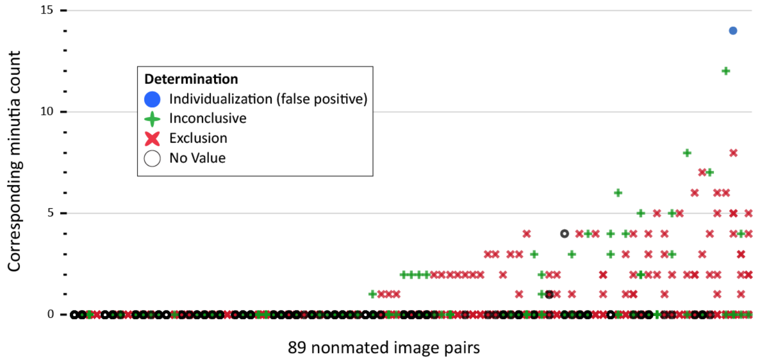

Fig. S11: Corresponding minutia count (y-axis) and determination (color) by nonmated image pair (x-axis). X-axis is sorted by median, then by mean corresponding minutia count. (n=847 responses by 165 examiners to 89 nonmated image pairs, mean of 9.5 responses per image pair; one invalid response omitted)

Appendix SI-17 Estimating repeatability of determinations

In [1] we measured interexaminer percentage agreement on mated pairs to be 86.6% and intraexaminer percentage agreement to be 92.2%. We also observed a proportional relation between repeatability and reproducibility. If we assume a similar relation holds here and that these numbers are driven by the proportion of borderline sufficiency image pairs in the test, then we can estimate a repeatability rate for this study. Based on the observed interexaminer percentage agreement of 79.5% in this study, this simple model predicts an intraexaminer percentage agreement of 88%, which would correspond to a misclassification rate of about 6% (i.e., approximately (1-0.88)/2).

In Table 5, we showed that the *Median(CMin) + Examiner* model resulted in a misclassification rate of 9.5%, most of which can be attributed to the lack of repeatability of individualization determinations.

1 Ulery BT, Hicklin RA, Buscaglia J, Roberts MA (2012), Repeatability and Reproducibility of Decisions by Latent Fingerprint Examiners. *PLoS ONE* **7**:3. (http://www.plosone.org/article/info:doi/10.1371/journal.pone.0032800)

Appendix SI-18 Effect of point standard

In the participant survey (Appendix SI-7, question #20), 19 participants self-identified as having point standards. The free-text responses were sometimes ambiguous. Four examiners specified a seven or eight point standard; given that nearly all of the individualizations had at least seven corresponding minutiae, such a standard presumably would have had negligible influence on the results of this test. Six of the examiners who specified a point count did not conform to that standard in the test, individualizing two or more times with fewer total features annotated. Therefore, for the purposes of analysis, ten participants were identified as having and following a standard: all ten had 12-point standards; most of these were from a single country, none from the U.S..

As was noted in Figure 8, one effect of a point standard is that it has a substantial effect on the number of minutia marked. For the examiners with a 12-point standard, Fig. S13B shows the strikingly disproportionate number of latents with 12 minutiae marked in Analysis. For the examiners without point count standards, we saw an abrupt step at seven corresponding minutiae in Figure 8A; a similar step is not clearly evident in Fig. S13A.

A: B:

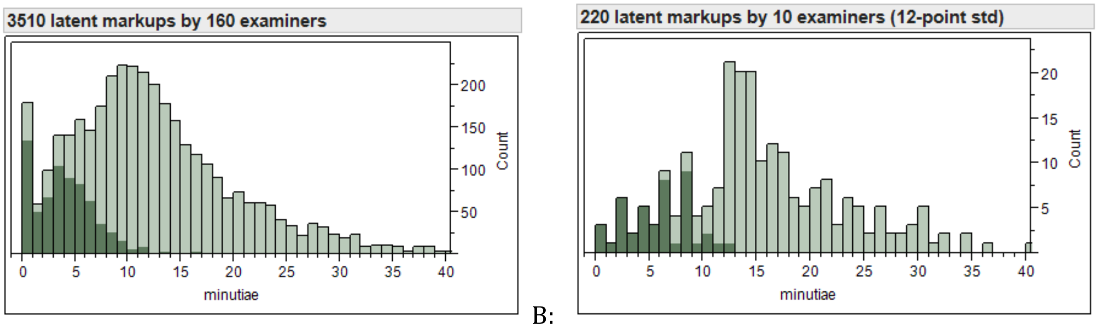

Fig. S13: Effect of point count standards on minutia count. (A) Analysis phase minutia counts by 160 examiners without point count standards; (B) Analysis phase minutia counts by 10 examiners who followed a 12-point standard. NV determinations shaded; plots truncated at 40 minutiae.

Appendix SI-19 Minimum number of corresponding minutiae

The minimum number of corresponding minutiae that each examiner reported when individualizing varied across examiners as shown in Fig. S14. We investigated the reasons for this wide variation in the data and what it tells us about meaningful differences among examiners as opposed to artifacts of how the data was collected. Specifically, we investigated four sources of variation in the data:

- outliers that are not indicative of extreme decision criteria (discussed in Appendix SI-9);
- random variations due to small sample sizes;
- variations associated with differing individualization rates from examiner to examiner; and
- variations associated with subjective differences in marking minutiae.

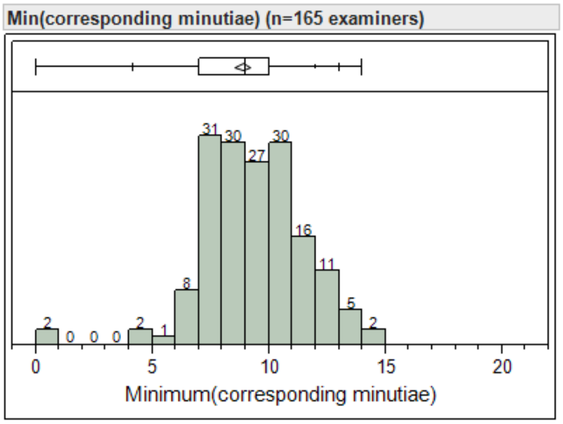

Fig. S14: Distribution of Minimum(corresponding minutiae) marked by examiners when they individualized (n=165).

In order to understand the substantial dispersion in Fig. S14, we performed three simulations to isolate contributing factors.

The minimum is an extreme statistic and biased upwards: if each examiner had been assigned many more comparisons, the minimum count for some examiners would have been lower. In the first simulation (Fig. S15), we assume there are no examiner differences in the number of corresponding minutiae reported, but that there are real differences in the numbers of individualizations per examiner. That is, the simulated examiners differ in sample sizes (up to 17 individualizations per examiner) but not in reported minutia counts. This simulation demonstrates that most of the observed variation is a random effect associated with the small sample sizes. Each simulation run shows nearly as much dispersion as the actual data even though the counts were randomly assigned to simulated examiners. We conclude that most of the dispersion in Fig. S14 is a consequence of the limited number of measurements obtained per examiner.

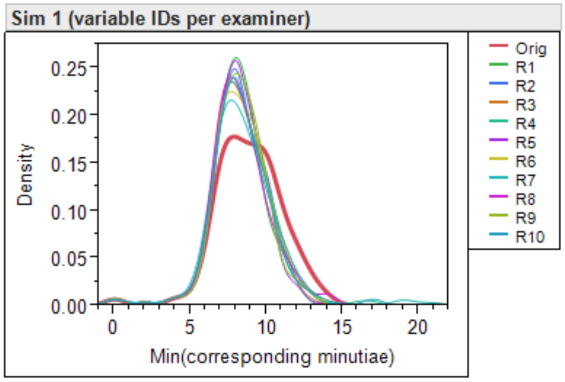

Fig. S15: Simulation #1: variable number of individualizations per examiner. Ten simulation runs in which the actual corresponding minutia counts were randomly assigned to 165 simulated examiners; the number of individualizations per simulated examiner matches the actual distribution of individualizations per examiner. In each run, the random reassignments were performed by permuting the original counts of the 1653 individualizations. The (bold) reference line shows the actual distribution of Minimum(corresponding minutiae). Data are summarized as smoothed kernel density estimates. This simulation demonstrates that most of the observed interexaminer variation in minimum number of corresponding minutiae is a random effect associated with the small sample size.

Next we investigated how much the variable number of individualizations per examiner contributed to the dispersion. In this simulation (Fig. S16), we assume there are no examiner differences in the number of corresponding minutiae reported or in individualization rates. Comparing Fig. S15 and Fig. S16, we see that the varying number of individualizations per simulated examiner had a very minor effect on the distribution. As demonstrated via the logistic regression models (Table 5A), there are important real differences among examiners' individualization rates (more than can be explained by the random test assignments). Fig. S16 demonstrates that these real differences in individualization rates, although they have great effect on the sample size from which the extreme statistic, minimum(corresponding minutiae), was calculated, contribute very little to the dispersion in Fig. S14.

The first simulation (Fig. S15) shows that the actual sample distribution differs substantially from the simulations. The second simulation (Fig. S16) shows that these differences are not due to differences in individualization rates. This means that there are real examiner differences in the number of corresponding minutiae that examiners mark when individualizing. Some examiners will not make individualization determinations if they only mark 7 or 8 corresponding minutiae.

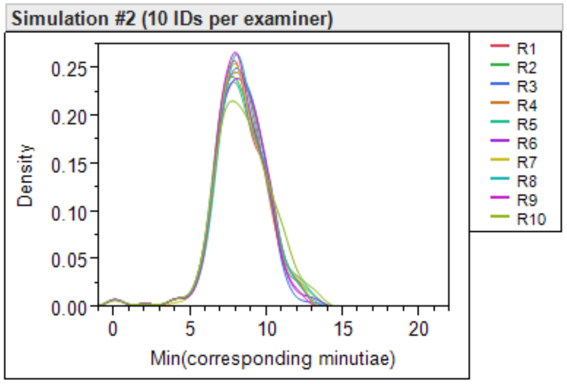

Fig. S16: Simulation #2: constant number of individualizations per examiner. Ten simulation runs in which the actual corresponding minutia counts were randomly assigned to 165 simulated examiners; each simulated examiner made 10 individualizations. In each run, the random reassignments were performed by permuting the original counts of the 1653 individualizations. The random reassignments were performed by permuting the original set of 1653 actual counts (3 high counts were omitted to reduce the number to 1650 = 10 * 165 and because omitting high counts would not affect the minima). The similarity of this outcome to the previous simulation demonstrates that real differences in examiners' individualization rates contribute little to the measured dispersion.

The final simulation (Fig. S17) investigates whether the real differences among examiners revealed in Fig. S15 pertain more to differences in how many minutiae they marked or to which image pairs they individualized. In order to perform this analysis, we remove subjectivity in the reported counts by measuring a fixed statistic for each image pair, mean(corresponding minutiae). The results show only subtle differences between the actual distribution of minimum(mean(corresponding minutiae)) and the simulated distribution. In other words, the real examiner effect on minimum(corresponding minutiae) relates largely to differences in how the examiners count minutiae, not to differences in the images.

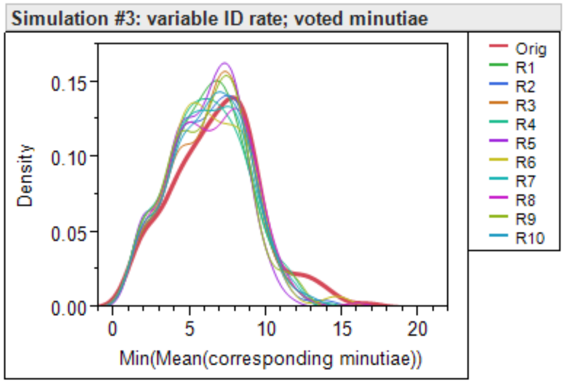

Fig. S17: Simulation #3: variable number of individualizations per examiner, fixed minutia count for each image pair. Ten simulation runs in which the actual mean(corresponding minutia) counts were randomly assigned to 165 simulated examiners; the number of individualizations per simulated examiner matches the actual distribution of individualizations per examiner. In each run, the random reassignments were performed by permuting the original mean counts of the 1653 individualizations. The (bold) reference line shows the actual distribution of Minimum(Mean(corresponding minutiae)). This simulation demonstrates that the real examiner effect on Minimum(corresponding minutiae) relates largely to differences in how the examiners count minutiae, not to differences in the images that they individualized.

In summary, the minimum number of corresponding minutiae required to individualize varied by examiner. More than one-third of examiners individualized with as few as seven or eight minutiae. Most of the observed differences among examiners pertain to the small sample size; that is, more opportunities would have lowered the observed minimum for many examiners. The observed differences in the minimum number of corresponding minutiae relate primarily to differences in the minutia counts that these examiners attributed to the images, not to differences in the images themselves or to differences in the examiners' individualization rates. Examiners do differ substantially in their individualization rates, but differences in their minimum minutia counts do not appear to be an important factor contributing to differing individualization rates.

Appendix SI-20 White Box Latent Examiner Study — Instructions

1 Summary of Test Procedure 2
1.1 Steps to be performed 2
1.2 Latent analysis 3
1.3 Comparison and evaluation 5
2 Markup 7
2.1 Ridge quality painting 7
2.2 Features 9
2.3 Corresponding features 11
3 Analysis and Comparison Determinations 13
3.1 Value determinations 13
3.2 Comparison determinations 14
3.3 Reason for exclusion 14
3.4 Difficulty 15
3.5 Comments 15
4 Image Processing and Markup Controls 15
4.1 Temporary lines 16
4.2 Erase 16
4.3 Show/Hide markup 17
4.4 Tonal reversal 17
4.5 Zoom 17
4.6 Rotation 17
4.7 Grayscale control 17
4.8 Display settings 17
4.9 Shortcuts 18

1 SUMMARY OF TEST PROCEDURE

In the White Box Study, you will be asked to perform twenty-two friction ridge impression examinations. For each examination, you will document your analyses and comparison of the latent and exemplar impressions and record your value and comparison determinations.

This Study is a follow-on to the Black Box Study performed two years ago. In Black Box, we focused on *accuracy* and *reproducibility* of examiners' decisions. In White Box, the focus is on understanding the basis for these determinations. You will be asked to make analysis (value) and comparison determinations for latent-exemplar fingerprint pairs, and to mark the features (quantity and quality) used in making those determinations. The test data will be analyzed to characterize what constitutes sufficient quantity and quality to support a decision.

Anonymity will be assured through the use of examiner IDs and by controlling the flow of information. The analysis data will be kept separate from personally identifiable information (names and email addresses) so that results cannot be associated with specific individuals even by members of the team involved in test administration or analysis. Information associating participants and examiner IDs will be destroyed upon completion of the study. The background survey does not request participants' names, employer, or any other personally identifying information.

Your careful adherence to test guidelines is critical to achieving these objectives: it is important that you apply the same diligence that you use in casework when taking the test.

1.1 Steps to be performed

The White Box test software includes a status window and a comparison tool as shown in Figure 1.

The status window shows the list of examinations to be performed and the status of your work on each. You may work through the examinations in any order. Each examination proceeds through five stages: To Do, Latent in progress (optional), Latent Complete, Comparison in progress (optional), and Comparison complete. To begin or continue an examination, launch the comparison tool by double-clicking on any item in the list.

For each examination, the comparison tool will first present a latent for analysis, then (unless the latent is deemed No Value) the latent and exemplar will be presented together for comparison and evaluation. A copy of your markup and latent value decision will be recorded when you indicate that the latent markup is complete, however, these may be changed during comparison and evaluation.

After all examinations are complete, you will complete a survey and submit your work. Once you have received a confirmation that your work has been received, you will be asked to Remove User Data. The confirmation email will provide instructions on this and how to uninstall the software.

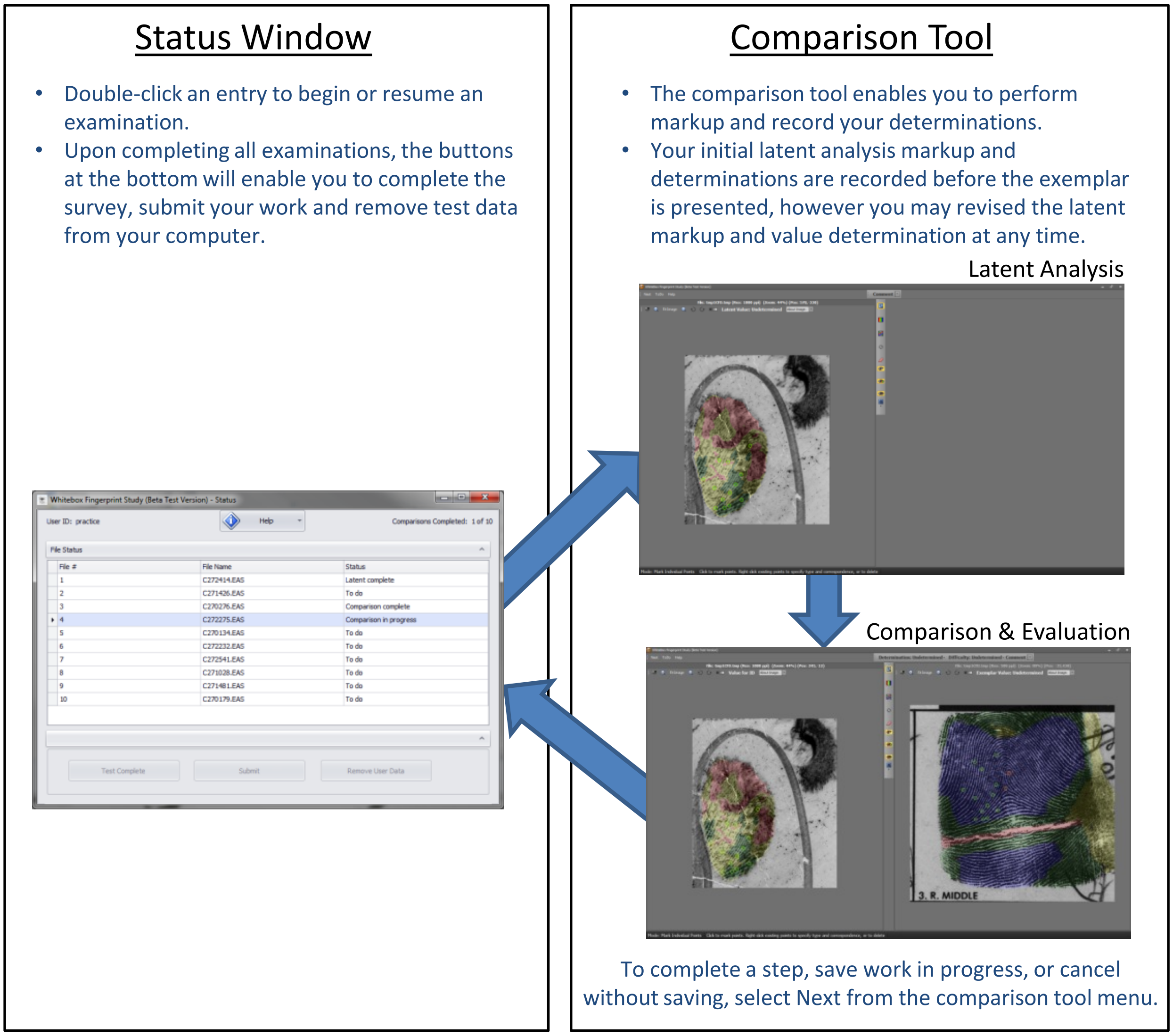

Figure 1: When you launch the White Box application, the status window presents a list of all assigned examinations together with your work status on each. From this window, you will launch the comparison tool to perform each examination. The status window also provides access to help documentation and, at the end of the test, will enable you to complete the survey, submit your work and remove test data from your computer.

1.2 Latent analysis

During latent analysis, you are required to perform the following steps:

- Paint the quality (clarity) of the latent (throughout the entire region of interest).
- Mark all minutiae, cores, and deltas in the latent.
- Record your value determination.

Figure 2 indicates how the comparison tool is used to perform these steps.

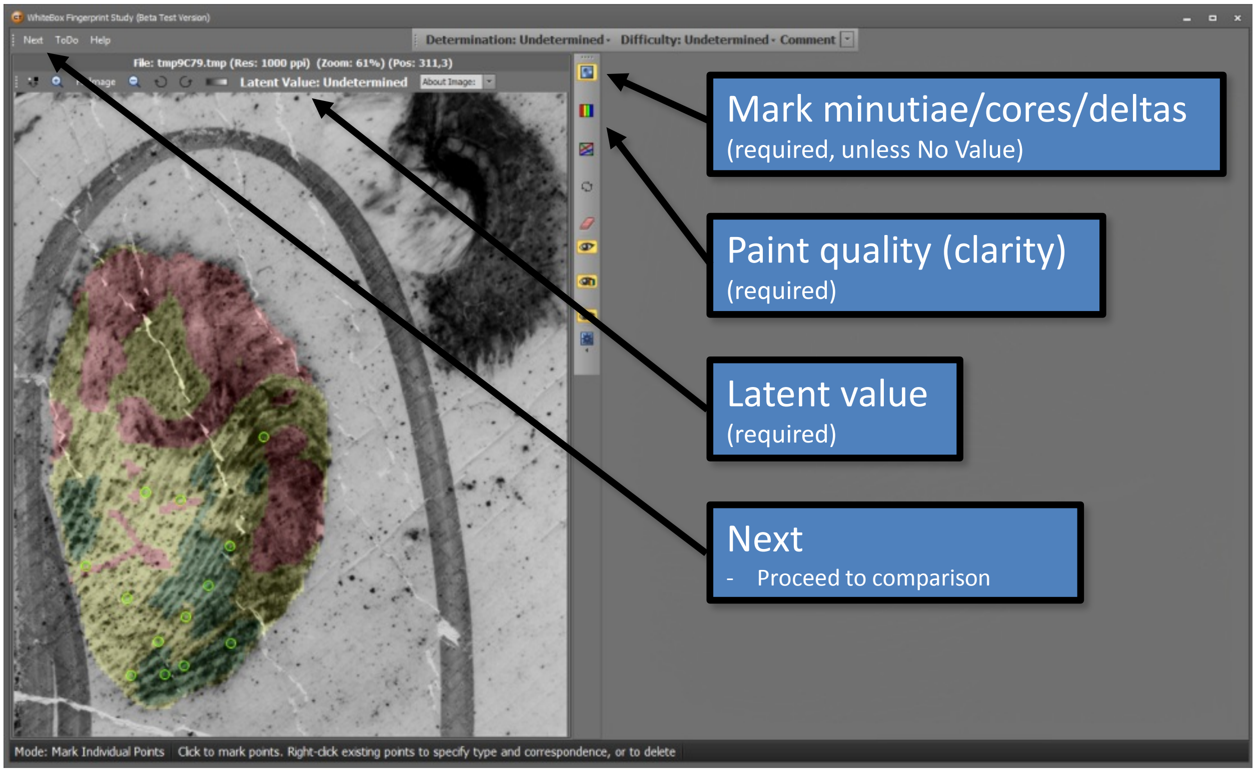

Figure 2: Latent analysis.

After latent analysis, hit Next (or close). You will see a "Close Latent" window (Figure 3) displaying a summary of your markup; warning/errors, if any (such as saying a print is of value without any minutiae marked); and options on saving.

After you have completed latent analysis, you may proceed to comparison and evaluation. Once the exemplar is presented, a copy of your initial latent markup and value determination will be saved for White Box analysis.

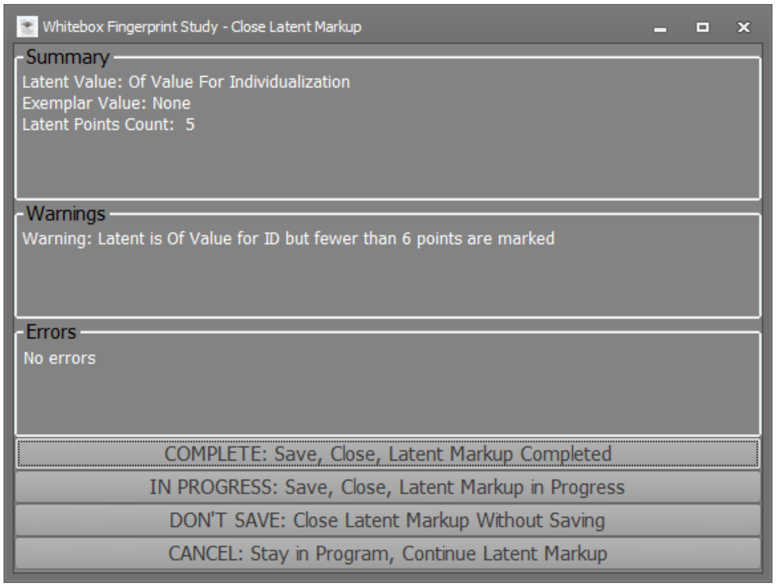

Figure 3: Example of the "Close Latent" window.

1.3 Comparison and evaluation

During comparison and evaluation, you may revise features that you marked during analysis of the latent by adding, deleting or repositioning features, or changing quality markup; you may also change your latent value determination.

During comparison and evaluation, you are required to perform the following steps:

- Paint the quality (clarity) of the exemplar.
- Mark all features in the exemplar that correspond to those marked in the latent.
- Mark any discrepancies used to support an exclusion determination.
- Mark other features whose correspondence (or non-correspondence) you use in making your comparison determination.
- Record your value determination for the exemplar.
- Record your comparison determination.

In the exemplar, you only have to mark correspondences or discrepancies – you do not have to mark every minutia in the exemplar!

Figure 4 indicates how the comparison tool is used to perform these steps.

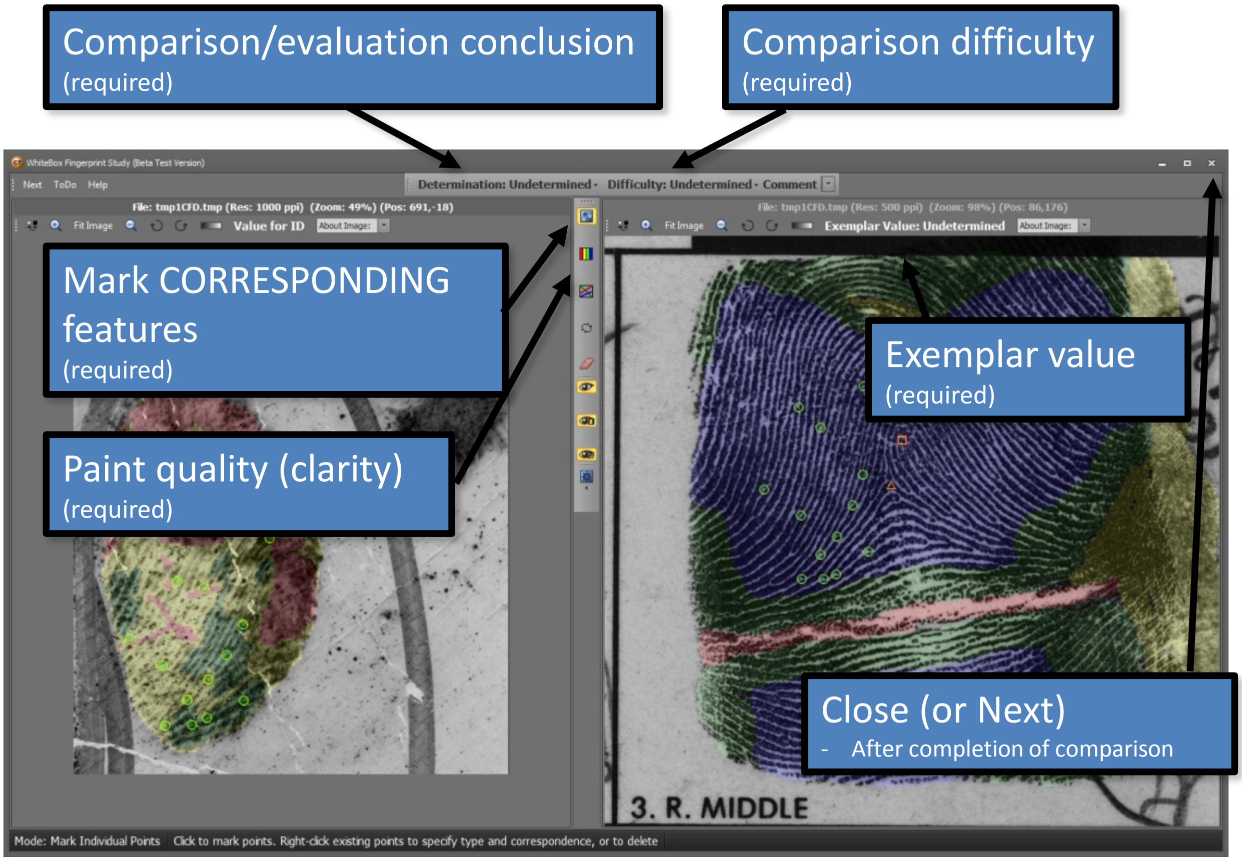

Figure 4: Comparison and evaluation.

When making your determinations, please make the following assumptions:

- Assume that the images provided are the only images available, and that physical evidence, lift cards, fingerprint cards, additional exemplars, and different images of these prints are not available.
- Assume that every impression is a fingerprint, not a palmprint or lower joint.
- For an inconclusive determination, it is assumed that additional exemplars would have been requested and were not available; it is not necessary to state this in a comment.
- Make latent or exemplar value determinations under the assumption that a good-quality exemplar with a large corresponding area may be available for comparison. The specific exemplar shown for comparison may or may not meet these criteria, but that should have no bearing on your value assessment.
- No images have already been claimed "Of Value." In a few images, impressions were marked to indicate which impressions were to be scanned; such marks do not indicate that another examiner necessarily determined that the print is of value.

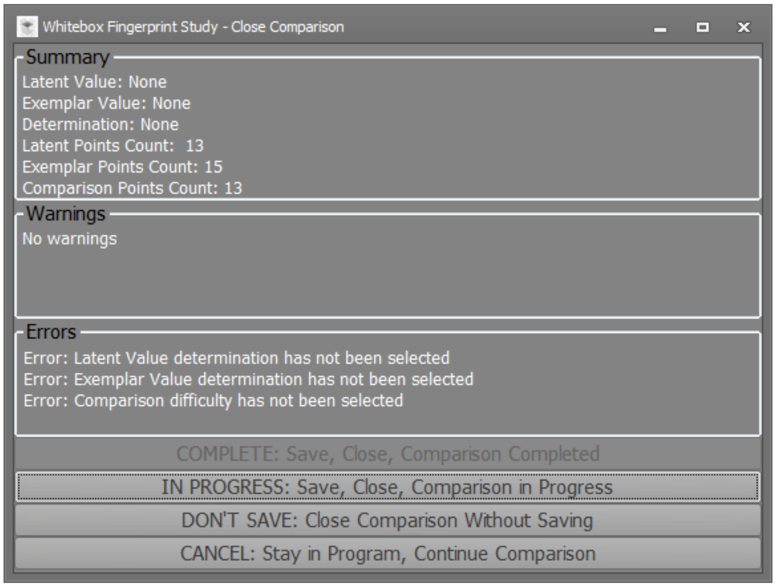

Figure 5: Example of the "Close Comparison" window.

2 MARKUP

Your markup of the fingerprint images is of central interest in this study, which will be looking at the relation between markup and ACE decisions. [1] This markup complies with a subset of features from the Extended Feature Set (EFS), which is the standard for feature markup in the American National Standard ANSI/NIST 2011.

2.1 Ridge quality painting

The ridge quality map is used to document your level of confidence in the marked features. Image quality is documented by painting over the image using standard colors as defined Figure 6. When each image is first presented, the entire image is painted black (denoting background) by default. Paint the all of the impression being evaluated, while leaving the background and other impressions (if any) black. Include all of the impression of interest even when it is superimposed with another impression.

Note particularly two critical distinctions:

- Green (or better) means that you are **certain** of the presence of all of the minutiae you mark in that region AND you are certain that there are no unmarked minutiae.
- Continuous areas marked as yellow or better indicate a single simultaneous impression — any discontinuities (e.g., smears) must be marked in red.

[1] *The ridge quality map and markup of features that you will provide on this test correspond to a subset of the Extended Feature Set (EFS), which is part of the ANSI/NIST-ITL standard (fingerprint.nist.gov/standard/). A detailed discussion of EFS markup including examples is available in Markup Instructions for Extended Friction Ridge Features (http://www.noblis.org/interop).*

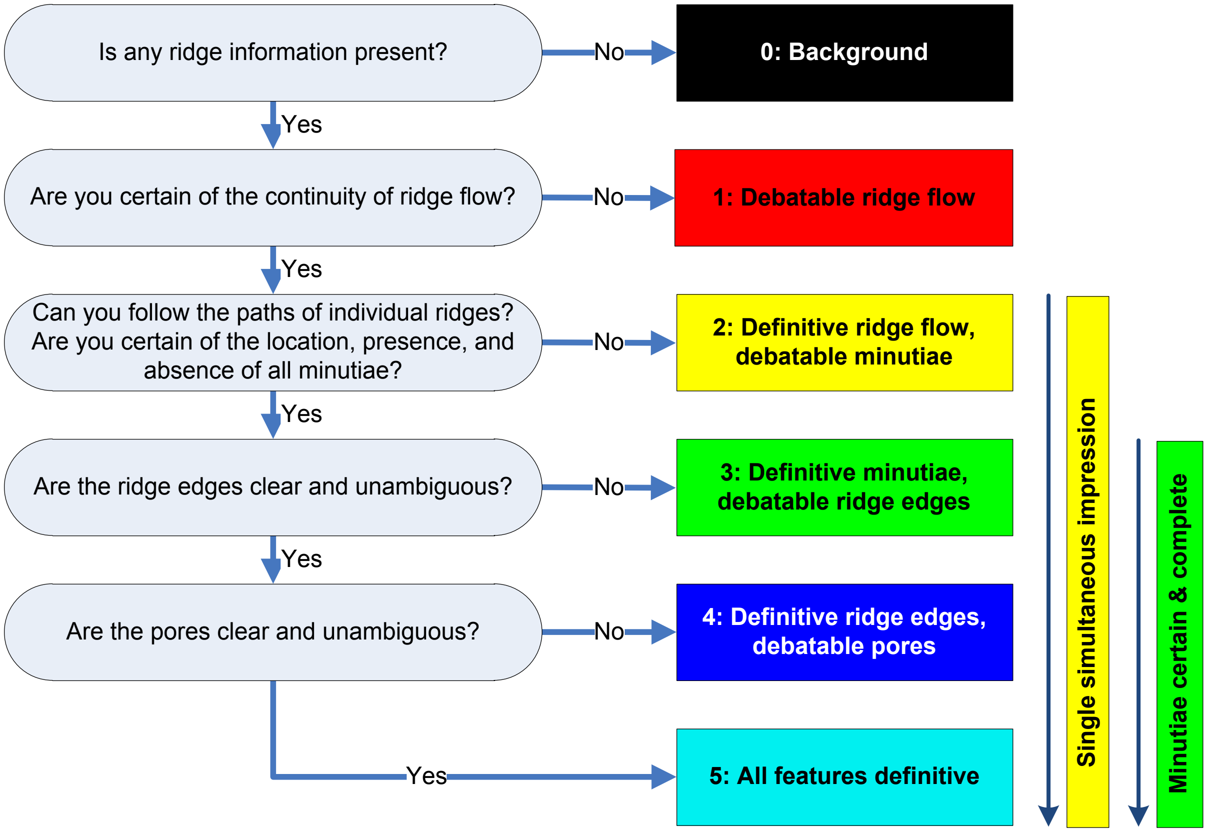

Figure 6: Definitions of ridge quality map colors.

Figure 7 through Figure 9 show examples of ridge quality markup.

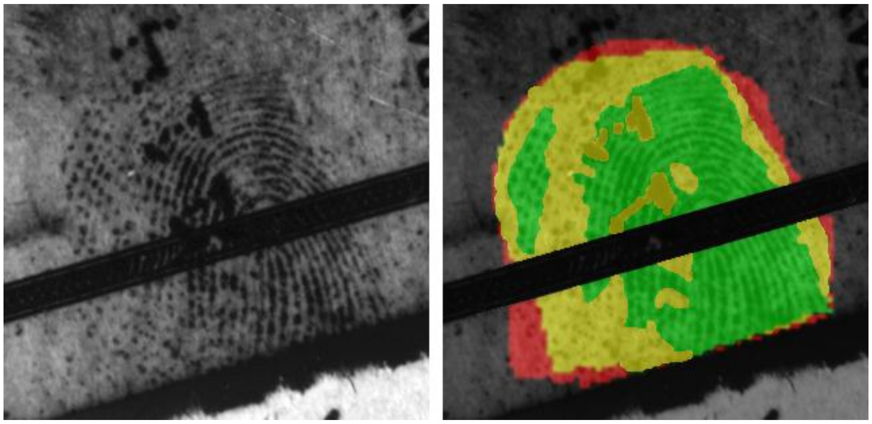

Figure 7: Ridge quality markup on a 500ppi latent.

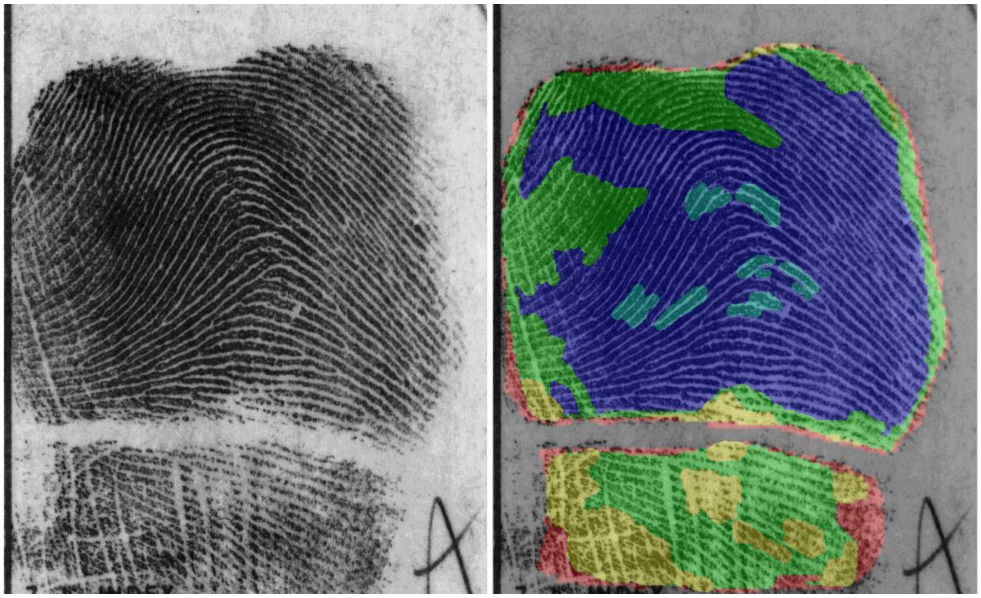

Figure 8: Ridge quality markup on an inked 500ppi exemplar.

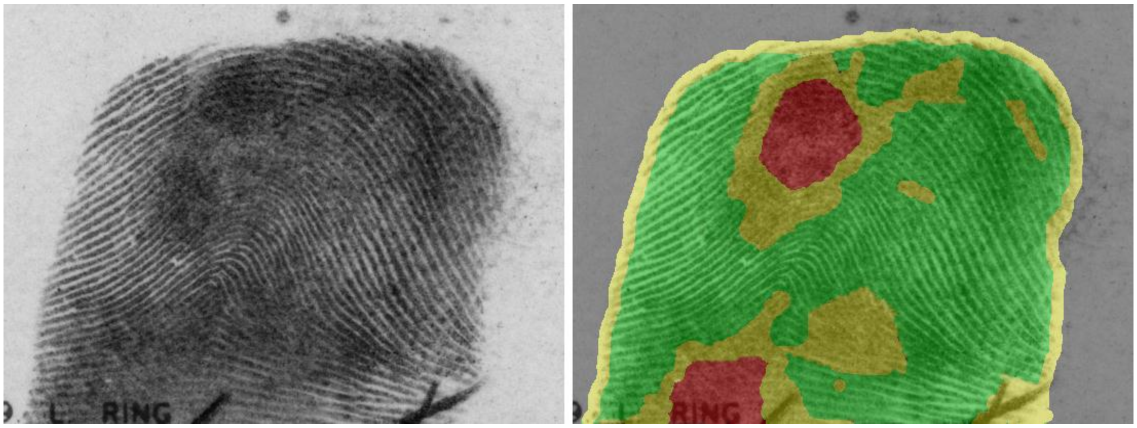

Figure 9: Ridge quality markup on an inked 500ppi exemplar.

How to paint ridge quality:

- Enable painting mode by left-clicking on the Paint Quality Map icon on the vertical toolbar.
- Change the color or size of the paint brush by right-clicking in the image while in Paint Quality Mode.
- To "erase" ridge quality, use black (background) to paint the area.

2.2 Features

When in Mark Points mode, click in the image to mark a feature. By default, the type will be minutia. To indicate a core, delta, or "other" type (such as dots or incipients), you must next right-click the point and make a selection. Cores and deltas should only be marked when they can be accurately located to within approximately three ridge intervals. Marking of "other" features is optional during latent analysis; it is only necessary for features you use as the basis for comparison determinations.

Minutiae

- The location for a **bifurcation** shall be at the "Y" of the ridge.

- The location for a **ridge ending** or **unknown type** shall be at the "Y" of the valley. Note that this definition of ridge ending location corresponds to markup for IAFIS (and NGI), but differs from some vendor-specific approaches, which may mark ridge endings on the ridge itself.

Cores are marked at the focus of the innermost recurving ridge. Note that the core is not on the innermost recurving ridgeline itself.

Deltas are marked at the center of the triradius. Deltas are marked in fingerprints for loops, whorls, and (when present) in tented arches.

Other may be used to mark features such as scars, dots, incipient ridges, creases and linear discontinuities, ridge edge features, or pores.

Direction (theta) does not need to be indicated for minutiae, cores, or deltas.

Remember that quality painting is used to indicate confidence in the presence, absence and location of minutiae:

- Green indicates that you are certain that each and every minutia in the area is marked;
- Yellow indicates that you are not confident in the presence or location of marked minutiae and there may be minutiae in the area that you did not mark.

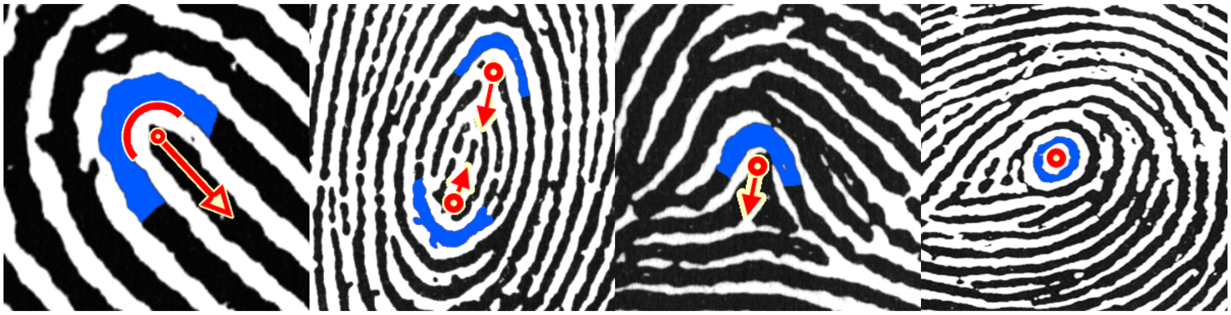

Figure 10: Cores are marked at the focus of the innermost recurving ridgeline. Examples of core locations for a right slant loop, plain whorl, tented arch, and central pocket loop whorl.

How to mark features:

- Enable feature marking by left-clicking on the Mark Points icon on the vertical toolbar.
- To mark a feature (core, delta, minutia, other) on an image, left-click at that position on the image. By default, the feature type will be minutia; to specify core, delta, or other, right click on the feature and select the appropriate type.
- To delete a marker, right-click and select "Delete Point."
- Change the color or size of markers by clicking the Display Settings button.

2.3 Corresponding features

You can indicate that two features correspond by clicking to select a feature in one image, then clicking a previously marked feature or a new point in the other image as illustrated in Figure 11. When you assess the correspondence of features during comparison, indicate the type of correspondence as summarized in Table 1. Correspondence applies to any type of feature (minutia, core, delta, other).

Type of correspondence	Description / What to mark	How to mark
Definite correspondence<br>(both images)	The feature is definitely visible in both images.<br><br>For each feature marked in the latent, mark the corresponding feature if present in the exemplar. Mark any additional features you use in making your determination (such as incipients or level-3 detail).	Click to select a point in one image, then click a preexisting or new point in the other image.
Definite non-correspondence<br>(single image)	A discrepancy – the feature exists in one print and is definitely not present in the other print.<br><br>Indicate points in one print that definitely do not exist in the other print as needed to support an exclusion determination	Right-click a point and select "Definite non-correspondence". Feature is displayed with "X".
Inconclusive correspondence<br>(single image)	The feature is not visible in the other print (either obscured, or outside the corresponding area)	Mark a point in one image (and do nothing else).
Debatable correspondence<br>(both images)	Optional: An apparent correspondence that does not rise to the threshold of definite correspondence.<br><br>Points that potentially correspond, but do not meet your threshold for supporting an ID, should be marked as debatable. This may occur, for example, when the determination is inconclusive or in low-clarity areas. In exclusion determinations, use debatable correspondence to mark reference points used to establish a discrepancy	After marking two points as corresponding, right-click either point, then select "Debatable correspondence." Feature is displayed with "?".

Table 1: Types of correspondence

- Move the cursor over a point – it will grow.
- Select the point by clicking on it – it will now stay large after you move the mouse.

- Move the cursor over the corresponding point in the other image – it will grow.

- Click the point – both points will now change colors.

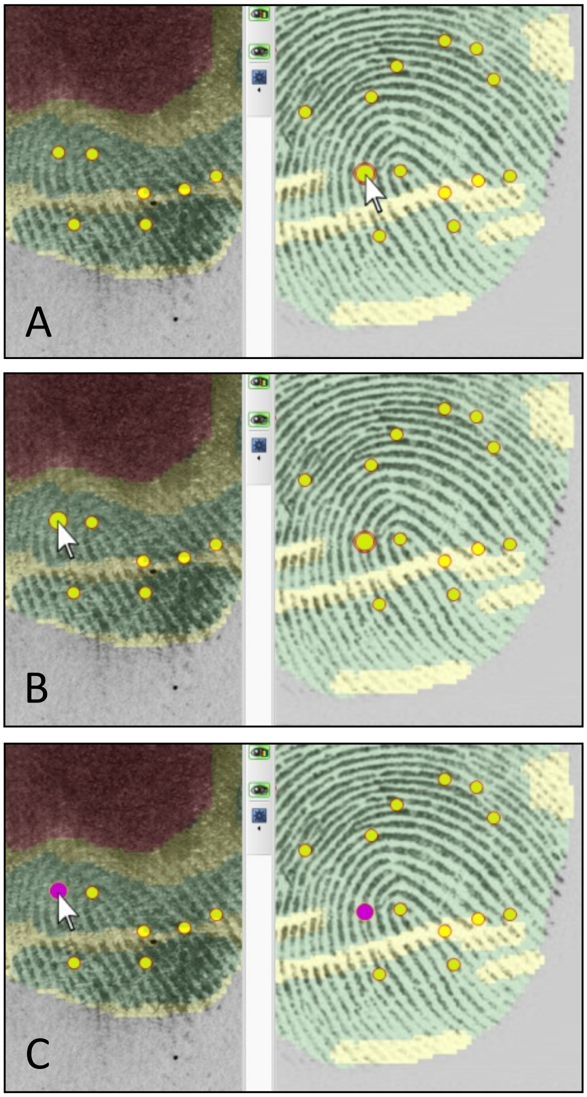

Figure 11: Marking corresponding points. Once corresponding points have been marked, moving the cursor over one point will cause it and the corresponding point to grow; this can be useful in reviewing correspondences. To break a correspondence, right-click on one point and select "unmatch point."

How to mark corresponding features:

- Left-click an existing point to select it; notice that the selected point will be larger than the others. Then click the corresponding point in the other image. The color of the two points will change to indicate that they (definitely) correspond.
- To indicate a debatable correspondence between two points, first mark the points as corresponding, then right-click either of the points and select "Debatable Correspondence" from the context menu.
- To indicate that a point definitely does not exist in the other image, first mark the point (left-click), then right-click on the marker and select "Definite Non-correspondence" from the context menu.
- Change the color or size of markers by clicking the Display Settings button.

3 ANALYSIS AND COMPARISON DETERMINATIONS

The determinations as defined here (Table 2, Table 3) may not correspond precisely to the procedures you normally follow. Please try to follow the guidance in this section as closely as possible so that responses from different people/organizations are comparable.

3.1 Value determinations

You must indicate the value of each image (latent or exemplar).

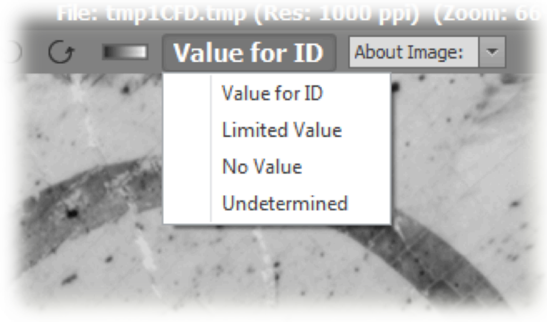

Determination	Definition
Of value for individualization *(abbreviated "Value for ID")*	The impression is of value and is appropriate for potential individualization if an appropriate exemplar is available.
Of value for exclusion only *(abbreviated "Limited value")*	The impression is NOT of value for individualization. The impression contains some friction ridge information (level 1 and/or level 2) that may be appropriate for exclusion if an appropriate exemplar is available.
No Value	The impression contains insufficient friction ridge information to reach a conclusion.

Table 2: Latent value assessment

3.2 Comparison determinations

For each comparison you must indicate a comparison determination (does not apply to latents that you determined were or no value).

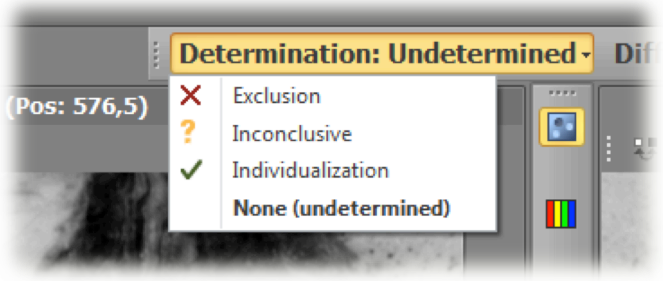

Determination	Definition
Individualization	The two fingerprints originated from the same finger.
Inconclusive	Neither individualization nor exclusion is possible.
Exclusions (of finger)	The two fingerprints did not come from the same finger.

Table 3: Comparison determination

3.3 Reason for exclusion

When the comparison determination is exclusion (of finger), you will be asked to indicate what observed differences led to that determination (Table 4). Please select the **first** option that applies.

Basis for exclusion	Definition
Pattern classes differ	The exclusion could be made based on pattern class alone.
Core or delta differences	The exclusion could be made based on one or more of the following: differing ridge flow in the cores or deltas; differing core-delta ridge counts; or differing relations among the deltas.
One or more minutiae differ	The exclusion determination could be made based on a comparison of Level-2 information.
Level 3 features differ	The exclusion determination required comparison of Level-3 information.
Other	None of the above categories satisfactorily explains the basis for the exclusion. Please briefly indicate the basis for the exclusion.

Table 4: Exclusion reason (provided only when the comparison determination is exclusion)

3.4 Difficulty

For each comparison, indicate how difficult the comparison was. Routine comparisons should be indicated as "Moderate".

Determination	Definition
Very Easy/Obvious	The comparison determination was obvious.
Easy	The comparison was easier than most latent comparisons.
Moderate	The comparison was a typical latent comparison.
Difficult	The comparison was more difficult than most latent comparisons.
Very Difficult	The comparison was unusually difficult, involving high distortion and/or other red flags.

Table 5: Comparison difficulty

3.5 Comments

For each examination, a comment box is provided to allow you to communicate concerns about the test process to the test administrators. Examples include software issues, data entry errors, particularly problematic images, any problems taking the test. The comment box is ***not*** intended to routinely capture your thought process in reaching determinations: comments should be reserved for exceptional circumstances. General comments about the White Box study will be solicited in the participant survey.

4 IMAGE PROCESSING AND MARKUP CONTROLS

The comparison tool interface is organized into several regions: latent (left side), exemplar (right side), image enhancement functions (above each image), markup functions (vertical toolbar between the two images), categorical test responses (above center), and a menu bar (at the top). A short descriptor of each button can be revealed by positioning the cursor over that button.

Figure 12 identifies software features that were not previously described.

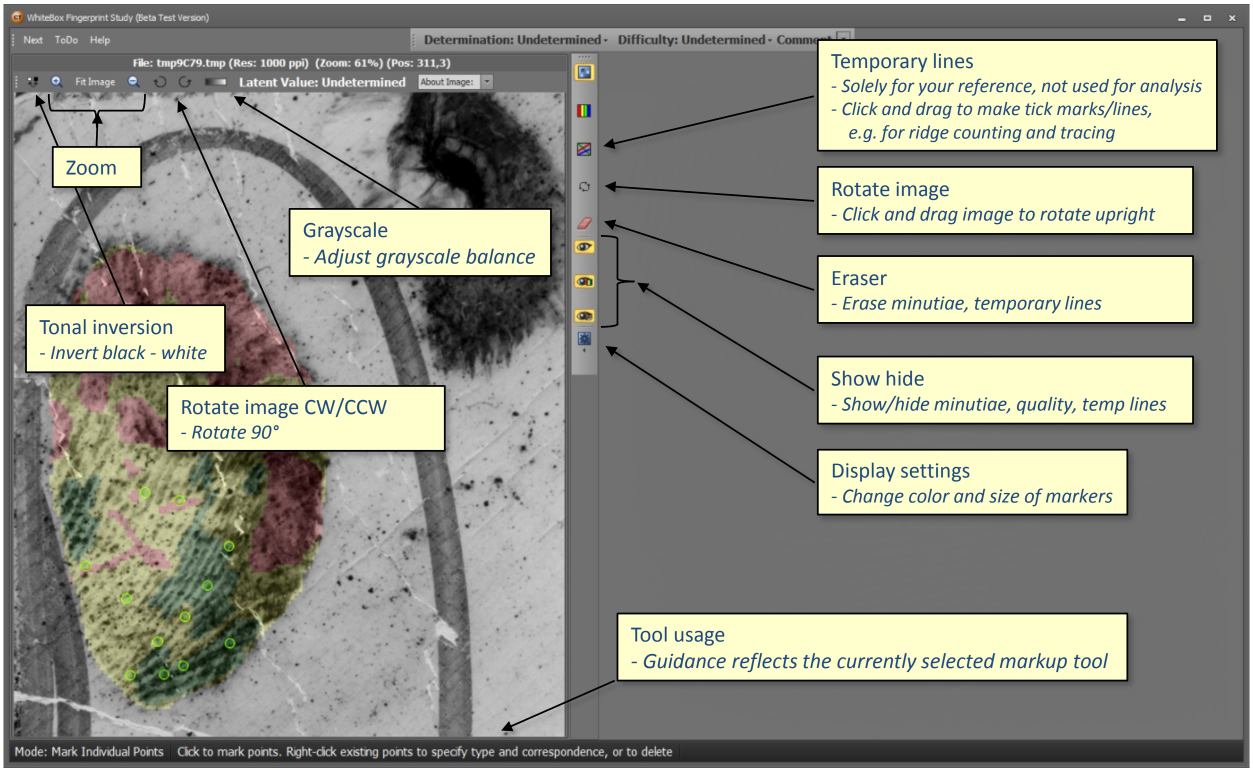

Figure 12. Image processing and markup tools. Controls specific to either the latent or exemplar are located above each image; controls on the vertical (center) toolbar apply to both images. During markup, the comparison tool will always be in one of five modes: mark points, paint quality, draw lines, dynamic rotate, or erase. Controls to select the current mode and to set display preferences are located on the vertical toolbar. Depending on the current mode and the location of the cursor, right-clicking will display a context-sensitive menu of additional options.

Painting ridge quality, marking of features, and marking of corresponding features were discussed previously; see

- Section 2.1 (Ridge quality).
- Section 2.2 (Features).
- Section 2.3 (Corresponding features).

4.1 Temporary lines

- Enable line drawing mode by left-clicking on the Draw Lines icon on the vertical toolbar.
- This function allows users to draw lines on the image, which may be helpful for ridge counting or tracing. This tool is provided as a convenience; the lines will be ignored during analysis of test results.

4.2 Erase

- Enable erasing mode by left-clicking on the Erase icon on the vertical toolbar. This tool erases marked features and temporary lines.
- With the left mouse button depressed, drag the eraser over the features or lines to be erased. Matched features will become unmatched if one of the corresponding features is erased. Note that this will not erase the corresponding feature; it will simply remove the corresponding status.

4.3 Show/Hide markup

- Three separate controls are provided to toggle whether points (marked features), quality map (painting), and temporary lines are displayed on the image(s).

- You can show/hide minutiae by hitting the spacebar (or by using the show/hide minutiae button in the middle toolbar).
- You can show/hide the quality markup by hitting ctrl-q (or by using the show/hide quality button in the middle toolbar)
- You can change the marker size, color and fill by using the Display Settings button in the middle toolbar.

4.4 Tonal reversal

- Inverts the colors in the image, so that black becomes white and white becomes black.

4.5 Zoom

The latent image and the exemplar image are linked so that when a zoom function is clicked, both images react. There are three different zoom controls:

- Zoom In or Out — Click either the "+" or the "–" buttons next to the Zoom button to zoom in or out on both images.
- Fit image — Fit the current image (latent or exemplar) to the window.

4.6 Rotation

- Fixed Rotation (above each image) – Rotate image 90 degrees
- Arbitrary Rotation (vertical toolbar) – Rotate image with hand cursor while holding down left mouse button.

4.7 Grayscale control

- Grayscale enhancement settings are reversible and not saved.
- The slider adjusts the grayscale balance of the image.
- The white and black markers define the lightest and darkest levels shown in the image.
- The gray marker defines Gamma, which defines the distribution of gray tones in the image

4.8 Display settings

Allows changing of the colors and sizes used to display features.

4.9 Shortcuts

Function	Shortcut
Zoom	Ctrl+ and Ctrl-<br>Or roll the mouse wheel
Show/hide point features	Space bar
Show/hide lines	Ctrl-L
Show/hide quality map	Ctrl-Q

In addition, the right-click menu includes a "Center Here" option as an alternative to using the scrollbars in positioning the images.
